# Structural insights into an atypical secretory pathway kinase crucial for *Toxoplasma gondii* invasion

**DOI:** 10.1038/s41467-021-24083-y

**Published:** 2021-06-18

**Authors:** Gaëlle Lentini, Rouaa Ben Chaabene, Oscar Vadas, Chandra Ramakrishnan, Budhaditya Mukherjee, Ved Mehta, Matteo Lunghi, Jonas Grossmann, Bohumil Maco, Rémy Visentin, Adrian B. Hehl, Volodymyr M. Korkhov, Dominique Soldati-Favre

**Affiliations:** 1 Department of Microbiology and Molecular Medicine, University of Geneva, Geneva, Switzerland; 2 Institute of Parasitology, University of Zurich, Zurich, Switzerland; 3 Institute of Biochemistry, ETH Zurich, Zurich, Switzerland; 4 Functional Genomic Center Zurich, ETH Zurich and University of Zurich, Zurich, Switzerland; 5 The Swiss Institute of Bioinformatics, SIB, Lausanne, Switzerland; 6 Paul Scherrer Institute, Villigen, Switzerland; 7 Present Address: School of Medical Science and Technology, IIT Kharagpur, India

**Keywords:** Kinases, Proteomics, Parasite biology, Cryoelectron microscopy

## Abstract

Active host cell invasion by the obligate intracellular apicomplexan parasites relies on the formation of a moving junction, which connects parasite and host cell plasma membranes during entry. Invading *Toxoplasma gondii* tachyzoites secrete their rhoptry content and insert a complex of RON proteins on the cytoplasmic side of the host cell membrane providing an anchor to which the parasite tethers. Here we show that a rhoptry-resident kinase RON13 is a key virulence factor that plays a crucial role in host cell entry. Cryo-EM, kinase assays, phosphoproteomics and cellular analyses reveal that RON13 is a secretory pathway kinase of atypical structure that phosphorylates rhoptry proteins including the components of the RON complex. Ultimately, RON13 kinase activity controls host cell invasion by anchoring the moving junction at the parasite-host cell interface.

## Abstract (web summary)

Host cell invasion by *Toxoplasma gondii* depends on the heavily phosphorylated RON complex, but the relevance and regulation of these modifications are not understood. Here, the authors identify the kinase RON13 as a key virulence factor, determine its structure and show that it phosphorylates the RON complex.

## Introduction

Toxoplasmosis is a zoonotic infectious disease caused by the protozoan parasite *Toxoplasma gondii*, a member of the Apicomplexa phylum. Specialized apical secretory organelles called rhoptries and micronemes crucially participate in gliding motility and active host cell entry of these obligate intracellular parasites. Micronemes secrete surface-exposed transmembrane adhesins (MICs) linked to the actomyosin system that promote traction for motility and invasion. Rhoptries inject proteins across the host cell plasma membrane (PM)^1^, some acting as receptors during host cell invasion and others as effectors to subvert host cellular functions^2^. These club-shaped organelles contain rhoptry bulb proteins (ROPs) in the enlarged base that are segregated from rhoptry neck proteins (RONs) at the thin neck connected to the apical tip of the parasite. After injection, the RON complex (composed of RON2, 4, 5, and 8) is inserted in the host PM and binds to the microneme protein AMA1 on the parasite surface, forming a multiprotein complex that contributes to the formation of the moving junction (MJ)^3,4^. On the host cytoplasmic side, the RON complex interacts with several host adaptor proteins presumably linking the MJ with the host cortical actin^5^. Powered by the parasite actomyosin system^6^, the MJ translocates toward the posterior end of the invading parasite, where the PM-derived parasitophorous vacuole membrane (PVM) is sealed^7,8^. Of relevance, the rhoptries also discharge membranous materials that participate in the PVM formation^9^. Once inside the host cell, *T. gondii* tachyzoite survival relies on an expanded coccidian lineage-specific family of secreted ROP kinases and pseudo-kinases (ROPKs)^10,11^ acting as key virulence factors^12^. The RON complex is heavily phosphorylated^13^; however, the functional relevance of this posttranslational modification and the kinase(s) implicated are not known. Here, we identified and characterized a rhoptry-resident kinase RON13 that phosphorylates several rhoptry proteins, including the RON complex. RON13 kinase activity stabilizes the RON complex at the MJ to ensure successful invasion.

## Results

### Proteolytic maturation of RON13

**Fig. 1:**
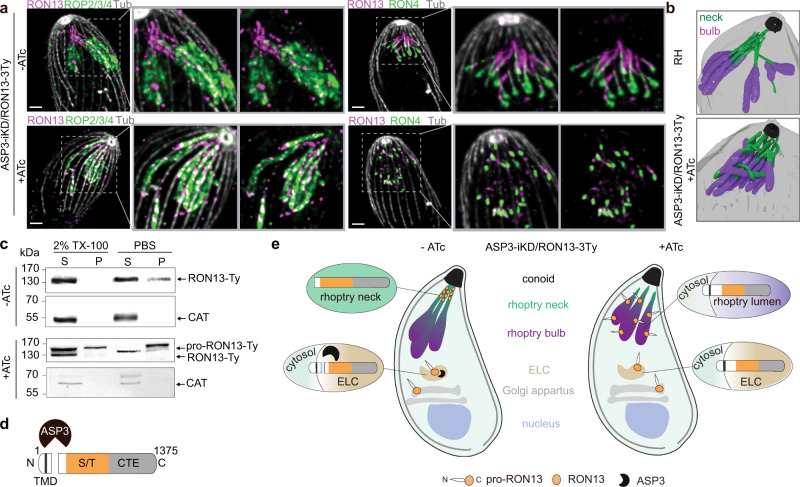
RON13 is a RON kinase processed by ASP3. **a** U-ExM images of rhoptries from ASP3-iKD/RON13-3Ty extracellular parasites ± anhydrotetracycline (ATc). RON4 (green) and ROP2/3/4 (green) antibodies are used to visualize the neck and the bulb of the rhoptries, respectively. RON13-3Ty (magenta) is detected by anti-Ty antibodies. The subpellicular microtubules (gray) are stained with α/β anti-tubulin antibodies. Scale bar = 2 µm. Image representative of three biologically independent experiments. **b** 3D reconstruction from FIB-SEM images of the apical part of RH (control) and ASP3-iKD parasites treated 48 h with ATc. The neck (green) and the bulb (violet) of the rhoptries are colored. The conoid (black) and the PM (gray) are depicted. *n* = 1 biologically independent experiment. **c** Solubility of RON13 in ASP3-iKD/RON13-3Ty parasites ±ATc. Catalase is a marker of the soluble fraction. Samples derived from the same experiment and gels were processed in parallel. Image representative of three biologically independent experiments. **d** Scheme of RON13 protein and its cleavage by ASP3 just downstream the transmembrane domain (TMD). S/T serine/threonine kinase domain (orange), CTE C-terminal extension (gray). **e** Scheme representing the fate of RON13 in presence or in absence of ASP3. Without processing by ASP3, RON13 remains insoluble and mistargeted to the body of morphologically aberrant rhoptries. ELC endosome-like compartment. Source data are provided as a Source data file.

Most ROPs/RONs as well as MICs are synthetized as pre-pro-proteins and are processed in the endosomal-like compartment by the aspartyl protease ASP3 ref. ^14^. ASP3-depleted parasites are unable to invade host cells, notably due to a severe defect in rhoptry discharge. Among the identified ASP3 substrates^14^ the TGGT1_321650 gene product (here referred to as RON13) is predicted to be a kinase. RON13 localizes to the rhoptry neck^14^ and deletion of the gene is fitness-conferring based on a genome-wide CRISPR-Cas9 screen^15^. Ultrastructure expansion microscopy (U-ExM) revealed that RON proteins present two types of localizations, previously undistinguishable by conventional microscopy. RON2 and RON4 are found all along the neck of the organelles, while RON13 and RON9 localize at the extreme tip of the rhoptry neck (Fig. 1a and Supplementary Fig. 1a, b). Upon ASP3 depletion, the localization of RON13 and other RONs is altered with a disappearance of the neck and sliding of the bulbous part of the organelle closer to the conoid (Fig. 1a and Supplementary Fig. 1a, b). Focused ion beam scanning electron microscopy (FIB-SEM) analysis confirmed that the rhoptry necks are morphologically aberrant and do not extend to the apical tip of the parasite (Fig. 1b, Supplementary Fig. 1b, and Supplementary Movies 1 and 2). RON13 is a substrate of ASP3 and consequently, in the absence of the protease, RON13 is not processed anymore and accumulates as a membrane-anchored, largely insoluble pro-protein (Fig. 1c, d and Supplementary Fig. 1c). Taken together the aberrant rhoptry morphology and content organization in ASP3-depleted parasites provide a rationale for the previously reported defect in rhoptry discharge^14^ (Fig. 1e).

**Fig. 2:**
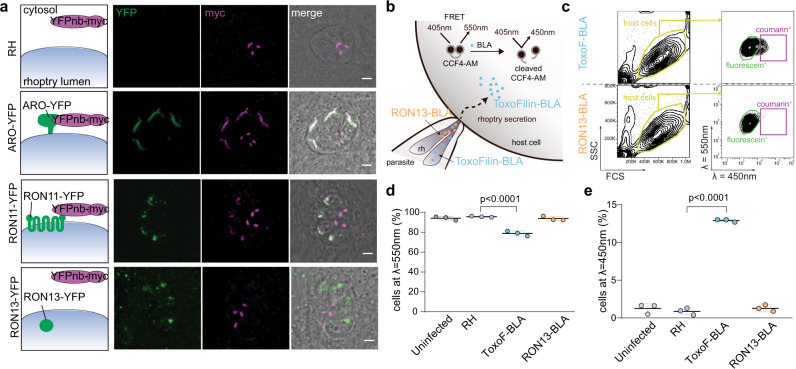
RON13 is a luminal rhoptry protein that is not secreted during invasion. **a** IFAs of RH-, ARO-YFP- (green), RON11-YFP- (green), or RON13-YFP- (green) expressing parasites transiently transfected with cytosolic nanobodies targeting YFP fused to a myc-tag (YFPnb-myc). ARO is a protein associated with the cytosolic face of the rhoptry membrane and RON11 is a type III transmembrane protein with its C-terminal domain exposed in the parasite cytoplasm. The myc signal (magenta) observed at the basal part of the parasite is unspecific. Left panels show the schematic topology of the proteins and their ability to bind the cytosolic YFPnb-myc. Scale bar = 2 μm. Image representative of three biologically independent experiments. **b** Principle of the experimental design for the FRET-based rhoptry secretion assay used to determine if RON13 (orange) is secreted into the host cell during the invasion. Toxofilin (blue) is a soluble rhoptry protein secreted into the host cell. **c** Gating strategy for quantification of fluorescein^+^ cell (green gate; *λ* = 550 nm) and coumarin^+^ cell (violet gate; *λ* = 450 nm) frequency for RON13-BLA- and Toxofilin-BLA-infected cell monolayer (yellow gate) analyzed by flow cytometry. **d**, **e** Frequency of fluorescein^+^ cells (*λ* = 550 nm, **d**) or coumarin^+^ cells (*λ* = 450 nm, **e**) in each condition (mean ± SD; *n* = 3 biologically independent experiments). Statistical significance was assessed by a one-way ANOVA significance with Tukey’s multiple comparison. Source data are provided as a Source data file.

The topology of the RON13 at the rhoptry membrane was examined in RON13-YFP-expressing parasites. Processed RON13 localizes to the rhoptry lumen as demonstrated by the lack of detection of RON13-YFP by cytosolic anti-YFP nanobodies (Fig. 2a and Supplementary Fig. 2a). To determine if RON13 is secreted, RON13 was fused to beta-lactamase (RON13-BLA) and intracellular lactamase activity was used as a sensitive readout for rhoptry secretion in the host cell^16^. Cells infected by RON13-BLA parasites do not show any detectable lactamase activity (Fig. 2b–e and Supplementary Fig. 2b–e). In addition, RON13 is neither associated with e-vacuoles nor with the RON complex post-secretion in invading parasites, supporting the notion that RON13 is not secreted into the host cell during invasion but is instead active in the parasite (Supplementary Fig. 2f, g).

### RON13 is an active kinase

**Fig. 3:**
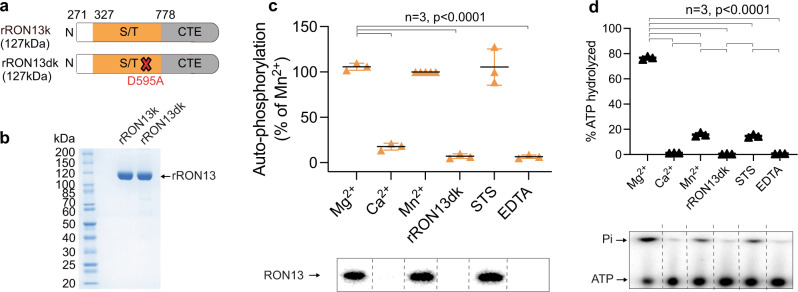
Recombinant RON13 is active in vitro. **a** Scheme of recombinant RON13 kinase (rRON13k) and RON13 dead kinase (rRON13dk), in which kinase activity is abrogated by introducing the mutation D595A. **b** Coomassie-stained gel of purified rRON13k and RON13dk produced in insect cells. **c** Radioactive RON13 kinase activity assays based on rRON13k autophosphorylation. Radiograph is shown below the graph. Densitometry data (mean ± SD; *n* = 3 biologically independent experiments) were normalized to rRON13k in presence of Mn^2+^. Statistical significance was determined using a two-tailed paired *t* test. STS staurosporine. **d** ATP hydrolysis during kinase reactions, as measured by TLC analysis (mean ± SD; *n* = 3 biologically independent experiments). Autoradiograph showing both non-hydrolyzed ATP (ATP) and inorganic phosphate (Pi) is shown below the graph. *P* values were determined by a two-tailed paired *t* test. Source data are provided as a Source data file.

RON13 is a 153 kDa type II transmembrane protein with a putative serine/threonine kinase domain and a large luminal carboxy-terminal extension (CTE) with no identifiable structural motif (Fig. 1d). Phylogenetic analysis showed that RON13 belongs to the ROPK clade (Supplementary Fig. 3)^11^. It has a bilobal kinase domain organization and possesses the motifs characteristic of active eukaryotic protein kinases (ePKs; Supplementary Data. 1). The canonical HRD catalytic triad of ePKs differs in RON13 with an HFD motif conserved among all the coccidian orthologues of RON13 (Supplementary Data 2). RON13 is an active kinase as demonstrated by the intense autophosphorylation activity of the recombinant RON13 protein (rRON13k; Fig. 3a–c). The kinase activity is stimulated by the addition of Mg^2+^ or Mn^2+^, and is insensitive to the broad-spectrum kinase inhibitor staurosporine (Fig. 3c), similarly to the Fam20C secretory pathway kinase^17^. In addition, ATP hydrolysis activity was detected (Fig. 3d). The catalytic activity was completely abolished by introducing a single amino acid change (D595A) in the catalytic HFD motif (rRON13dk), validating the importance of this conserved kinase motif for enzymatic activity (Fig. 3a–c).

### RON13 has an atypical kinase structure

**Fig. 4:**
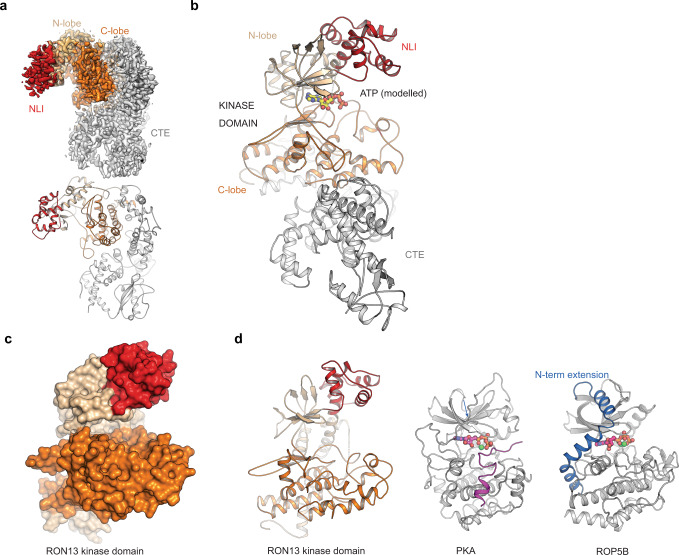
Structure of RON13. **a** Cryo-EM density map determined by single-particle analysis at a resolution of 3.1 Å (top), and the corresponding views of the atomic model of rRON13dk. The kinase domain and the C-terminal extension (CTE) are colored in orange and gray tones. **b** Domain organization of RON13. The position of the ATP-binding site is indicated by the ATP ligand modeled into the active site (absent in the experimentally determined density map). The N-lobe insertion (NLI, red) and the C-terminal extension (CTE, gray) are color-coded. **c** Surface representation of RON13 kinase domain at 3.1 Å resolution. **d** Comparison of RON13 kinase domain structure with other kinases. PKA from *Mus musculus* (PDB ID: 1atp) is complexed with MnATP and an inhibitory peptide (violet). ROP5B from *T. gondii* bound to ATP (PDB ID: 3q60) contains a ROP-specific N-terminal extension (blue).

To gain insight into the function and regulation of RON13 kinase, its three-dimensional structure was resolved at 3.1 Å resolution by single-particle cryo-EM (Fig. 4a, Supplementary Fig. 4a–i, Supplementary Table 1, and Supplementary Movie 3). The RON13 kinase domain is similar to ePKs, but contains an α-helical N-lobe insertion (NLI; Fig. 4b, c) present exclusively in RON13 coccidian orthologues (Supplementary Data 1 and 2). Unlike ROPKs that also reside in the rhoptries, RON13 lacks an α-helical N-terminal extension (Fig. 4d)^10,18^. The kinase C-lobe structure contains all the motifs required for ATP transfer to the substrate, as well as a ROPK-specific disulfide bond^10^. Another unique feature of RON13 is its CTE, composed of 550 amino acids, that shares no sequence homology with any other proteins (Supplementary Data 1 and 2). The CTE is mainly composed of α-helices clamping the kinase domain C-lobe (Fig. 4b). Of note, the CTE is essential for RON13 folding and stability as neither expression of the kinase domain alone, nor proteolytic cleavage to separate the kinase domain from the CTE of rRON13 yielded a functional RON13 kinase. Although the proteolytic cleavage was efficient, as observed on denaturing/reducing SDS–PAGE analysis, both the kinase domain and the CTE remained attached and could not be separated by addition of high salt concentrations. Cleaved rRON13 behaved exactly as WT rRON13 when analyzed by size-exclusion chromatography, further emphasizing the strong interaction between RON13 kinase domain and the CTE (Supplementary Fig. 5a, b). Analysis of RON13 electrostatic potential highlighted a rather extended positively charged groove lying on one side of the kinase that may be involved in protein–protein interactions, but did not provide strong hints to the role the CTE plays (Supplementary Fig. 5c).

### RON13 is critical for invasion and virulence

**Fig. 5:**
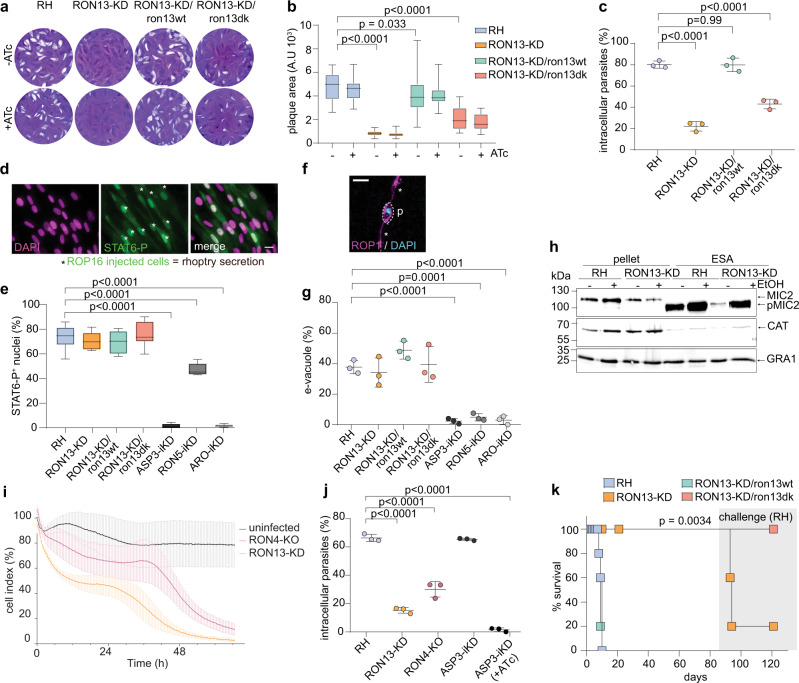
RON13 is critical for invasion. **a** Plaque assays of different parasite strains (±ATc) showing that depletion of RON13 impairs the parasite lytic cycle (no plaque), a phenotype that is fully rescued by complementation with an active RON13 kinase. Image representative of three biologically independent experiments. **b** Quantification of plaque assays for RH, RON13-KD, and complemented RON13-KD/ron13wt or RON13-KD/ron13dk parasites (±ATc). Data are presented as box and whiskers plot (median with min to max, *n* = 3 biologically independent experiments). Statistical significance was assessed by a two-way ANOVA significance with Tukey’s multiple comparison. **c** Invasion assay (+ATc) showing a strong invasion defect when RON13 kinase is absent or inactive *P* value were determined by a one-way ANOVA significance with Tukey’s multiple comparison. (Mean ± SD; *n* = 3 biologically independent experiments). **d** IFA showing a representative field of the rhoptry secretion test using phosphor-STAT6 as a readout with fibroblast nuclei stained in DAPI (magenta) and ROP16-injected cells stained with anti-phospho-STAT6 (STAT6-P) antibody (green, asterisks). Scale bar = 25 μm. Image representative of three biologically independent experiments. **e** Phospho-STAT6 assays assessing the ability of the parasite to secrete the rhoptry protein ROP16 into the host cell that phosphorylates host STAT6 in the nucleus. Data are presented as box and whiskers plot (median with min to max, *n* = 3 biologically independent experiments). Statistical significance was assessed by a two-way ANOVA significance with Tukey’s multiple comparison. **f** Representative IFA of the e-vacuole assay to assess rhoptry secretion when invasion is blocked by cytochalasin D. A parasite (p) secreting e-vacuoles ROP1^+^ (asterisks) is depicted. Parasite DNA is visualize using DAPI (blue). Scale bar = 5 µm. Image representative of three biologically independent experiments. **g** E-vacuole assays assessing the ability of the parasites to secrete the rhoptry protein ROP1 into the host cell (mean ± SD; *n* = 3 biologically independent experiments). Statistical significance was assessed by a one-way ANOVA significance with Tukey’s multiple comparison. **h** Microneme secretion of extracellular parasites stimulated with 2% ethanol to assess the release of MIC2 in culture supernatant. pMIC2 processed MIC2, ESA excreted–secreted antigens. GRA1 is used as a control for constitutive secretion from dense granules. Samples derived from the same experiment and gels were processed in parallel. Image representative of three biologically independent experiments. **i** Kinetic assay representing the cell index of HFF infected with different parasite strains. (Mean ± SD; *n* = 3 biologically independent experiments). **j** Invasion assay showing that RON13-KD and RON4-KO parasites are defective in invasion. *P* values were determined by a one-way ANOVA significance with Tukey’s multiple comparison (mean ± SD; *n* = 3 biologically independent experiments). Statistical significance was assessed by a one-way ANOVA significance with Tukey’s multiple comparison. **k** Virulence of different strains in mice. Surviving mice at 84 days post infection were challenged with RH parasites (gray shaded). (*n* = 5 biologically independent animals). *P* values were determined by a Mantel–Cox test. Source data are provided as a Source data file.

To assess the role of RON13 kinase in *T. gondii*, knockdown parasites (RON13-KD) were generated by replacing the endogenous promoter with an anhydrotetracycline (ATc) repressible promoter (Supplementary Fig. 6a). The promoter swapping resulted in RON13 gene knockdown even without ATc treatment (Supplementary Fig. 6b, c). RON13-KD parasites were severely impaired in their lytic cycle and in host cell invasion (Fig. 5a–c and Supplementary Fig. 6d), but not affected in intracellular replication or egress from host cells (Supplementary Fig. 6e, f). Despite numerous attempts, a complete deletion of the gene could not be obtained. The kinase activity is important for RON13 function as complementation with the dead kinase mutant (RON13-KD/ron13dk) failed to fully rescue the phenotype. In contrast to ASP3-iKD^14^, RON5-iKD^19^ or ARO-iKD^20^ parasites previously reported to be unable to secrete their rhoptries, rhoptry discharge into the host cell was unaffected in RON13-KD parasites, when assayed by empty vacuole formation (e-vacuoles)^9^ or by ROP16-dependent host cell STAT6 phosphorylation^21^ (Fig. 5d–g). Likewise, microneme secretion was unaffected in RON13-depleted parasites (Fig. 5h). These results indicate that the invasion defect of RON13-KD parasites is not explained by a defect in rhoptry discharge. Of note, infection with RON13-KD parasites resulted in a premature damage of the host cell monolayer, a phenomenon not observed with RON4-KO parasites that display otherwise a comparable invasion defect (Fig. 5i, j and Supplementary Fig. 6g). Remarkably, RON13-KD parasites lost virulence in mice (Fig. 5k) along with a failure to elicit a protective immune response (Supplementary Fig. 6h and Supplementary Table 2). Such inability to trigger immune protection has been previously reported in mutant parasites defective in secretion of rhoptry contents^20,22^. In contrast, RON13-KD/ron13dk parasites were avirulent, but still able to induce seroconversion and protection in the infected animals challenged with the lethal RH strain (Fig. 5k and Supplementary Fig. 6h). Taken together, the presence of RON13 appears to favor host cell survival and is necessary to mount a protective immune response.

### RON13 phosphorylates mainly RONs

**Fig. 6:**
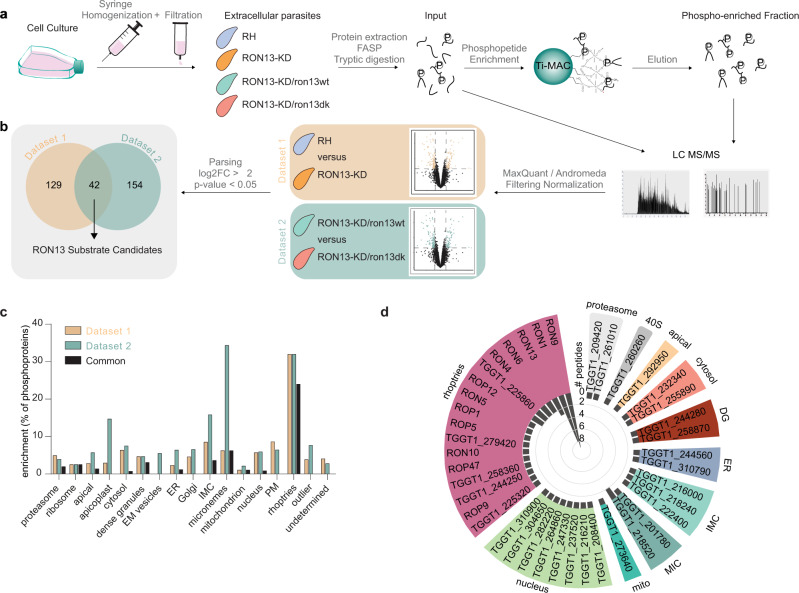
Rhoptry proteins are the major substrate of RON13. **a** Workflow of the shotgun approach and phosphoproteome analysis used to identify RON13 substrates. Samples were prepared for each strain from four independent experiments (*n* = 4 biologically independent experiments). RH (blue) RON13-KD (orange) RON13-KD/ron13wt (green) RON13-KD/ron13dk (salmon). **b** Venn diagrams of the phosphoproteins found in datasets 1 and 2. **c** Bar graph showing the percentage of phosphoproteins from datasets 1 and 2 relative to the total number of phosphoproteins, according to their predicted localization^23^. EM endomembrane, IMC inner membrane complex. **d** Polar plot of the number of phosphopeptides found in both datasets (common) binned by gene IDs and clustered, according to their predicted subcellular localizations^23^. Source data are provided as a Source data file.

RON13 substrate identification was undertaken using comparative phosphoproteome analyses of wt (RH) versus RON13-KD (dataset 1), as well as RON13-KD/ron13wt versus RON13-KD/ron13dk (dataset 2; Fig. 6a). A total of 14,681 phosphosites (mainly phosphoserines) in 3012 proteins were identified in the quadruplicate experiments. The overlap with a previous study^13^ is partial mainly due to differences in sample size, as well as in the experimental settings. The enrichment of phosphopeptides by immunoprecipitation done in this study allowed the detection of low abundance peptides leading to the identification of new phosphoproteins^13^ (Supplementary Data 3 and Supplementary Fig. 7a, b). Over 220 phosphopeptides corresponding to 171 phosphoproteins showed a >4-fold difference in abundance in wt compared to RON13-KD (dataset 1). Dataset 2 revealed 196 differentially phosphorylated proteins between the complemented strain RON13-KD/ron13wt and RON13-kD/ron13dk. Overall 42 proteins (63 phosphopeptides) are common to both datasets (Fig. 6b, Supplementary Fig. 7c, and Supplementary Data 4). We defined phosphopeptides as highly probable targets of RON13, when these were upregulated in both datasets, i.e., in wt versus RON-13KD, as well as RON13-KD/ron13wt versus RON13-KD/ron13dk. According to the spatial data obtained from hyper LOPIT^23^, most of the proteins hits are predicted to localize to the rhoptries (Fig. 6c, d and Supplementary Fig. 7d, e). Dataset 2 uncovered an increased phosphorylation of proteins from microneme, apicoplast and inner membrane complex likely attributable to the ~3-fold higher level of RON13 in RON13-KD/ron13wt parasites compared to wt (Supplementary Data 4). It is plausible that the overexpression of RON13 in the RON13-KD/ron13wt parasites led to mis-trafficking and thus to increased exposure of RON13 to targets outside of the rhoptries. A large proportion of the deduced RON13 substrates consists of previously annotated or predicted ROPs and RONs based on fractionation or transcriptomic data^23,24^ (Supplementary Data 5). Analysis of the phosphopeptide sequences led to the identification of a RON13 substrate consensus motif ExxxxxExxxNx**S**QSSxxxAxE (Supplementary Fig. 7f).

**Fig. 7:**
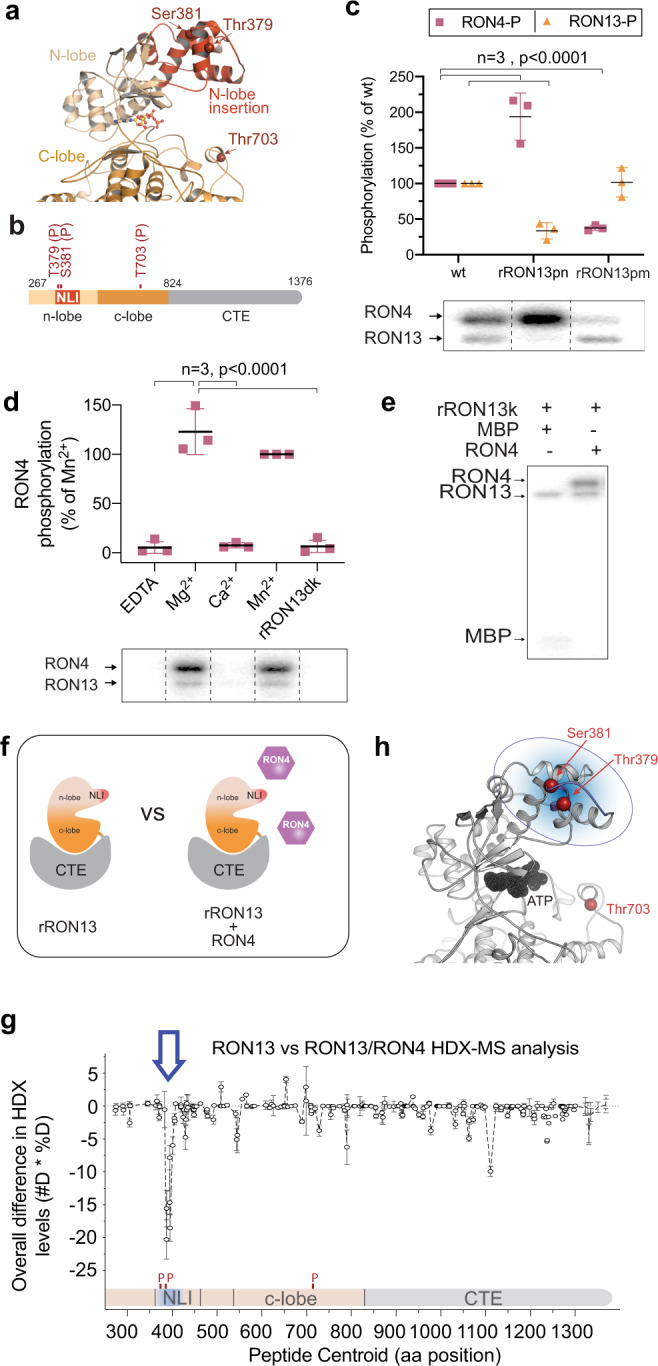
RON4 is a direct substrate of RON13. **a** Partial view of the RON13 structure highlighting the autophosphorylation sites (arrows, red). Kinase domain (orange tones). NLI (N-lobe insertion). **b** Schematic representation of RON13 structure organization highlighting the autophosphorylated residues (red) mutated in this study. The kinase domain and the C-terminal extension (CTE) are colored in orange and gray tones. **c** Radioactive kinase activity assays comparing RON4 phosphorylation and RON13 autophosphorylation for different rRON13 phospho-mutants; phosphonull (RON13pn) and phosphomimetic (RON13pm). Autoradiograph of a representative radioactive kinase assay is shown below the graph. Data shown as mean ± SD normalized to wt (*n* = 3 biologically independent experiments). *P* values were determined using a two-tailed paired *t* test. **d** Radioactive kinase activity assays characterizing the influence of ions on rRON4 phosphorylation by RON13. Densitometry data (mean ± SD; *n* = 3 biologically independent experiments) were normalized to RON4 phosphorylation in presence of Mn^2+^. Statistical significance was determined using a two-tailed paired *t* test. **e** Autoradiograph from a radioactive kinase activity assays in presence of MBP (left) or RON4 (right) as a substrate. **f** Schematic representation of the two states analyzed by HDX-MS. The kinase domain and the C-terminal extension (CTE) are colored in orange and gray tones. NLI (red). RON4 (violet). **g** Differences in HDX rates between rRON13dk alone and in the presence of RON4. A single region encompassing amino acids 377–390 is protected by RON4, indicating the contact site between the two proteins. Data shown as mean ± SD; *n* = 3 biologically independent experiments. **h** RON13 kinase domain highlighting the RON4-binding site (residues 377–390; blue) identified by HDX-MS. This region corresponds to the NLI and encompasses the two phosphosites Thr379 and Ser381. Source data are provided as a Source data file.

Of interest, three autophosphorylation sites were identified on RON13: Thr379 and Ser381 within the NLI in proximity to the ATP-binding site (N-lobe) and Thr703 closer to the activation loop (C-lobe; Fig. 7a, b). Mutation of these residues to alanine (phosphonull mutant, ron13pn) abolishes autophosphorylation in vitro, whereas mutation to aspartic acid (phosphomimetic mutant, ron13pm) does not impact RON13 autophosphorylation, suggesting that other residues than the ones identified are targeted (Fig. 7c). The complemented RON13-KD/ron13pm parasites showed a mild defect in invasion (Supplementary Fig. 8a–c), suggesting a modest contribution of RON13 autophosphorylation to its function. Taken together comparative phosphoproteomics reveals that RON13 phosphorylates rhoptry proteins in situ, including the components of the RON complex.

### RON4 is a substrate of RON13

RON4 which plays a critical role in MJ formation is differentially phosphorylated in both datasets. To validate it as a direct substrate, recombinant RON4 (rRON4) produced in insect cells was purified and its phosphorylation by rRON13 tested in vitro. Compared to the generic kinase substrate myelin basic protein (MBP) or autophosphorylation, rRON4 serves as a much better substrate, while maintaining the same ion preferences as observed with autophosphorylation (Fig. 7d, e), strongly suggesting that RON4 is an endogenous substrate. The phosphorylation status of rRON13 clearly influenced its RON4-directed phosphotransferase activity with the phosphonull mutant (rRON13pn) showing a twofold increased activity, whereas phosphomimetic significantly downregulating RON4 phosphorylation status (Fig. 7c). Thus, RON13 autophosphorylation directly influences substrate phosphorylation in vitro. Next, hydrogen/deuterium eXchange-mass spectrometry (HDX-MS)^25^ was applied to map the interaction sites between RON13 with RON4. HDX-MS can monitor protein dynamics based on the rate of exchange of protein amide protons with the solvent. Comparison of rRON13 dynamics alone and in presence of an excess of rRON4 (Fig. 7f) identified a single region protected from H/D exchange by RON4 (amino acids 377–390; Fig. 7g, Supplementary Fig. 9a, b, Supplementary Data 6, and Supplementary Table 3). As the only rRON13 region showing differential HDX rate upon addition of RON4 is small compared to the overall rRON13 structure, this suggests that the RON13–RON4 interaction is transient. Of relevance, this region is surface-exposed within the NLI and comprises two of the autophosphorylation sites, Thr379 and Ser381 (Fig. 7h). These results validate the direct interaction between rRON4 and rRON13, and identify the NLI as a substrate-recognition region.

### RON13-KD parasites are unable to assemble the MJ

**Fig. 8:**
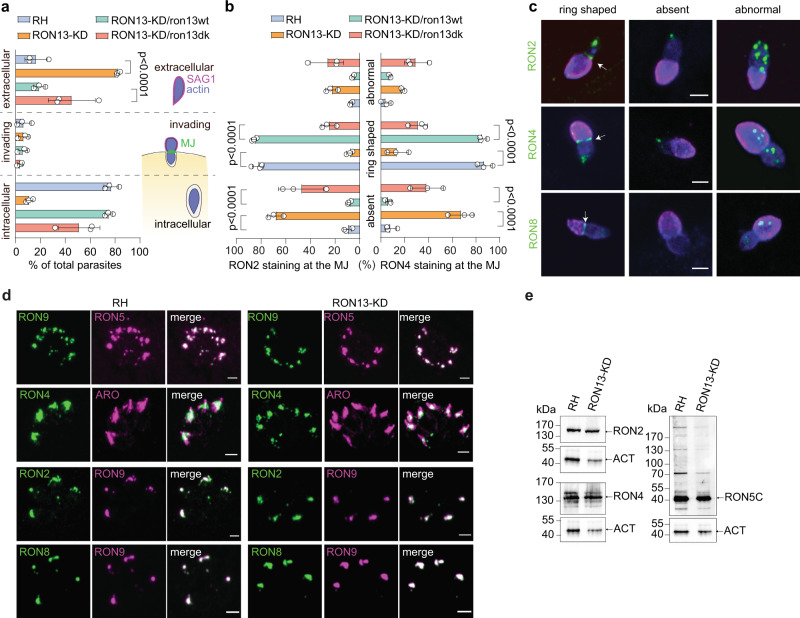
RON13 kinase activity is important for moving junction formation during invasion. **a** Graph representing the proportion of extracellular, invading, and intracellular parasites observed in the pulse-invasion assay. The scheme depicts the three stages of the invasion process considered in this assay (mean ± SD; *n* = 3 biologically independent experiments). Statistical significance was assessed by a one-way ANOVA significance with Tukey’s multiple comparison. **b** Quantification of the different types of RON2 and RON4 staining (absent, abnormal, and ring shaped) observed at the MJ of invading parasites. (Mean ± SD; *n* = 3 biologically independent experiments). Statistical significance was assessed by a one-way ANOVA significance with Tukey’s multiple comparison. **c** IFAs showing a representative example of RON2, RON4 and RON8 staining (green) of invading RON13-KD parasites obtained from three biologically independent experiments. An arrow indicates the bona fide ring-shaped MJ. Anti-SAG1 (magenta) and anti-actin (blue) antibodies stain the extracellular part of the parasite and the parasite cytoplasm, respectively. For the last row, anti-GAP45 antibodies (blue) were used. Scale bar = 2 µm. **d** IFAs on RH and RON13-KD intracellular parasites showing that proteins of the RON complex are well expressed and addressed to the rhoptry in RON13-KD-treated parasites. For the first row, RON5 is in magenta and RON9 in green. RON4, RON2, and RON8 are in green. Anti-ARO (magenta) and anti-RON9 (magenta) antibodies are used as rhoptry compartment markers. Scale bar = 2 µm. Image representative of three biologically independent experiments. **e** WB showing a comparable level of expression of RON complex proteins between RH and RON13-KD parasites using anti-RON2, anti-RON4, and anti-RON5C antibodies. Actin (anti-ACT) is used as a loading control. Image representative of three biologically independent experiments. Source data are provided as a Source data file.

**Fig. 9:**
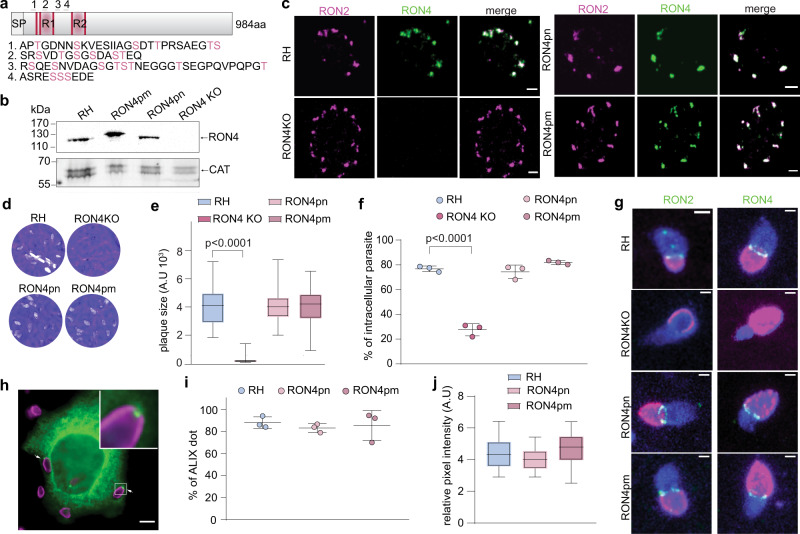
Phosphorylation of RON4 is not required for MJ formation and ALIX recruitment. **a** Schematic representation of RON4 protein. Two repeats (R1 and R2) and five motifs (pink lines) known to be important for binding host cytoskeleton are present in the N-term of RON4. The number (1, 2, 3, and 4) indicate the regions of RON4 known to be phosphorylated. The corresponding amino acid sequences and phosphorylated residues are indicated below in pink. These amino acids have been mutated in alanine or negatively charged amino acid to generate RON4 phosphonull (ron4pn) and RON4 phosphomimetic (ron4pm) mutant parasites, respectively. **b** Western blot showing that RON4pn and RON4pm are properly expressed. RON4-KO parasites have undetectable amount of RON4. Image representative of three biologically independent experiments. **c** IFA showing that mutation of phosphorylated residues either in Ala (ron4pn) or in Asp (ron4pm) does not impact RON4 (green) targeting to the rhoptry neck. Scale bar = 2 µm. Image representative of three biologically independent experiments. RON2 (magenta). **d** Plaque assay comparing the ability parasites to accomplish the lytic cycle. Image representative of three independent experiments. **e** Quantification of the plaque assay experiment. Data are presented as box and whiskers plot (median with min to max, *n* = 3 biologically independent experiments). Statistical significance was assessed by a one-way ANOVA significance with Tukey’s multiple comparison. **f** Invasion test showing the percentage of intracellular parasites reflecting their ability to invade. One-way ANOVA followed by Tukey’s multiple comparison was used to test differences between groups. (Mean ± SD; *n* = 3 biologically independent experiments). **g** IFA of invading parasites with RON2 (green) and RON4 (green) seen at the MJ except for RON4-KO invading parasites. SAG1 (magenta) and GAP45 (blue) were used to discriminate invading parasites. Scale bar = 1 µm. Image representative of three biologically independent experiments. **h** ALIX-GFP (green) expressing HeLa cells infected by RH parasites (magenta). ALIX recruitment at the MJ closure is indicated by white arrowhead. The inset displays the white boxed area at higher magnification. Scale bar = 5 µm. Image representative of three biologically independent experiments. **i** Quantification of the proportion of intracellular parasites associated with an ALIX dot in an in/out assay. Data are presented as box and whiskers plot (median with min to max, *n* = 3 biologically independent experiments). Statistical significance was assessed by a one-way ANOVA significance with Tukey’s multiple comparison. **j** Relative pixel intensity of ALIX dot recruited at the MJ in infected ALIX-GFP HeLa cells. When not stated two-way ANOVA followed by Tukey’s multiple comparison was used to test differences between groups. (Mean ± SD; *n* = 3 biologically independent experiments). Source data are provided as a Source data file.

As previously stated, the majority of RON13-KD parasites were defective in host cell entry and remained extracellular (~90%; Fig. 8a). However, among the minor subpopulation of invading parasites, the typical ring-shaped MJ was observed in <14% of the events (Fig. 8b). For most of the RON13-KD- and RON13-KD/ron13dk-invading parasites, the MJ components RON2/4/8 were absent or abnormally distributed at the host–parasite interface (Fig. 8b, c). Importantly, prior to secretion, the RON complex is properly processed, assembled, and accurately targeted to the neck of the rhoptries (Fig. 8d). Furthermore, co-immunoprecipitation experiments of RON4 confirmed that the intact RON complex is still able to associate to the microneme protein AMA1 (Fig. 8e and Supplementary Table 4). Consequently, RON13-catalyzed phosphorylation is a prerequisite for the stabilization of the MJ at the parasite/host interface without destabilizing the RON complex prior secretion. Of note, autophosphorylation of RON13 does not influences the correct assembly of the RON complex at the MJ post-secretion (Supplementary Fig. 10a, b). Identified as an endogenous RON13 substrate, modification of RON4 phosphorylation status alone is not sufficient to prevent MJ formation. Indeed, RON4 phosphonull (RON4pn) and RON4 phosphomimetic (RON4pm) mutant parasites (Fig. 9a–c) showed an unaltered lytic cycle and host cell invasion (Fig. 9d–f). In accordance with these observations, in both RON4 phosphomutant parasites, the stabilization of the RON complex at the MJ is unaffected and even if dispensable, we observed the recruitment of the host adaptor protein ALIX to the MJ^5^ (Fig. 9g–j). Overall, these results suggest a pleiotropic role of RON13 in the phosphorylation of each of the components of RON complex that together contribute to the success of host cell invasion.

## Discussion

RON13 is an atypical kinase possessing a unique NLI within the kinase domain that participates in substrate recognition. It harbors a large CTE critical for proper folding that is conserved among the coccidians. Comparisons of RON13 CTE structure to other proteins in the protein data bank did not reveal any clear match (only superficial similarity of the CTE fold to that of cytochrome P450, OleP (PDB ID: 5mnv), could be detected using PDBeFold^26^ (https://www.ebi.ac.uk/msd-srv/ssm/ssmcite.html)). Based on this, the CTE region of RON13 bares little to no structural similarity to any other proteins of known structure. RON13 clusters with the ROPK family members but differs from the ROPKs characterized to date. It localizes to the neck instead of the bulb of the rhoptries and does not appear to be secreted, although we cannot formally exclude it. Moreover, its kinase activity is essential for parasite survival and with its insensitivity to the broad kinase inhibitor staurosporine, it offers great promises for the identification of highly selective inhibitors. This atypical kinase operates in the secretory pathway and phosphorylates predominantly RON proteins, but also other ROPs known to be involved in subversion of host cell function.

**Fig. 10:**
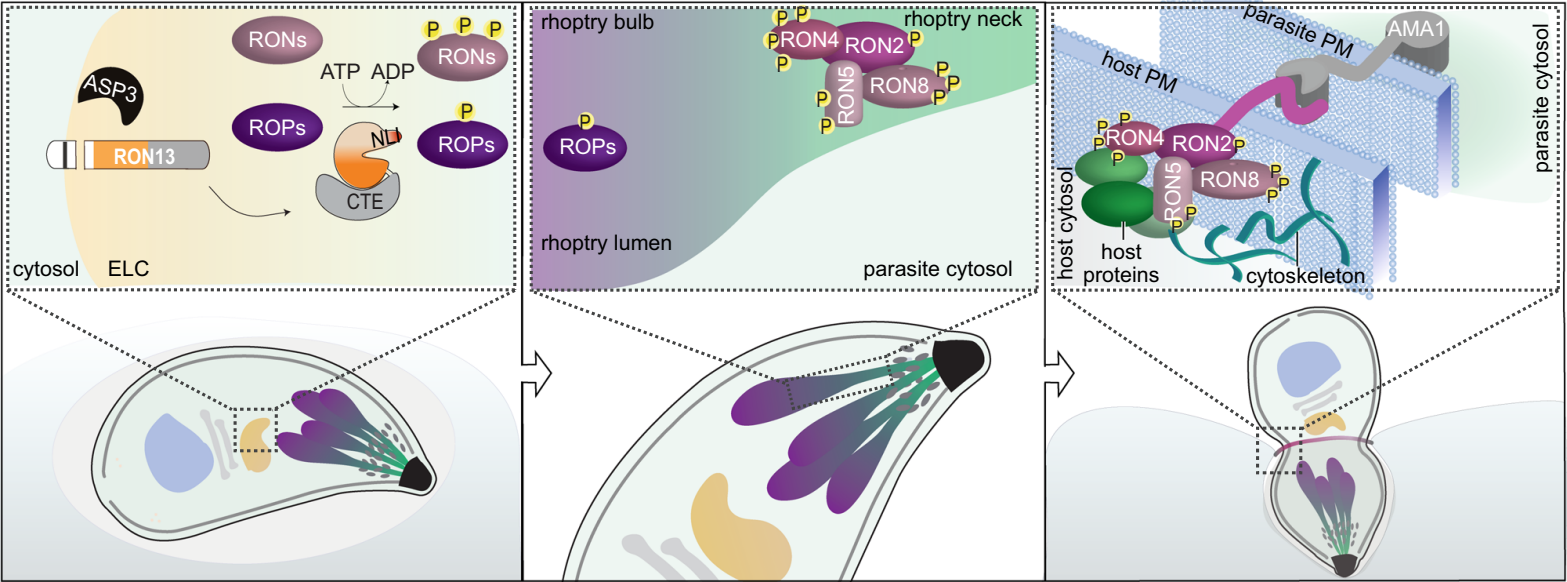
Cartoon summarizing the main findings. Left: in the endosome-like compartment (ELC) where protein trafficking to secretory organelles is determined, Asp3 protease cleaves the N-terminal transmembrane segment of RON13. Rhoptry proteins, including RONs and ROPs (violet tones), are phosphorylated by RON13 within this compartment. Center: RON13-phosphorylated proteins assemble to form the RON complex within the rhoptry neck, prior to being secreted into the host. Right: the RON complex, localized to the cytosolic face of the host, contributes to parasite invasion by forming a moving junction by associating with adhesins at the parasite plasma membrane (blue). Phosphorylated proteins of the RON complex additionally recruit host proteins (green) to assist in parasite invasion and subvert host cellular functions.

The premature destruction of the HFF monolayer observed under RON13-KD infection supports the hypothesis that this kinase modulates the function of an unidentified secreted ROP that controls host cell survival by an unknown mechanism. Furthermore, infection of mice with RON13-KD parasites failed to mount a resistance to reinfection. This phenomenon was previously observed only in parasites incompetent in invasion, resulting from defect in rhoptry discharge. Thus, RON13-dependent phosphorylation of effectors likely participates in modulation of host immune response. In contrast the impact of RON13 on the RON complex is more evident. The phosphorylation of the individual components of the RON complex is clearly dependent on RON13 and ensures proper MJ formation at the parasite–host interface and host invasion (Fig. 10). Mutations in the known phosphorylated sites of RON4 were not sufficient to recapitulate the phenotype observed in absence of RON13. This result suggests a cooperative role of phorsporylation of RON2/4/5 and 8 for the proper assembly of the MJ at the host PM, as observed for the interaction between RON complex and host proteins^5^. This study unravels a new level of complexity in the mechanism that governs invasion highlighting the phosphorylation as a key posttranslational modification that arms the secreted virulence factors necessary for host cell invasion. RON13 is found and conserved in the coccidian subgroup of the Apicomplexa. Importantly, the components of the RON complex are also phosphorylated in the deadliest malaria parasite *Plasmodium falciparum*. In silico analysis identified a predicted atypical kinase (PF3D7_3121100) expressed in the invasive stages of *P. falciparum* that also displays some homology to *Tg*RON13, including a large CTE and a determinant to enter the secretory pathway (Supplementary Data 7). Such an atypical kinase is worthy of deeper investigations as it might unravel a potential candidate for targeting invasion in malaria parasites via chemotherapeutic intervention.

## Methods

### DNA vector constructs and transfections

The sequence of the oligonucleotide primers and the plasmids generated are listed in Supplementary Table 5. The *T. gondii ΔKU80* RH^27^ and TaTi/*ΔKU80* (ref. ^28^) strains were used to obtain all the transgenic strains generated in this study (Supplementary Table 5). *Escherichia coli* XL-10 Gold chemically competent bacteria were used for DNA amplification.

To generate RON13-3Ty transgenic parasites, 1763bp corresponding to the C-terminal part of RON13 (TGGT1_321650) were amplified using the primers 6765/6766, and cloned into the ApaI and SbfI sites of the vector Ct‐ASP5‐3Ty‐DHFR^29^. A total of 40 µg of Ct-RON13-3Ty-DHFR plasmid were linearized by BstBI prior transfection.

For the YFP C-term tagging of ARO and RON11, we amplified the 3′ coding sequence of each gene using the primer pairs 8798/8799 (1043 bp) and 8800/8801 (1345 bp), respectively. PCR fragments were cloned into the LIC-YFP-DHFR vector^27^ and the final vectors were digested with BclI for ARO-YFP-DHFR and with AflII for RON11-YFP-DHFR, respectively, prior to transfection.

For YFP C-term tagging of RON13, we amplified the 3′ coding sequence using two pairs of primers 8802/8803 and 8804/8805 (1135 bp) to introduce a silent mutation, and thus generate an EcoRV restriction site used for LIC-RON13-YFP-DHFR vector linearization.

To generate RON13-KD parasite line, we amplified a PCR fragment encoding the TaTi trans-activator, the HXGPRT selection cassette and the TetO7S1 promoter from the vector 5′COR-pT8TATi1-HXtetO7S1myc^30^, using the primers 7013/7015 and KOD polymerase (Novagen, Merck). Primers 7013 and 7015 each share 30 and 32 bp of homology with the 5′ end of RON13. A specific gRNA vector generated by Q5 Hot Start site-directed mutagenesis kit (NEB) with the primers 4883/7012 on the vector template pSAG1::CAS9-GFPU6::sgUPRT^31^ was used to direct the insertion of the PCR cassette by double homologous recombination at the 5′ of the *ron13* locus.

To generate RON4-KO parasites, a gRNA targeting the 5′ coding sequence of RON4 was used to introduce a frameshift, resulting in RON4-KO. RON4 protein ablation was verified by IFA and western blotting using anti-RON4 antibodies (Supplementary Table 6).

RON4pm and RON4pn parasites were generated by modifying the *ron4* endogenous locus. Synthetic fragments corresponding to the bp 208 to bp 1104 of the RON4 cDNA flanked by 100 bp of ron4 gDNA homology region in 5′ and 3′ and EcoRV restriction sites were purchased from Genewiz®. In those fragments, the 23 phosphosites identified previously^13^ and in this study were mutated either to alanine (ron4pn) or to acid aspartic (ron4pm). Following enzymatic restriction, each fragment was co-transfected with the vector 2gRNA CRISPR-Cas9 plasmid^32^ containing 2gRNA targeting sequences located at 958 and 2519 bp from the start codon (Supplementary Table 5). Parasite clones were selected by FACS and screened by PCR for integration. The modified region was verified by sequencing.

To complement the RON13-KD line with a wild-type copy of *ron13* under a rhoptry promoter (p*ron5*), total RNA was extracted from RH tachyzoites using the RNeasy Minikit (Qiagen), and reverse transcribed in cDNA using the Superscript II kit (Invitrogen). The specific full-length cDNA of *ron13* gene was PCR-amplified in three parts using the primers 8178/7813 (part 1—2097 bp), 7814/7815 (part 2—960 bp), 7816/8179 (part 3—1152 bp), and the Pangea polymerase (Canvax).The three fragments were Gibson assembled with the vector pUPRT-promRON5-G13-4myc^5^ digested by PacI/EcoRV. A total of 60 µg of the resulting vector pUPRT-pRON5-RON13-4myc was digested by KpnI/BamHI and co-transfected with 15 µg of the pU6-Cas9-Universal-gRNAUPRT vector^33^. Parasites for which the integration of the vector pUPRT-p*ron*5-RON13-4myc at the UPRT locus by double homologous recombination had occurred were selected by 5′-fluo-2′-deoxyuridine (FUDR) negative selection and designated as RON13-KD/ron13wt. Point mutagenesis introduced by Q5 site-directed mutagenesis (NEB) on p*ron*5-RON13-4myc were used to generate ron13dk, ron13cte, ron13pn, and ron13pm vectors (Supplementary Table 5).

To assess RON13 secretion into the host cell, we modified the vector SP3-toxofilin-BLA (kind gift from Dr. Lodoen MB.)^16^ by cloning RON13 cDNA in frame with the beta-lactamase tag. The fragment p*ron*5-RON13-4myc (5565 bp) was PCR-amplified with the pair of primers 8263/8264 using the FastPangea polymerase (Canvax), and subconed in the SP3-toxofilin-BLA vector previously digested with SfoI and HindIII. The resulting construct was sequence verified and transfected in RH parasites. Transgenic parasites were selected by addition of hypoxanthine and mycophenolic acid.

### Parasite culture

Tachyzoites from parental and modified strains were propagated in confluent human foreskin fibroblasts (HFFs) with Dulbecco modified Eagle’s medium supplemented with 5% fetal bovine serum, 2 mM glutamine, and 25 µg/mL gentamicin. To generate transgenic parasites, 20 μg of linearized plasmid or of PCR product were transfected with a mix of 15 µg Cas9‐sgRNA plasmid. Transgenic parasites were selected by addition of mycophenolic acid (25 µg/mL) and xanthine (50 µg/mL) exploiting the HXGPRT selection cassette, 1 µM of pyrimethamine for the DHFR selection cassette, and 5 µM of FUDR for the negative selection at the UPRT locus. Clones were isolated by limiting dilution or FACS and checked for proper integration by immunofluorescence (Supplementary Table 6), western blotting (Supplementary Table 6), and PCR on genomic DNA (Supplementary Table 5) using the GoTaq polymerase (Promega). ATc treatment were done for 48 h at 1 µg/mL. For treatment with 49c, infection was performed in the presence of 1 µM of 49c and parasites were collected 48 h post infection.

### Western blotting

Freshly egressed tachyzoites were pelleted by centrifugation, washed with PBS, and resuspended in SDS–PAGE buffer (50 mM Tris-HCl, pH 6.8, 10% glycerol, 2 mM EDTA, 2% SDS, 0.05% bromophenol blue, and 100 mM dithiothreitol (DTT)). After boiling and sonication, samples were subjected to SDS–PAGE under reducing conditions. Proteins were transferred to nitrocellulose membrane and immunoblot analysis was performed. Primary antibodies (Supplementary Table 6) were diluted in 5% milk/0.05% Tween-20/PBS. Secondary antibodies coupled with HRP were purchased from Invitrogen® and were used according to manufacturer information. All western blots were done in triplicate. Uncropped and unprocessed western blot can be found in the Source data file of this manuscript.

### Immunoprecipitation of RON4 and analysis by mass spectrometry

Freshly egressed parasites RH and RON13-KD from a 10 cm dish were centrifuged, rinsed in PBS, and lysed in PBS-1%Triton X-100 in the presence of protease and phosphatase inhibitors (PhosSTOP™, Roche®, and A32965, ThermoFisher®). Following five cycles of freeze/thaw, the lysate was sonicated, incubated 10 min on ice, and the soluble fraction was incubated with 10× concentrated supernatant containing RON4 mAb 4H1 overnight. The next day protein A-sepharose equilibrated in IP buffer was added and the incubation was continued for 1–2 h at 4 °C. Beads were then washed three times in IP buffer, two times in PBS, resuspended in SDS–PAGE sample buffer, and boiled. Sample were then loaded on a polyacrylamide gel and directly upon entry in the separating gel, migration was stopped and bands were cut, and submitted to the Proteomic Core Facility, Faculty of Medicine, Geneva.

To prepare the samples, bands of interest were digested as follow: gel pieces were destained by incubation in 100 μL of 50% acetonitrile (AcN) in 50 mM ammonium bicarbonate (AB) for 15 min at room temperature. Proteins were reduced by incubation of gel pieces for 30 min at 50 °C in 100 μL of 10 mM DTT in 50 mM AB. DTT solution was then replaced by 100 μL of 55 mM iodoacetamide in 50 mM AB, and protein were alkylated by incubation of the gel pieces for 30 min at 37 °C in the dark. Gel pieces were then washed for 15 min with 100 μL of 50 mM AB and for 15 min with 100 μL of 100% AcN. Gel pieces were then air dried for 15 min at room temperature. Dried pieces of gel were rehydrated for 45 min at 4 °C in 35 μL of a solution of 50 mM AB containing trypsin at 10 ng/μL and 0.01% of Protease Max Surfactant trypsin enhancer (Promega). Subsequently, 10 μL of 0.01% of Protease Max in 50 mM AB was added before incubating the samples for 1 h at 50 °C. Supernatant was transferred to a new polypropylene tube and an additional peptide extraction was performed with 70 μL of 20% FA for 15 min at room temperature with occasional shaking. Extractions were pooled, completely dried under speed vacuum, and stored at −20 °C.

For ESI-LC-MSMS, samples were diluted in 15 μL of loading buffer (5% CH_3_CN and 0.1% FA) and 2 μL were injected on column. LC-ESI-MS/MS was performed on a Q-Exactive Plus Hybrid Quadrupole-Orbitrap Mass Spectrometer (Thermo Fisher Scientific) equipped with an Easy nLC 1000 liquid chromatography system (Thermo Fisher Scientific). Peptides were trapped on a Acclaim pepmap100, C18, 3 μm, 75 μm × 20 mm nano trap column (Thermo Fisher Scientific) and separated on a 75 μm × 250 mm, C18, 2 μm, 100 Å Easy-Spray column (Thermo Fisher Scientific). The analytical separation was run for 90 min using a gradient of H_2_O/0.1% formic acid (solvent A) and CH_3_CN/0.1% formic acid (solvent B). The gradient was run as follows: 0–5 min 95% A and 5% B, then to 65% A and 35% B for 60 min, then to 10% A and 90% B for 10 min, and finally stay 15 min at 10% A and 90% B. Flow rate was of 250 nL/min for a total run time of 90 min. For MS survey scans, the resolution was set to 70,000 and the ion population was set to 3 × 106 with an *m*/*z* window from 400 to 2000. For data-dependant analysis, up to 15 precursor ions were isolated and fragmented by higher-energy collisional dissociation HCD at 27% normalized collision energy (NCE). For MS/MS detection, the resolution was set to 17,500, the ion population was set to 1 × 105 with an isolation width of 1.6 *m*/*z* units. Peak lists (MGF file format) were generated from raw data using the MSConvert conversion tool from ProteoWizard. The peaklist files were searched against the ToxoDB_Tgondii_GT1 database (http://ToxoDB.org, release 42, 8460 entries) combined with an in-house database of common contaminant using Mascot (Matrix Science, London, UK; version 2.5.1). Trypsin was selected as the enzyme, with one potential missed cleavage. Precursor ion tolerance was set to 10 p.p.m. and fragment ion tolerance to 0.02 Da. Carbamidomethyl of cysteine was specified as fixed modification. Deamidation of asparagine and glutamine, and oxidation of methionine were specified as variable modifications. The Mascot search was validated using Scaffold 4.9.0 (Proteome Software). Peptide identifications were accepted if they could be established at >5.0% probability to achieve an false discovery rate (FDR) < 0.1% by the Peptide Prophet algorithm^34^ with Scaffold delta-mass correction. Protein identifications were accepted if they could be established at >26.0% probability to achieve an FDR < 1.0 % and contained at least two identified peptides. Protein probabilities were assigned by the Protein Prophet algorithm^35^. Proteins that contained similar peptides and could not be differentiated based on MS/MS analysis alone were grouped to satisfy the principles of parsimony. The mass spectrometry proteomics data have been deposited to the ProteomeXchange Consortium via the PRIDE^36^ partner repository with the dataset identifier PXD021516.

### Plaque assay

Confluent HFF monolayers were inoculated with freshly egressed parasites in absence or presence of ATc. Seven days post infection, the infected cells were fixed with 4% paraformaldehyde (PFA) and stained with a crystal violet (Sigma). Lysis plaque area were quantified using ImageJ software (NIH, version 1.53c). Ten lysis plaque were quantified for each strain in each replicate. Quantification represents the mean (±SD) from three independent experiments. Statistical significance was assessed by one-way ANOVA significance test with Tukey’s multiple comparison on GraphPad Prism 8 software.

### Intracellular growth assay

Freshly egressed parasites pretreated ±ATc for 24 h were used to infect new confluent HFF on glass coverslips. Cells were fixed 24 h post infection and parasite were visualized using anti-GAP45 antibodies by IFA. For each condition, at least 200 vacuoles were counted. Experiments were performed in three independent replicates. Results are presented as mean ± SD. Two-way ANOVA followed by Tukey’s multiple comparison was used to test differences between groups on GraphPad Prism 8 software.

### Egress assay

Freshly egressed parasites pretreated ±ATc for 12 h were used to infect new confluent HFF on glass coverslips. Cells were fixed 30 h post infection and parasite boundaries were visualized using anti-GAP45 antibodies by IFA. At least 200 vacuoles were counted. Results are presented as mean ± SD and the experiment performed in three independent replicates. Two-way ANOVA followed by Tukey’s multiple comparison was used to test differences between groups on GraphPad Prism 8 software.

### Microneme secretion

Freshly egressed RH and RON13-KD parasites were resuspend in harvested and resuspended in intracellular buffer (5 mM NaCl, 142 mM KCl, 1 mM MgCl_2_, 2 mM EGTA, 5.6 mM glucose, and 25 mM HEPES, pH 7.5) ± ethanol 2%. After an incubation for 30 min at 37 °C, parasites were pelleted and washed once with PBS, while the supernatant was cleared again by a second centrifugation. Pellet and supernatant (excreted–secreted antigens—ESA) were resuspend in sample buffer before boiling and immunoblotting. MIC2 secretion and processing was assessed by western blotting. GRA1 is used as a marker for constitutive secretion and catalase as a positive control of parasite lysis. This experiment as done in triplicate.

### Rhoptry secretion by e-vacuole assay

Freshly egressed parasites were washed once with PBS, resuspended in prechilled egress buffer containing 1 µM cytochalasin D, and incubated 10 min on ice. Then, the parasites were added to prechilled HFFs. After a short centrifugation and 20 min on ice to allow the parasites to settle, HFFs were washed with cold PBS. The PBS was replaced by warm media + cytochalasin D. Rhoptry secretion was triggered by incubating the infected coverslips at 37 °C for 20 min. The coverslips were then fixed with PFA 4%. Approximately 100 parasites were counted in duplicate in three independent experiments. Results are presented as mean ± SD and the statistical analysis was done using one-way ANOVA statistical test on GraphPad Prism 8 software.

### Rhoptry secretion by phospho-STAT6

Freshly egressed parasites (5 × 10^5^) pretreated ±ATc for 48 h were used to infect HFF-coated coverslips. Following a short centrifugation (30 s at 1100 × *g*) and an incubation on ice for 20 min, the cells were incubated at 37 °C for 20 min and fixed with ice-cold methanol. After blocking, immune-detection was performed using anti-phospho-STAT6 antibody (Cell signaling 9361; 1/400). The experiments were done in triplicate and >200 host cell nuclei were counted each time. *P* values were calculated using a two-way ANOVA test on GraphPad Prism 8 software. The line represents the median and the whiskers represent the min to max values from 30 fields.

### Red/green invasion assay

Freshly egressed parasites were allowed to invade a HFF for 30 min before fixing with PAF/Glu for 7 min. A first immune-detection using anti-SAG1 antibodies on non-permeabilized cells was performed. Cells were then fixed with 1% formaldehyde/PBS for 7 min, washed with PBS, and permeabilized using 0.2% Triton X-100/PBS. Parasites were labeled using anti-GAP45 antibodies. At least 100 parasites were counted in duplicate for each strain in three independent experiments. Results are presented as mean ± SD, and the statistical analysis was done using one-way ANOVA followed by Tukey’s multiple comparisons on GraphPad Prism 8 software.

### In/out assay and moving junction formation

Freshly egressed parasites were resuspended in cold DMEM and added to prechilled HFF monolayer on a glass coverslip. Following a quick centrifugation (30 s at 500 × *g*) and an incubation of 20 min on ice, the culture was incubated at 38 °C for 3 min in a water bath and fixed with PFA for 15 min. A first indirect immuno-detection using anti-SAG1 antibodies, and secondary antibodies was done followed by another fixation step with 1% formaldehyde/PBS for 7 min and permeabilization with 0.025% saponin for 10 min. After blocking, a second immuno-detection with anti-RON2, or anti-RON4 or anti-RON8 antibodies was performed. Finally, cells were permeabilized with 0.2%Triton X-100/PBS and stained with anti-GAP45 or anti-actin antibodies. Extracellular parasites (SAG1+/GAP45+), in/out (half SAG1+/GAP45+; half GAP45+), and intracellular parasites (GAP45+) were quantified. RON2 and RON4 staining at the MJ was examined for at least 100 parasites. Values represent means ± SD, from three independent assays. *P* values reported are from two-way ANOVA statistical analysis followed by Tukey’s multiple comparison on GraphPad Prism 8 software.

### ALIX recruitment

The recruitment of ALIX at the MJ was assessed by quantifying ALIX-positive staining at the site of invasion^5^. HeLa cells (0.8 × 10^5^) were seeded on glass coverslips and transfected after 24 h with 500 ng of ALIX-GFP plasmid (kind gift from Dr. M. Lebrun) using lipofectamine 2000® (ThermoFisher), according to the manufacturer’s protocol. The following day, in/out assays were performed and cells were fixed with methanol and processed for IFA using anti-GFP and anti-GAP45 antibodies. ALIX recruitment was assessed over 50 invading parasites. Results are presented as mean ± SD of three independent replicates. *P* values reported are from two-way ANOVA statistical analysis followed by Tukey’s multiple comparison. Pixel intensity at the site of ALIX recruitment was normalized to the background fluorescence in the HeLa cytoplasm. The pixel intensity of thirty ALIX dots were quantified per experiment and results are presented as mean ± SD of three independent replicates. *P* values reported are from two-way ANOVA statistical analysis followed by Tukey’s multiple comparison on GraphPad Prism 8 software.

### Topology

To assess RON13 topology, we used GFP nanobodies (kind gift from Clare Harding)^37^. Briefly, 2 × 10^6^ RH (negative control), ARO-YFP, and RON11-YFP (positive controls) and RON13-YFP parasites were transiently transfected with 30 µg of mCherry-GFP nanobodies-DD plasmid and used to infect an HFF monolayer. The day after, 1 µM of Shield was added to the infected cells for 1 h prior to methanol fixation and immunofluorescence assay using anti-myc and anti-GFP antibodies. This experiment was done in triplicate.

### Solubility test

To assess the solubility of RON13, extracellular parasites ASP3-iKD/RON13-3Ty from culture pretreated ±ATc for 48 h, were pelleted and resuspended in PBS or PBS-2% TX-100. Samples were lysed by freeze-thawing and incubated at 37 °C. The pellet and the soluble fraction were separated by centrifugation for 30 min at 4 °C max and 15,000 × *g*. Samples were finally resuspended with SDS–PAGE loading buffer (±10 mM DTT) and heated at 95 °C for 10 min prior to separation. This experiment was done in triplicate.

### Beta-lactamase assay

The activity of the beta-lactamase fusion proteins was assessed on extracellular parasites. Extracellular parasites from RH, toxofilin-BLA, and RON13-BLA strains were washed twice in PBS and incubated with the BLA substrate CCF4-AM (Thermo Fisher Scientific) or DMSO (control) in DMEM, 5% FCS for 2 h in the dark at room temperature, according to manufacturer’s protocol. Following two washes in PBS, extracellular parasites were distributed in a 96-well plate and the fluorescence at 450 and 550 nm (excitation 405 nm) was read on a SpectraMax Paradigm reader (Molecular Devices,LLC) at the READS platform of the University of Geneva. The experiment was performed in three independent experiments. The relative fluorescence was obtained by subtracting the background fluorescence (DMSO wells) to the absolute fluorescence (CCF4-AM wells).

Analysis of rhoptry protein secretion into the host cell was assessed by flow cytometry by detection of beta-lactamase activity in the host cell^16^. HFF monolayers were infected with extracellular parasites from RH, toxofilin-BLA, and RON13-BLA parasites at a MOI of 30. After 1 h, cells were washed and incubated with the BLA substrate CCF4-AM (Thermo Fisher Scientific) or DMSO (control) for 2 h in the dark at room temperature. Cells were washed three times with PBS, trypsinised and analyzed by flow cytometry on a Gallios flow cytometer (Beckman Coulter) at the flow cytometry platform (University of Geneva, Switzerland). Samples were excited at 405 nm and coumarin and fluorescein were detected with the 450/50 nm laser and the 550/40 nm laser, respectively. FlowJo (Becton, Dickinson & Company) and Kaluza (Beckman Coulter) softwares were used for analysis. Graphs were made using GaphPad Prism 8 and represent means ± SD from three independent assays. Statistical significance was assessed by a paired *t* test.

### Kinetic assay measuring host cell impedance

Host cell damage following infection was assessed by the Live Cell Analysis System xCELLigence (OLS®) that measure cellular impedance in a kinetic assay and collected on the RTCA software (Agilent, version 1.0). HFF (5 × 10^4^ cells) were seeded in an eight-well E-plate and cellular impedance was measured every 30 min during 24 h. The next day, medium was renewed 2 h prior infection. HFF were infected with 5 × 10^5^ freshly egressed parasites, the plate was centrifuged 1 min at 2500 × *g* and put back in the xCELLigence. Extracellular parasites were washed away 30 min post infection and cellular impedance was measured every 30 min during 60 h. Data were normalized according to the cellular impedance at the time of the last wash, and expressed as a percentage of the impedance measured for uninfected wells overtime. Three independent experiments were done in duplicate.

### Animal experimentation

Virulence assays were performed by intraperitoneal injection of 100 freshly lysed tachyzoites (RH and mutant strains) in 7-week-old female CD1 mice (Charles River). Mice were monitored daily and sacrificed at the onset of signs of acute infection (ruffled fur, difficulty moving, and isolation). Surviving mice were assessed for seroconversion by Western blotting, as well as challenged with an inoculation with 1000 freshly lysed RH tachyzoites at 84 days post infection. Surviving mice were humanely sacrificed at the end of the experiment, ~40 days after the challenge infection.

### Ethics statement

All animal experiments were conducted with the authorization numbers GE121-19, according to the guidelines and regulations issued by the Swiss Federal Veterinary Office. All animals were housed at the University of Geneva in room with day/night cycle of 12 h/12 h and constant ambient temperature of 22 °C and 35% humidity, respectively.

### Transmission electron microscopy (TEM)

Infected HFF cells grown on a round glass coverslips were fixed with 2.5% glutaraldehyde (Electron Microscopy Sciences) and 2% PFA (Electron Microscopy Sciences) in 0.1 M sodium cacodylate buffer at pH 7.4 for 1 h at room temperature. Traces of fixative were removed by extensive washing with 0.1 M sodium cacodylate buffer, pH 7.4, and postfixed with reduced 1% osmium tetroxide (Electron Microscopy Sciences) with 1.5% potassium ferrocyanide in 0.1 M sodium cacodylate buffer, pH 7.4 for 1 h and immediately followed by 1% osmium tetroxide alone (Electron Microscopy Sciences) in the same buffer for 1 h. After two washes in double-distilled water (ddH_2_0) for 5 min each wash samples were en bloc stained with aqueous 1% uranyl acetate (Electron Microscopy Sciences) for 1 h or overnight at 4 °C. After a 5 min wash in ddH_2_0, cells were dehydrated in graded ethanol series (2× 50, 70, 90, 95%, and 2× absolute ethanol) for 10 min each wash and infiltrated with graded series of Durcupan resin (Electron Microscopy Sciences) diluted with ethanol at 1:2, 1:1, and 2:1 for 30 min each. Next, cells were infiltrated twice with pure Durcupan for 30 min each and with fresh Durcupan resin for additional 2 h. Finally, coverslips with cells facing down, were placed on 1 mm thick teflon rings filled with resin and placed on glass slide coated with mold separating agent (Glorex) and polymerized in the oven at 65 °C for 24 h. The glass coverslip was removed from the cured resin disk by alternate immersion into hot (60 °C) water and liquid nitrogen, until the glass parted. Laser microdissection microscope (Leica Microsystems) was used to select suitable areas and to outline their positions on the resin surface to cut out from the disk using a single-edged razor blade and glued with superglue (Ted Pella) to a blank resin block. The cutting face was trimmed using a Leica Ultracut UCT microtome (Leica Microsystems) and a glass knife. A 70 nm ultrathin serial sections were cut with a diamond knife (DiATOME) and collected onto 2 mm single slot copper grids (Synaptec, Ted Pella) coated with Formvar plastic support film.

Sections were examined using a Tecnai 20 TEM (FEI) operating at an acceleration voltage of 80 kV and equipped with a side-mounted MegaView III CCD camera (Olympus Soft-Imaging Systems) controlled by iTEM acquisition software (Olympus Soft-Imaging Systems).

### FIB-SEM and 3D reconstruction

Sample for FIB-SEM imaging were prepared in the same way as for the TEM. Selected parasitophorous vacuoles were marked on the surface of the resin block by laser microdissection microscope (Leica Microsystems). Either whole resin block or large cut out area containing the region of interest was glued onto a flat SEM stub with superglue, and silver conductive paste was applied on each side of the resin block to ensure the conductivity within SEM. Finally, the mounted sample was gold coated with 20 nm thick layer of gold.

The samples were imaged inside a FEI Helios NanoLab G3 UC DualBeam microscope (FEI). Ion beam was used in conjunction with a gas injection system to deposit a thick (∼1.5 µm) layer of platinum on the top surface of the sample above the region of interest to reduce the FIB milling artefacts. The imaging surface was exposed by creating the front trench using 21 nA of focused ion beam current at 30 kV voltage and subsequently two side trenches were created using the same parameters. AutoSlice and View G3 software (FEI) was used to acquire the serial SEM images. Focused ion beam at current of 2.5 pA and 30 kV of acceleration voltage was applied to mill 10 nm layer from imaging face, and freshly exposed surface was imaged with back scattered electron beam at current of 400 pA and at acceleration voltage of 2 kV, the dwell time of 9 µs/pixel and at the resolution of 4 nm/pixel. Serial images were combined into single image stack, aligned, and scaled down to obtain imaged volume with isotropic pixel properties of the 10 nm/pixel in all *x*-, *y*-, and *z*-dimension using the FIJI program (fiji.sc/). Semiautomated approach using Ilastik software (ilastik.org) was used for segmentation and 3D reconstruction. Final 3D models were visualized using the Blender software (v.2.79; blender.org).

### Image acquisition

Confocal images were acquired with a confocal laser scanning microscope LSM700 (Zeiss) and confocal expansion microscopy images were collected with a TCS SP8 STED ×3 microscope (Leica) at the Bioimaging Core Facility of the University of Geneva Medicine Faculty. Image processing for expansion microscopy was performed using LasX Software (Leica, version 3.7.0), while ImageJ (NIH; version 1.53c) was used otherwise. The antibodies used for immunofluorescence assay and their dilutions are listed in Supplementary Table 6. Secondary antibodies were purchased from Invitrogen® and used according to the manufacturer’s protocol.

### Ultrastructure expansion microscopy

U-ExM was performed on extracellular parasites^38^. Extracellular parasites were resuspended in PBS and settled on a poly-d-lysine (Gibco) coated 12 mm coverslip. Protein crosslinking was performed by incubating the coverslip in 1.4% formaldehyde/2% acrylamide/PBS solution for 5 h at 37 °C. The monomer solution (19% sodium acrylate (Sigma)/10% acrylamide (Sigma)/0.1% N, N′-methylenbisacrylamide (Sigma)/PBS), as well as the 10% TEMED and 10% APS solutions were thawed on ice. The gelation step was performed on ice as well with the coverslip incubated face down on a drop of 35uL of the gelation solution (90 µL monomer solution + 5 µL TEMED 10% + 5 µL APS 10%) in a humid chamber for 5 min followed by an incubation at 37 °C for 1 h. The gel and coverslip were then transferred to six-well plate filled with denaturation buffer (200 mM SDS, 200 mM NaCl, and 50 mM Tris pH 9.0) face up for 15 min at room temperature under agitation. When the gel detached from the coverslip, it was transferred into a Eppendorf filled with denaturation buffer and incubated at 95 °C for 1.5 h. A first round of expansion was performed by incubating the gel three times in ddH_2_0 for 30 min. Prior incubation with the primary antibodies diluted in Tween 0.1%/PBS for 3 h at 37 °C, two rounds of gel shrinkage were performed by replacing the ddH_2_O with PBS for 15 min. Following three washes of 10 min each in Tween 0.1% / PBS, secondary antibody detection was performed. A last round of expansion was done by incubating the gel for 30 min twice in ddH_2_O and then overnight. For imaging, pieces of gel were put on poly-d-lysine (Gibco) coated 24 mm coverslip with sample facing down clipped on 35 mm round adapters (Okolab). Images were acquired on a Leica Thunder inverted microscope using 63× 1.4 NA oil objective with Small Volume Computational Clearing mode to obtained deconvolved images. 3D stacks were acquired. Images were analyzed and merged using LasX software (Leica).

The antibodies used and the appropriate dilution are mentioned in Supplementary Table 6.

### Phylogeny

Sequences of Apicomplexan kinases were procured from EuPathDB and aligned using MUSCLE sequence alignment software (version 3.8.31)^39^. The resulting sequence alignment was manually curated utilizing BioEdit (http://www.mbio.ncsu.edu/bioedit/bioedit.html) to edit out uninformative alignment positions. Phylogeny tree was generating utilizing PhyML (version 3.0)^40^ on the curated MUSCLE alignment. The curated alignment of sequences used in the phylogenetic analysis can be found in Supplementary Data 8.

### RON13 purification for antibody generation

The RON13 kinase domain (163–769) was inserted into a pGEX-modified vector to contain a N-terminal His10-tag followed by a tobacco etch virus (TEV) protease recognition site (Gibson; digested with NotI/KpnI). The plasmid was transformed into Rosetta DE3 pLysS cells and cultures were grown until reaching an OD600 of 0.6. Protein production was induced by adding IPTG to a final concentration of 0.5 mM and left to incubate for 4 h at 37 °C. Cells were then harvested by centrifugation at 4000 × *g* for 15 min and the pellet was frozen at −80 °C. The pellet from 1 L of cells was resuspended in 25 mL of bacteria lysis buffer (50 mM Tris pH 8, 400 mM NaCl, 15 mM imidazole, 0.05 % Triton X-100, 8 M urea, and 3 mM beta-mercaptoethnol (2-ME)). Cells were lysed by two passages through a French Pressure Cell at 1000 p.s.i. Cell membranes and debris were removed by centrifugation at 35,000 × *g* for 30 min at 4 °C. The supernatant containing soluble proteins was applied to a 5 mL His-trap FF column (GE healthcare), washed with 50 mL of lysis B buffer, followed by a second washing step using bacteria Ni-A buffer (15 mM Tris pH 8, 200 mM NaCl, 15 mM imidazole, 5 M urea, and 3 mM 2-ME). Protein was then eluted using bacteria Ni-A buffer supplemented with 430 mM imidazole. Eluted proteins were concentrated to 1 mg/mL using AMICON 30 MWCO concentrators and used as antigens for antibodies generation at the Geneva Antibody Facility (University of Geneva, Switzerland).

### ASP3 purification from insect cells

Recombinant ASP3 protease was expressed in baculovirus-infected Sf9 insect cells. Baculovirus were generated using a modified pFastBac vector encoding a N-terminal Melittin secretion signal, followed by ASP3 protease (TGME49_ 246550, amino acids 38–643) and a C-terminal His6 tag. Cells infected for 68 h were harvested by centrifugation at 4000 × *g* for 15 min at 4 °C. The pellet was dissolved in Asp3 lysis buffer (50 mM Tris-HCl pH 8, 500 mM NaCl, 10 mM imidazole, 0.1% Triton X-100, and 3 mM 2-ME) and lysed by one passage through a French Pressure Cell at 1500 p.s.i. Cell membranes and debris were removed by centrifugation of the lysate at 35,000 × *g* for 35 min at 4 °C. The supernatant containing soluble protein was applied to a 5 mL His-trap column (GE healthcare), washed with 40 mL of Asp3 lysis buffer and with 40 mL of Asp3 Ni-A buffer (20 mM Tris pH 8, 150 mM NaCl, 15 mM imidazole, and 3 mM 2-ME). Protein was eluted with 15 mL of Asp3 Ni-B buffer (20 mM Tris pH 8, 150 mM NaCl, 350 mM imidazole, and 3 mM 2-ME). Eluted protein was diluted fivefold with Asp3 Q-A buffer (20 mM Tris pH 8, 10 mM NaCl, and 3 mM 2-ME) and loaded to a 5 mL Q-HP column (GE healthcare). The protein was eluted by applying a gradient from 10 to 30% Asp3 Q-B buffer (20 mM Tris pH 8, 1 M NaCl, and 3 mM 2-ME) in 40 mL. Fractions containing ASP3 protein were pooled, concentrated to 1 mL using AMICON 30 MWCO concentrators, and loaded on a size-exclusion Superdex 200 10/300 column at 4 °C equilibrated in Asp3 final buffer (50 mM Hepes pH 7.4/150 mM KCl, 5% glycerol, and 1 mM DTT). Fractions containing pure ASP3 were pooled, concentrated to 1 mg/mL and flash frozen in liquid nitrogen.

### Recombinant RON13 purification

All RON13 constructs and mutants used for structural and biochemical studies were expressed in insect cells and purified, following the same procedure. RON13 coding sequence (amino acids 271–1375) was inserted into a modified pFastBac vector encoding a N-terminal Melittin secretion signal, a GST-tag and a TEV recognition site preceding RON13, and a C-terminal 10-His tag. Proteins were expressed in baculovirus-infected Sf9 cells for 66 h at 27 °C before cell harvesting and purification. The pellet from 1 L of cells was resuspended in 60 mL of Sf9 lysis buffer (50 mM Tris-HCl pH 7.5, 300 mM NaCl, 5 mM EDTA, and 3 mM 2-ME) containing protease inhibitors and lysed by one passage through a French Pressure Cell at 1500 p.s.i. Cell membranes and debris were removed by centrifugation at 35,000 × *g* for 30 min at 4 °C and soluble proteins were applied to 5 mL (bed volume) of gluthatione-agarose resin equilibrated in Sf9 lysis buffer. The sample was incubated for 4 h at 4 °C on a roller, then transferred to a gravity purification column to remove unbound material. The resin was washed with 50 mL of Sf9 lysis buffer followed by another 50 mL wash with Sf9 wash buffer (20 mM Tris pH 7.5, 100 mM NaCl, 0.5 mM EDTA, and 3 mM 2-ME). On-resin digestion was performed to elute the untagged RON13 protein from the resin. For this, 400 µg of pure TEV protease was diluted in 5 mL of Sf9 wash buffer and applied to the resin. After 2 h incubation at 22 °C, the digested protein was eluted by applying twice 7 mL of Sf9 wash buffer. The TEV protease was separated from RON13 using an anion-exchange column. Proteins eluted were concentrated to 3 mL using AMICON 30 MWCO concentrators, diluted fourfold with a 100 mM Tris-HCl pH 8 solution, and passed through a 1 mL Q-XL column (GE healthcare). The unbound material, containing RON13, was concentrated to 1 mL using AMICON 30 MWCO concentrator and loaded on a Superdex 200 10/300 size-exclusion chromatography column equilibrated in Sf9 final buffer (5 mM Tris-HCl pH 7.5/200 mM NaCl/0.5 mM EDTA/3 mM DTT). Fractions containing pure RON13 protein, eluting ~13 mL, were pooled, concentrated, and flash frozen in liquid nitrogen before storage at −80 °C.

### Recombinant dephosphorylated RON4 purification

RON4 FL (gene TGGT1_229010 product, residues 27–984) purification was performed using Ni^2+^ metal affinity purification^5^. Sequence of RON4 (27–984) with a C-terminal His_6_ tag was inserted into a modified pFastBac vector encoding a N-terminal gp67 secretion signal. Baculoviruses encoding RON4 protein were generated following standard procedures. Briefly, a pellet of Sf9 cells infected for 70 h was resuspended in lysis buffer 4 (50 mM Tris pH 8, 500 mM NaCl, 20 mM imidazole, and 3 mM 2-ME), lysed by shear forces at 15,000 p.s.i. using a Microfluidizer instrument. Cell membranes and debris were removed by centrifugation at 35,000 × *g* for 30 min at 4 °C, and soluble proteins were passed through a 5 mL His-trap FF column, washed with 50 mL of lysis buffer 4, and eluted with 15 mL of lysis buffer 4 supplemented with 350 mM imidazole. The protein was dephosphorylated using 400 units of alkaline phosphatase (CIAP, Promega # M1821A, 1 U/μL) in a buffer supplemented with 10 mM MgCl_2_ prior to running a Superdex 200 size-exclusion chromatography step equilibrated in SEC buffer 4 (25 mM Tris pH 7.5, 150 mM NaCl, and 0.5 mM EDTA) to separate RON4 protein from the phosphatase.

### Radioactive kinase assays

RON13 in vitro kinase activity was measured by looking at the transfer of γ-32P from ATP to substrate proteins. Reactions were run in 15 μL volume with a final RON13 concentration of 50 nM in kinase reaction buffer (20 mM Tris pH 7.5/50 mM NaCl/0.5 mM EDTA/0.5 mM EGTA). Reactions were supplemented with 6 mM of metal ion cofactor (MgCl_2_ for Mg^2+^, CaCl_2_ for Ca^2+^, and MnCl_2_ for Mn^2+^). Substrates were used at a final 1.5 μM concentration (MBP, dephosphorylated, Millipore #13-110). Reactions were initiated by the addition of tenfold concentrated ATP (100 μM final) containing 5 μCi of ATP-γ−32P. Reactions were run for 5 min at 37 °C. Phosphotransfer activity was monitored by analyzing 5 μL of reaction sample on a SDS–PAGE and the amount of protein phosphorylation was determined by exposing the gel to a X-ray sensitive plate. ATPase activity was measured by spotting 1 μL of reaction onto a TLC plate. ATP was separated from free phosphate by migrating the silica plate using a solution of 1 N formic acid/0.5 N LiCl as eluent. Exposure to an X-ray sensitive plate was used to measured radioactive 32P on a Typhoon instrument (GE healthcare). Experiments were done in triplicate.

### Hydrogen/deuterium exchange coupled to mass spectrometry

HDX-MS experiments were performed at the UniGe Protein Platform (University of Geneva, Switzerland) following a well-established protocol with minimal modifications^41^. HDX reactions were done in 50 μL volumes using 60 pmol (1.3 μM) of rRON13dk and a threefold molar excess of recombinant RON4. Briefly, rRON13dk was preincubated with buffer (RON13-alone) or RON4 on ice in a final volume of 7.7 μL. The mix was then left to equilibrate at the room temperature for 10 min. Deuterium exchange reactions were initiated by adding 42.3 µL of D2O exchange buffer (96% D2O, 10 mM HEPES pH 7.5, 100 mM NaCl, and 0.5 mM EDTA) to the rRON13 protein mixture. Reactions were carried-out for 3, 30, and 300 s at room temperature, and terminated by the sequential addition of 20 μL of ice-cold quenching buffer 1 (6 M urea/0.1 M NaH_2_PO_4_ pH 2.5/1% formic acid). Samples were immediately frozen in liquid nitrogen and stored at −80 °C for up to 4 weeks. All experiments were repeated in triplicates.

To quantify deuterium uptake, protein samples were thawed and injected in UPLC system immersed in ice. The protein was digested via two immobilized pepsin columns (Thermo #23131), and peptides were collected onto a VanGuard pre-column trap (Waters). The trap was subsequently eluted and peptides separated with a C18, 300 Å, 1.7 μm particle size Fortis Bio column 100 × 2.1 mm over a gradient of 8–30% buffer B over 20 min at 150 μL/min (buffer A: 0.1% formic acid; buffer B: 100% AcN). Mass spectra were acquired on an Orbitrap Velos Pro (Thermoscientific), for ions from 400 to 2200 *m*/*z* using an electrospray ionization (ESI) source operated at 300 °C, 5 kV of ion spray voltage. Peptides identified by data-dependent acquisition after MS/MS and data were analyzed by Mascot. A 94% sequence coverage was obtained. Deuterium incorporation levels were quantified using HD examiner software version 1.4 (Sierra Analytics), and quality of every peptide was checked manually. Results are presented as percentage of theoretical maximal deuteration level and can be compared to a highly-deuterated sample that was prepared by incubating the protein for 1 h in 1 M guanidinium-HCl before incubation for 2 h in deuterated buffer. All experimental details and data of percentage deuterium incorporation for all peptides can be found in Supplementary Table 3 and Supplementary Data 6. The HDX-MS data have been deposited on the ProteomeXchange Consortium via the PRIDE^42^ partner repository with the dataset identifier PXD023791.

### Cryo-EM sample preparation and data collection

The purified RON13-KD was concentrated (15 mg/mL) and immediately used for cryo-EM grid preparation. For cryo-EM sample preparation, Quantifoil 1.2/1.3 200-mesh grids were glow discharged using a PELCO easiGlow (Ted Pella) for 30 s at 25 mA in air. An aliquot of the protein (3.5 µL) was deposited on the surface of the grid immediately before blotting for 3 s on a Vitrobot Mark IV (FEI) operating at 4 °C with 100% humidity (blot force 0) and plunging the grid into liquid ethane. The grids were transferred into liquid nitrogen for storage until the day of cryo-EM data collection. A cryo-EM dataset was collected at using Titan Krios microscope equipped with a Gatan K2 Summit detector and an energy filter at ETH Zurich (ScopeM). The dataset was collected in counting mode using EPU, at a magnification of 165,000×, corresponding to a pixel size 0.8544 Å/pix. The exposure was 8 s over 40 frames, with a total dose of 47 e^−^/Å^2^.

### Cryo-EM data analysis

Motion correction and alignment of the cryo-EM movies was performed using MotionCor2 (UCSF; version 1.3.0)^43^; the defocus of the aligned micrographs was estimated using Gctf (version 1.06)^44^. The images with an estimated resolution higher than 4.5 Å (4782 micrographs) were used in the further processing steps; all subsequent steps were performed in relion-3.0 (refs. ^45,46^). Manual particle picking and 2D classification of a small subset of particles (732) was used to generate four autopicking templates. In total 1,385,124 particles were autopicked and extracted from the selected micrographs (downsampled to 2.56 Å/pix in a 100 pix box). Two rounds of 2D classification produced 2D classes with clearly visible secondary structure elements (711,852 particles). An initial model generated using this particle selection in relion-3.0 was used for 3D classification. A round of 3D classification with four classes (*T* = 4, *E* = 8, mask 190 Å) produced a single class (348,152 particles) was followed by a round of masked 3D classification without alignment (*T* = 4, *E* = −1) generating a 3D class used for further processing (320,723 particles). The particles constituting this 3D class were used in 3D refinement (3.4 Å resolution, FSC cutoff 0.143). Further CTF refinement and Bayesian particle polishing, followed by 3D refinement and a post-processing step (applying a *b*-factor of −85.7 estimated in relion-3.0), produced the final map 3.1 Å resolution. The local resolution of the map was estimated in relion-3.0. The anisotropy of the cryo-EM map was assessed using the 3DFSC server^47^. Slight anisotropy was detected (Supplementary Fig. 4i), without a substantial effect on the quality of the density map (global resolution reported by 3DFSC is 3.09 Å, consistent with the results of Relion processing). The complete cryo-EM data analysis procedure is illustrated in Supplementary Fig. 4.

### Model building and validation

An initial round of autobuilding was performed using phenix.map_to_model (Phenix 1.16-3549)^48^. All subsequent model building steps were performed in Coot (version 0.9.5)^49^ using the 3.1 Å post-processed map. The atomic model of RON13-KD was refined using phenix.real_space_refine in Phenix (version 1.16-3549). For model validation, the atom coordinates in the refined model were randomLy displaced by a 0.5 Å using the PDB tools in Phenix and the derived model was subjected to real space refinement using one of the refined half maps (half-map1). Map versus model FSC comparison was made for the model against the corresponding half-map1 used in the refinement job, and for the same model versus the half-map2 (not used during refinement)^50^. The geometry of the model was validated using MolProbity (version 4.5.1)^51^. Figure panels featuring the density maps and structural models were prepared using PyMol (DeLano Scientific LLC, version 4.2.4)^52^ and UCSF Chimera^53^ (UCSF; version 1.13.1).

### Phosphoproteome sample preparation

*T. gondii* tachyzoites were cultured using HFFs as host cells. After 48 h, the supernatant was removed and syringe homogenized prior to differential centrifugation at 200 × *g* for 5 min to pellet host cells. The supernatant containing extracellular parasites was filtered to remove cell debris and the parasites were washed five times in PBS containing protease inhibitors. Samples were prepared for each strain from four independent experiments. Proteins were extracted from tachyzoites in 4% SDS/0.1 M DTT/100 mM Tris-Cl pH 8.2 first by boiling at 95 °C for 5–10 min and following by at least one round of exposure to high-intensity focused ultrasound for 1 min at 100% amplitude and 80% duty cycle. Insoluble material was pelleted by high-speed centrifugation and 190–300 µg of proteins in 60 µL of 4% SDS/0.1 M DTT/100 mM Tris-Cl pH 8.2 were further processed using a modified protocol for filter-aided sample preparation^54^. A total of 400 µL of 8 M urea/100 mM Tris-Cl pH 8.2 were added, and the sample was loaded onto a Microcon-30kDa Centrifugal Filter Unit (Merck) and centrifuged at 14,000 × *g* until ~10 µL remained. The sample was washed with 200 µL 8 M urea/100 mM Tris-Cl pH 8.2 and subsequently, 100 µL 50 mM iodoacetamide/8 M urea/100 mM Tris-Cl pH 8.2 was added. The solution was shaken at 600 r.p.m. for 1 min and incubated for 5 min at RT. The sample was spun at 14,000 × *g* and washed three times with 100 µL 8 M urea/100 mM Tris-Cl pH 8.2. The sample was then washed twice with 50 mM of triethylammoniumbicarbonate (TEAB) in H_2_O and spun at 14,000 × *g*. A total of 120 µL of trypsin in 50 mM TEAB at 1/50 w/w was added to the sample, mixed at 600 r.p.m. for 1 min, and incubated in a wet chamber overnight. Peptides were collected by centrifugation at 14,000 × *g*, a 5 µL aliquot was removed as input sample and the peptides concentrated by vacuum centrifugation. For the following steps, all the incubation steps were done while shaking at 700 r.p.m. at RT and supernatants removed after bead attachment to a magnet. A 50 µL of Ti-IMAC beads (ReSyn Biosciences) per sample were washed twice with 200 µL 70% ethanol for 5 min, and subsequently once with 100 µL 1% NH_4_OH 10 min. Beads were equilibrated three times for 1 min in 50 µL loading buffer (380 mg glycolic acid/5% trifluoroacetic acid (TFA)/80% AcN). Peptides were taken up in 100 µL loading buffer and incubated 20 min. Beads were washed in 100 µL loading buffer for 30 s and washed three times with 100 µL 1% TFA/80% AcN for 2 min, and then twice with 0.2% TFA/10% AcN for 2 min. Phosphopeptides were eluted three times in 80 µL 1% NH_4_OH for 15 min. Samples were vacuum dried and resuspended in 15 µL 0.1% formic acid/3% AcN. To the input sample, 10 µL 0.1% formic acid/3% AcN was added.

### Reverse phase chromatography and mass spectrometry

For peptide separation by reverse phase chromatography, a Waters ACQUITY UPLC M-Class was used with a 20 mm nanoEase M/Z Symmetry C18 trap column (180 µm inner diameter packed with 5 µm C_18_ silica particles), and a 25 cm nanoEase M/Z HSS C18 T3 column (75 µm inner diameter and packed with 1.8 µm C_18_ silica particles), and analytical column. Total separation time was 75 min over a linear gradient from 5 to 35% solvent B (solvent A: 0.1% formic acid in H_2_O, solvent B: 0.1% formic acid in AcN) with a flow rate of 300 nL/min. Separated peptides were directly applied by ESI into an Orbitrap Q-Exactive HF mass spectrometer (Thermo Fisher Scientific). The mass spectrometry was set up in full MS/data-dependent-MS^2^ mode. For the full scans, a scan range of 350–1500 *m*/*z* at a resolution of 120,000 was used with an automatic gain control (AGC) target at 3 × 10e6 and a maximum injection time of 50 ms. The top 20 most intense ions were isolated with a maximum injection time of 119 ms, fragmented with a NCE of 28 and detected at 60,000 resolution with a scan range of 200–2000 *m*/*z* and an AGC target of 1 × 10e5 (fixed first mass 130 *m*/*z*).

### Phosphoproteome data analysis

Individual data analysis workflows have been used for global protein and phosphopeptide analysis. The statistical analysis was done in a two-group comparison, i.e., RON13-KD versus RH and RON13-KD complemented with WT RON13 versus RON13-KD complemented with inactive RON13. For proteome and phosphoproteome, the acquired raw MS data were processed by MaxQuant (version 1.6.2.3), followed by protein identification using the integrated Andromeda search engine^55^. Spectra were searched against the ToxoDB GT1 protein database (release 44), concatenated to its reversed decoyed fasta database and common protein contaminants. By default, carbamidomethylation of cysteine was set as fixed modification, while methionine oxidation and N-terminal protein acetylation were set as variable. In addition, Phospho(STY) was set as a variable modification. Enzyme specificity was set to trypsin/P allowing a minimal peptide length of seven amino acids and a maximum of two missed-cleavages. For the proteome data, MaxQuant default search settings for Orbitrap were used. The maximum FDR was set to 0.01 for peptides and 0.05 for proteins. Label-free quantification was enabled and a 2 min window for match between runs was applied. In the MaxQuant experimental design, each file is kept separate to obtain individual quantitative values. Protein fold changes were computed based on intensity values reported in the proteinGroups.txt file. A set of functions implemented in the R package SRMService^56,57^ was used to filter for proteins with two or more peptides allowing for a maximum of four missing values, normalizing the data with a modified robust *z*-score transformation and computing *P* values using the moderated *t* test with pooled variance (as implemented in the limma package^58^). If all measurements of a protein are missing in one of the conditions, a pseudo fold change was computed replacing the missing group average by the mean of 10% smallest protein intensities in that condition. For the phosphoproteome data, each file was kept separate in the experimental design to obtain individual quantitative values. Precursor and fragment tolerance was set to 10 and 20 p.p.m., respectively for the initial search. The maximum FDR was set to 0.01 for peptides and 0.05 for proteins. Label-free quantification was enabled and a 2 min window for match between runs was applied. The re-quantify option was selected.

For the phosphosite analysis, a similar data analysis strategy as described by Sharma et al.^59^ was implemented. In brief, the MaxQuant phospho_STY_site.txt file was used as the input file. The phosphosite table was expanded with respect to their multiplicity and filtered for a minimum localization site probability of 0.75. For each two-group comparison, all peptides with a maximum of four missing values were retained. The data (like for the total proteome) was normalized with a modified robust *z*-score transformation and *P* values were computed with a moderated *t* test with pooled variance (as implemented in the limma package^58^). If all measurements of a protein were missing in one of the conditions, a pseudo fold change was computed replacing the missing group average by the mean of 10% smallest protein intensities in that condition. Calculated *P* values are adjusted for multiple testing using the BH-method. The statistics of the phosphopeptide analysis and the total proteome analysis were merged. The mass spectrometry proteomics data have been deposited to the ProteomeXchange Consortium via the PRIDE^36^ partner repository with the dataset identifier PXD018056.

### Data parsing and analysis

For comparison to the existing phosphoproteome^13^, phosphopeptide data from all strains in this study, and data from purified tachyzoites and infected host cells (phosphopeptide-enriched and -depleted) were pooled. The ToxoDB gene IDs, as well as phosphosites from each study were then intersected and plotted as a Venn diagram. To identify potential targets of RON13, the comparison of RON13-KD to RH (dataset 1), as well as of RON13-KD/ron13wt and RON13-KD/ron13dk (dataset 2) were used. Peptide contaminants and peptides mapping to reverse proteins were removed. Phosphopeptides with at least fourfold more abundance in either comparison (log_2_FC < −2) were selected for further analysis. Peptides with the posterior error probabilities (PEPs) > 0.05 and with *P* values (associated to the log_2_FC) were >0.05 were excluded. The common hits between both datasets were then considered high-confidence candidates of RON13. Differences in multiplicity of phosphorylation were not taken into account if position of the phosphorylated amino acid was identical. The polar plot was built by slightly modifying the R package funscoR (https://github.com/evocellnet/funscoR)^60^. The phosphoproteome data have been deposited on the ProteomeXchange Consortium via the PRIDE^42^ partner repository with the dataset identifier PXD018056.

### Determination of phosphoserine sequence logo

To determine the sequence logos for phosphoserine, the phosphopeptides corresponding to RON13 substrates (Supplementary Data 5) containing phosphoserines were used. The flanking 15 amino acids of the phosphoserines were extracted, and the resulting 31 amino acid sequences were used as an input for Sequence Logo Analysis tool at PhosphoSitePlus^61^ (www.phosphosite.org, version 6.5.9.3). PSP production was used as an algorithm and “Phoshpo Ser” used as background. The consensus sequence was computed using EMBOSS^62^ (https://www.ebi.ac.uk/Tools/msa/emboss_cons/).

### Reporting summary

Further information on research design is available in the Nature Research Reporting Summary linked to this article.

## Supplementary information

Supplementary_information

Description of Additional Supplementary Files

Supplementary Data 1

Supplementary Data 2

Supplementary Data 3

Supplementary Data 4

Supplementary Data 5

Supplementary Data 6

Supplementary Data 7

Supplementary Data 8

Supplementary Movie 1. Rhoptry morphology of control RH sample.

Supplementary Movie 2. Rhoptry morphology in ASP3 depleted parasites.

Supplementary Movie 3. The atypical structure of RON13 kinase.

Reporting Summary

## Data Availability

All data are present in the main text and the supplementary materials. The mass spectrometry proteomics regarding RON4 immunoprecipitation have been deposited on the ProteomeXchange Consortium via the PRIDE^42^ partner repository with the dataset identifier PXD021516. The phosphoproteomic data have been deposited on the ProteomeXchange Consortium via the PRIDE^42^ partner repository with the dataset identifier PXD018056. The HDX-MS data have been deposited on the ProteomeXchange Consortium via the PRIDE^42^ partner repository with the dataset identifier PXD023791. The coordinates and the cryo-EM map have been deposited to the Protein Data Bank (PDB ID: 7NUR) and Electron Microscopy Data Bank (accession code: EMD-12600). The public dataset used for mass spectrometry analysis of RON4 immunoprecipitation is the ToxoDB_Tgondii_GT1 database (http://ToxoDB.org, release 42) and the one used for the phosphoproteomics analysis is the ToxoDB_Tgondii_GT1 database (http://ToxoDB.org, release 44). All biological materials and data are available from the authors upon request. Source data are provided with this paper.

## Source data

Source Data File
