# Supplementary material for: Structural insights into an atypical secretory pathway kinase crucial for Toxoplasma gondii invasion
Source: Nat Commun. 2021 Jun 18;12:3788. doi: 10.1038/s41467-021-24083-y (PMC8213820; doi:10.1038/s41467-021-24083-y)
Supplement: Supplementary file 1 — Supplementary_information [file 41467_2021_24083_MOESM1_ESM.pdf]

Supplementary Information for

Structural insights into an atypical secretory pathway kinase crucial for *Toxoplasma gondii* invasion

Gaëlle Lentini1†, Rouaa Ben Chaabene1†, Oscar Vadas1†, Chandra Ramakrishnan2, Budhaditya Mukherjee1,3, Ved Mehta4, Matteo Lunghi1, Jonas Grossmann5,6, Bohumil Maco1, Rémy Visentin1, Adrian B. Hehl2, Volodymyr M. Korkhov4,7,* and Dominique Soldati-Favre1,*

Correspondence to: dominique.soldati-favre@unige.ch, volodymyr.korkhov@psi.ch

†These authors contributed equally to the work

This PDF file includes:

Supplementary Figures 1 to 10
Supplementary Tables 1 to 6
Supplementary references

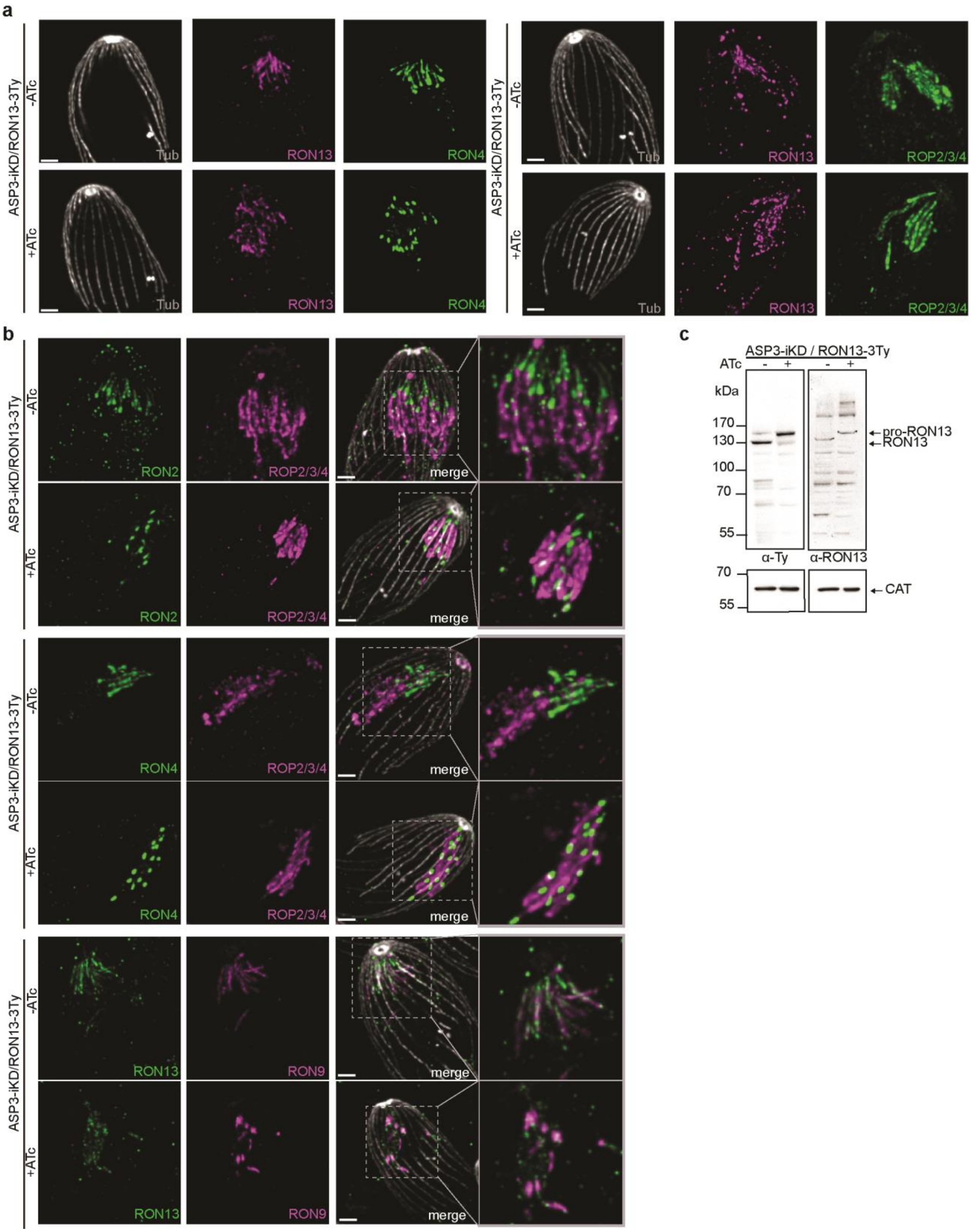
a
ASP3-iKD/RON13-3Ty
-ATc
+ATc
Tub
RON13
RON4
ROP2/3/4
b
RON2
RON9
merge
c
ASP3-iKD / RON13-3Ty
ATc
kDa
170
130
100
70
55
pro-RON13
RON13
α-Ty
α-RON13
CAT

Supplementary Fig. 1. ASP3 depletion impacts rhoptry morphology and content localization. a. U-ExM images of parasites from ASP3-iKD/RON13-3Ty extracellular parasites ± anhydrotetracycline (ATc). The images presented here correspond to the individual channel merged in Figure 1a. ROP2/3/4 (green) antibodies are used to visualize the bulb of the rhoptries while RON4 (green) stains the neck of the rhoptries. RON13 (magenta) is detected using anti-Ty antibodies and parasite subpellicular microtubules are detected with anti-α/β tubulin antibodies (grey). Scale bar = 2 µm. Images representative of three biologically independent experiments. **b.** U-ExM images of rhoptries from ASP3-iKD/RON13-3Ty extracellular parasites ±ATc. ROP2/3/4 (magenta) antibodies are used to visualize the bulb of the rhoptries while RON2 (green), RON4 (green), RON9 (magenta) antibodies stain the neck of the rhoptries. RON13 (green) is detected using anti-Ty antibodies. Scale bar = 2 µm. Images representative of three biologically independent experiments. **c.** Immunoblot on ASP3-iKD/RON13-3Ty parasite lysates ±ATc demonstrating that the RON13 antibodies generated recognize both pro and mature RON13. Catalase (anti-CAT) is used as a loading control. Image representative of three biologically independent experiments. Source data are provided as a Source data file.

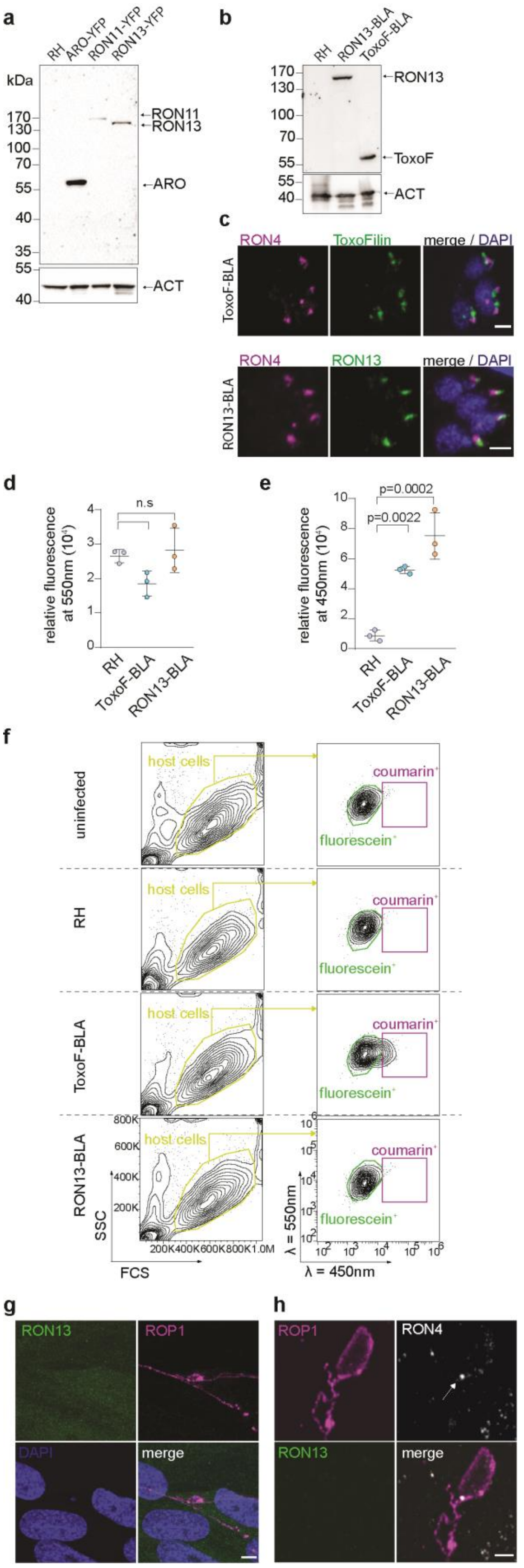
a
RH
ARO-YFP
RON11-YFP
RON13-YFP
kDa
170
130
100
70
55
40
35
55
40
RON11
RON13
ARO
ACT
b
RH
RON13-BLA
ToxoF-BLA
RON13
ToxoF
ACT
c
RON4
ToxoFilin
merge / DAPI
ToxoF-BLA
RON13
RON13-BLA
d
relative fluorescence at 550nm (10⁴)
n.s
RH
ToxoF-BLA
RON13-BLA
e
relative fluorescence at 450nm (10⁴)
p=0.0002
p=0.0022
f
uninfected
host cells
coumarin⁺
fluorescein⁺
SSC
FCS
λ = 550nm
λ = 450nm
g
ROP1
DAPI
merge
h
RON4

19

Supplementary Fig. 2. RON13 is not secreted during invasion. a. Immunoblot on RH and ARO-YFP, RON11-YFP and RON13-YFP transgenic parasites showing proper expression of the fusion protein used in the topology assay. Actin (ACT) is used as a loading control. Image representative of three biologically independent experiments. **b.** Immunoblot showing the fusion of ToxoFilin and RON13 with the beta-lactamase protein (BLA). Anti-HA antibodies were to detected the fusion protein ToxoFilin-HA-BLA (ToxoF-BLA) and anti-myc antibodies were used to detect the fusion protein RON13-4myc-BLA (RON13-BLA). Image representative of three biologically independent experiments. **c**. ToxoF-BLA (green) and RON13-BLA (green) fusion proteins are properly targeted to the rhoptries as shown by IFA on intracellular parasites using anti-HA antibodies. Anti-HA antibodies were used to detect the fusion protein ToxoF-BLA and anti-myc antibodies to detect the fusion protein RON13-BLA. Scale bar = 2µm. Images representative of three biologically independent experiments. RON4 (magenta). DAPI (blue). **d and e**. Lactamase activity assessed on extracellular parasites demonstrating that the beta-lactamase is active when fused to ToxoFilin or RON13. The relative fluorescence of extracellular parasites incubated with the beta-lactamase substrate at 550nm (**d**) and 450nm (**e**) is shown for RH, ToxoF-BLA and RON13-BLA strains. This experiment was performed in triplicate. One ANOVA followed by Tukey's multiple comparison was used to test differences between groups (mean ± SD; n=3 biologically independent experiments). **f**. Gating strategy for quantification of fluorescein$^+$ cell (λ=550nm; green gate) and coumarin$^+$ cell (λ=450nm; violet gate) frequency for uninfected cell monolayer, RH, RON13-BLA and ToxoFilin-BLA infected cell monolayer (yellow gate) analyzed by flow cytometry. The gating strategy for RON13-BLA and ToxoFilin-BLA is also shown in Figure 2c.The frenquencies of fluorescein$^+$ cell

(λ=550nm; green gate) and coumarin$^+$ cell (λ=450nm; violet gate) were used to generate the graph shown in Figure 2d and 2e respectively. This experiment was performed in triplicate (n=3 biologically independent experiments). **g.** IFAs of extracellular parasites treated with cytochalasin D to block invasion but not rhoptry secretion. E-vacuoles are visualized with anti-ROP1 antibodies (magenta) and DAPI (blue) is used to stain DNA. Anti-Ty antibodies failed to detect RON13 (green) post-secretion. Scale bar = 5 µm. Images representative of three biologically independent experiments. **h.** IFAs of e-vacuole rhoptry secretion assay on RON13-3Ty parasites. ROP1 (magenta) is detected in the e-vacuoles post-secretion using anti-ROP1 antibodies and RON4 (white) is detected as a dot post-secretion using anti RON4 antibodies (arrow). RON13 (green) is not detected in the host cell post-secretion using anti-Ty antibodies. Scale bar = 2 µm. Images representative of three biologically independent experiments. Source data are provided as a Source data file.

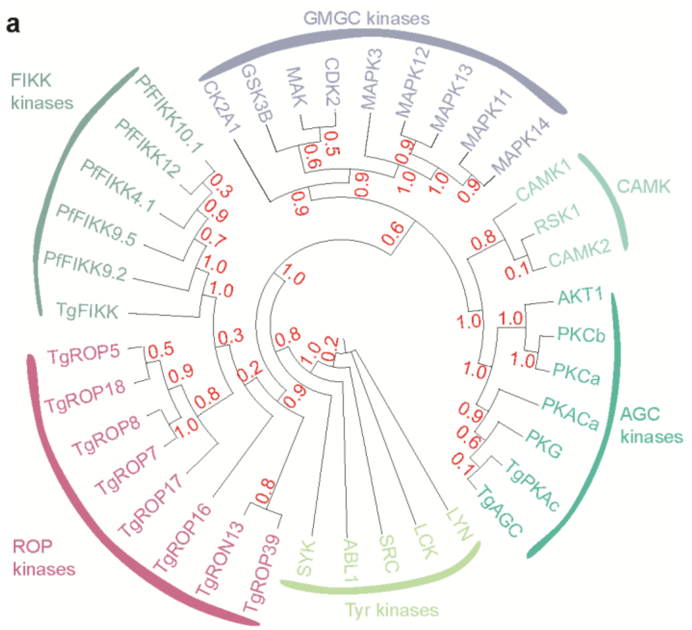

Supplementary Figure 3. RON13 belongs to the ROPK clade. Maximum-likelihood phylogenetic tree estimated from the multiple sequence alignment of the indicated kinases from different families (Supplementary Data 8). Bootstrap values are indicated in red.

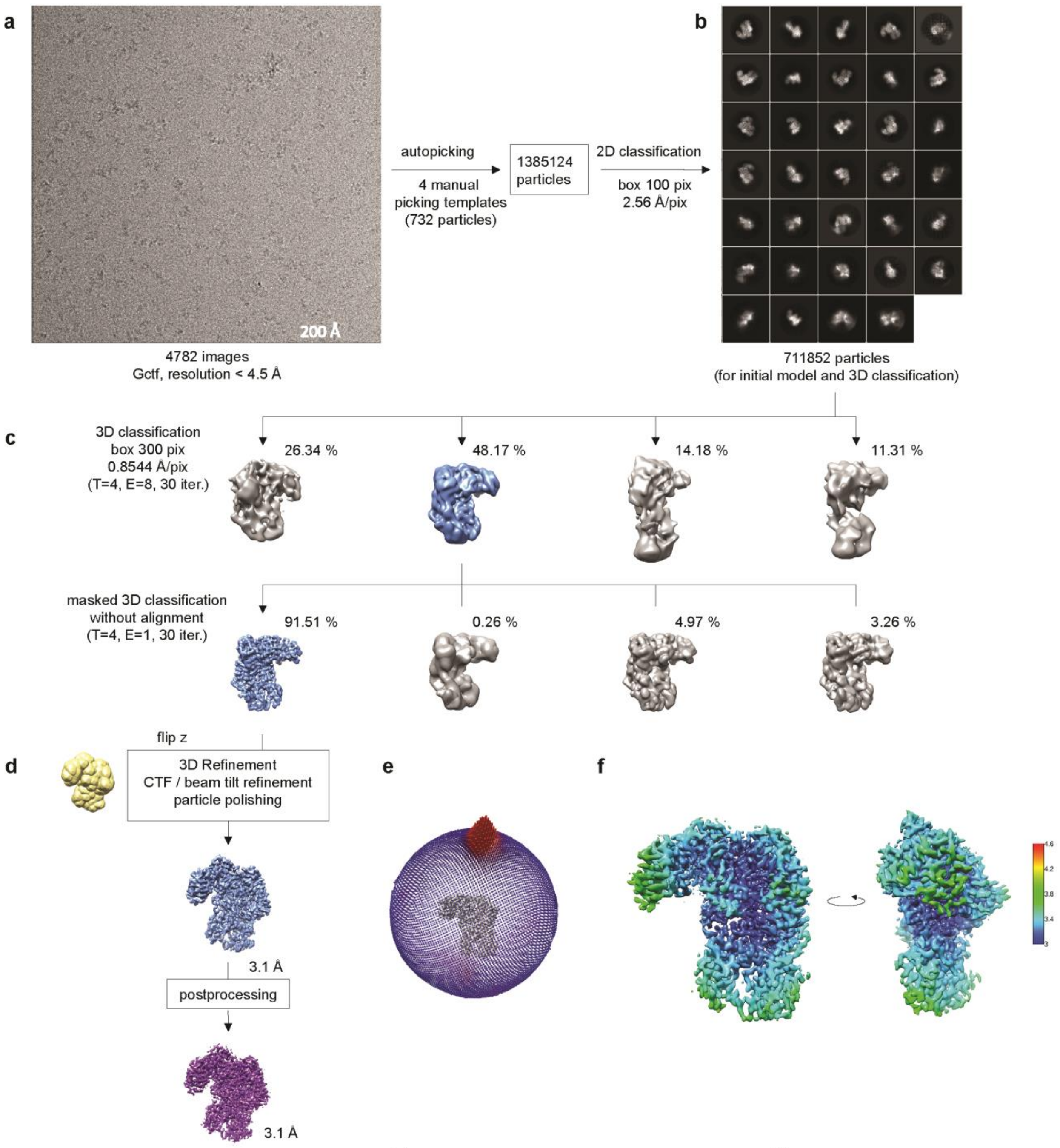
a
200 Å
4782 images
Gctf, resolution < 4.5 Å
autopicking
4 manual
picking templates
(732 particles)
1385124
particles
2D classification
box 100 pix
2.56 Å/pix
b
711852 particles
(for initial model and 3D classification)
c
3D classification
box 300 pix
0.8544 Å/pix
(T=4, E=8, 30 iter.)
26.34 %
48.17 %
14.18 %
11.31 %
masked 3D classification
without alignment
(T=4, E=1, 30 iter.)
91.51 %
0.26 %
4.97 %
3.26 %
flip z
d
3D Refinement
CTF / beam tilt refinement
particle polishing
3.1 Å
postprocessing
3.1 Å
e
f

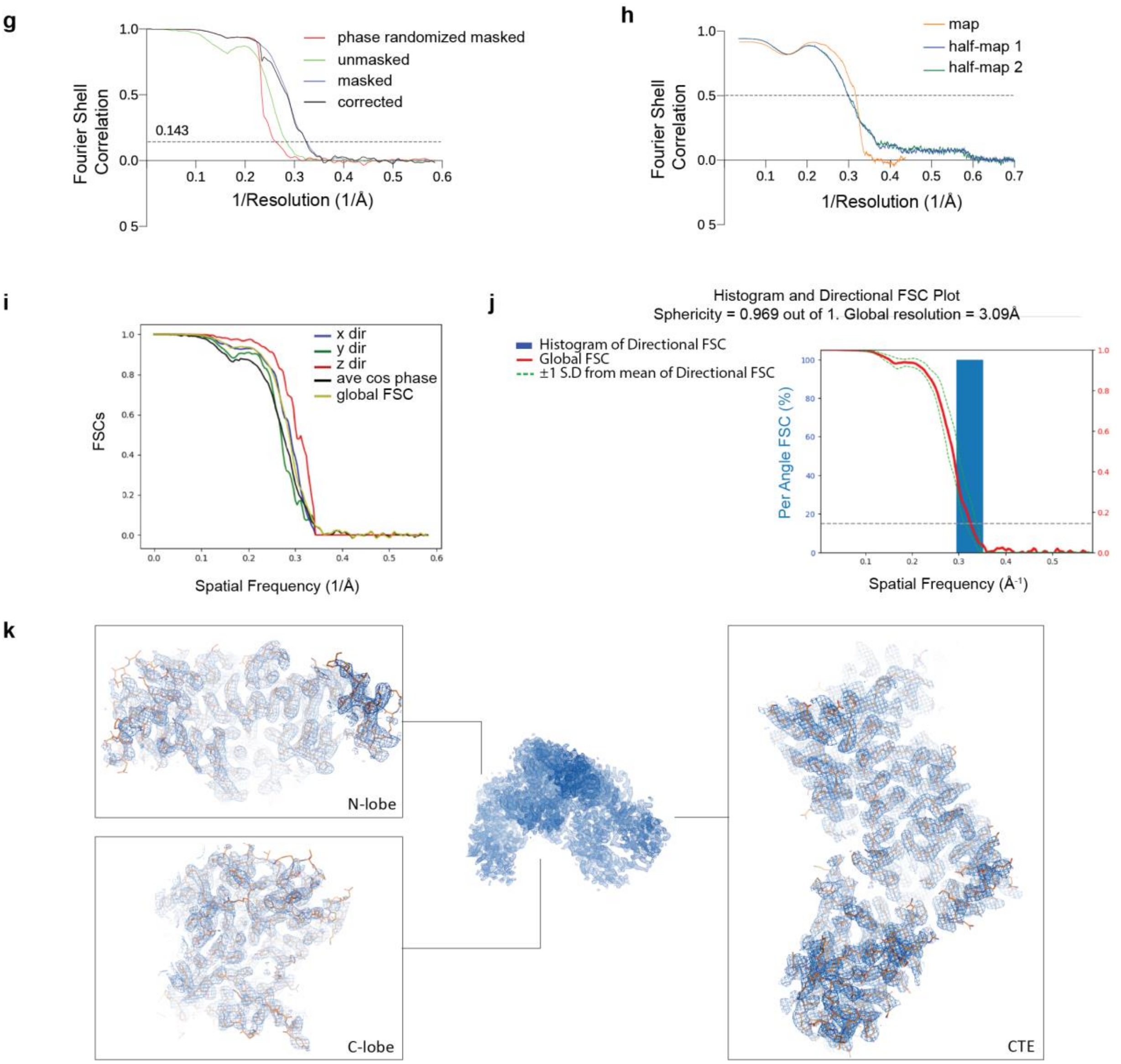

Supplementary Fig. 4. Cryo-EM and single particle analysis of rRON13dk. a. A representative motion-corrected micrograph of rRON13dk (scale bar corresponds to 200Å). **b.** A selection of the best 2D classes (bottom; box edge corresponds to 256 Å). **c.** Several rounds of 3D classification resulted in the final selection of particles for 3D refinement (blue). **d**. The z-flipped 3D class and mask were used for 3D refinement, followed by CTF refinement and Bayesian particle polishing, as described in “Methods”. The final postprocessed density map at 3.1 Å resolution is shown in magenta. **e.** Angular distribution

histogram of the rRON13dk dataset used in the final refinement. **f**. Local resolution estimate for the refined density map, calculated in relion-3.0. **g.** Fourier shell correlation (FSC) plot for the 3D map shown in d, refined and postprocessed to a resolution of 3.1 Å (FSC 0.143). **h.** Model to map FSC curve. **i** and **j.** Output of the 3DFSC server, reporting the directional (**i**) and global FSC (**j**) of the cryo-EM reconstruction. The sphericity and global resolution are indicated in (**j**). **k.** The postprocessed density map of RON13-KD, contoured at 8σ. The views of the map in boxes corresponding to the kinase N-lobe, C-lobe and CTD are overlayed with the model of the protein in stick representation.

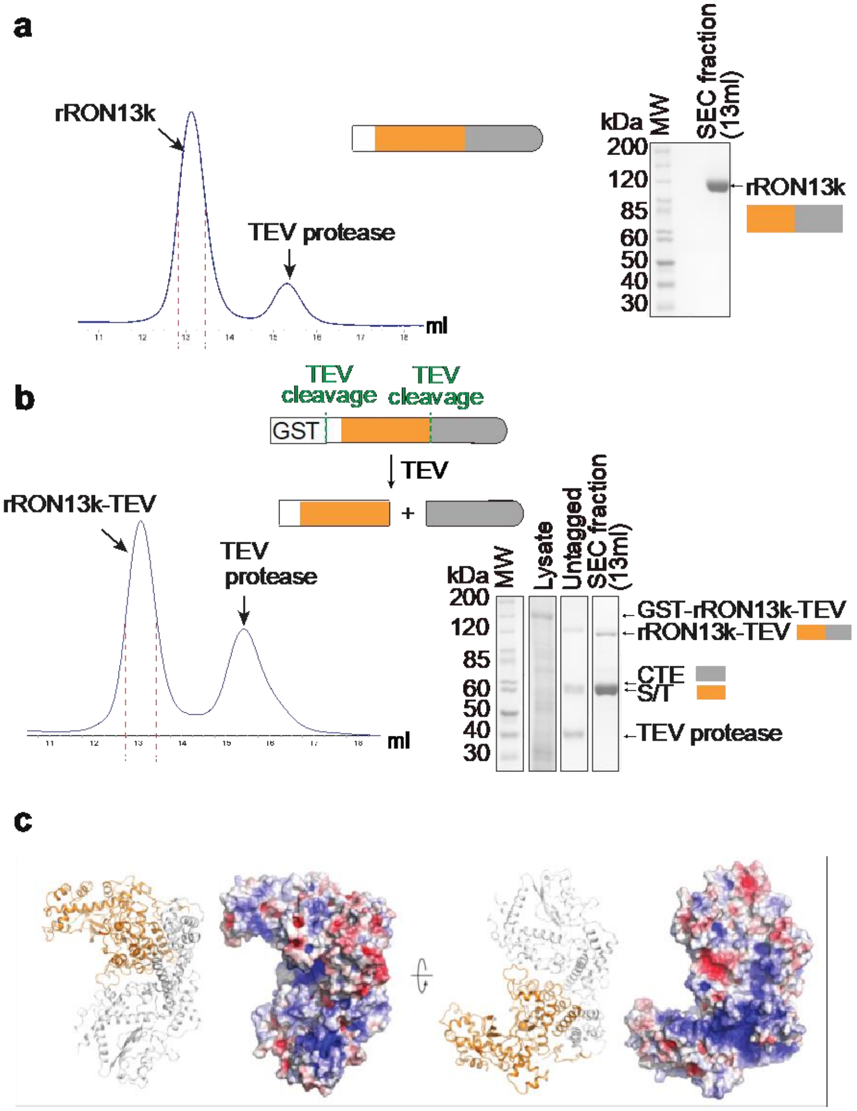

Supplementary Figure 5. RON13 CTE is essential for its folding and stability. a and **b.** Size-exclusion chromatography elution profiles of rRON13k protein (**a**) and a RON13 protein composed of individual kinase domain (orange) + CTE fragment (grey) (rRON13k-TEV) (**b**). The two fragments (kinase and CTE) were generated by cloning a TEV protease recognition site between the two domains, yielding the two expected fragments after purification and TEV proteolytic cleavage (SDS-PAGE analysis). Both rRON13wt and rRON13k-TEV behave identically on a Superdex 200 size-exclusion column. **c.** Cartoon and surface representation of RON13 from two opposite points of views; the sphere in the

cartoon representation indicates the position of the active site (residue 595). The protein surface is colored according to electrostatic potential, calculated using APBS (from red, -5 kT/e, to blue, +5 kT/e). Source data are provided as a Source data file.

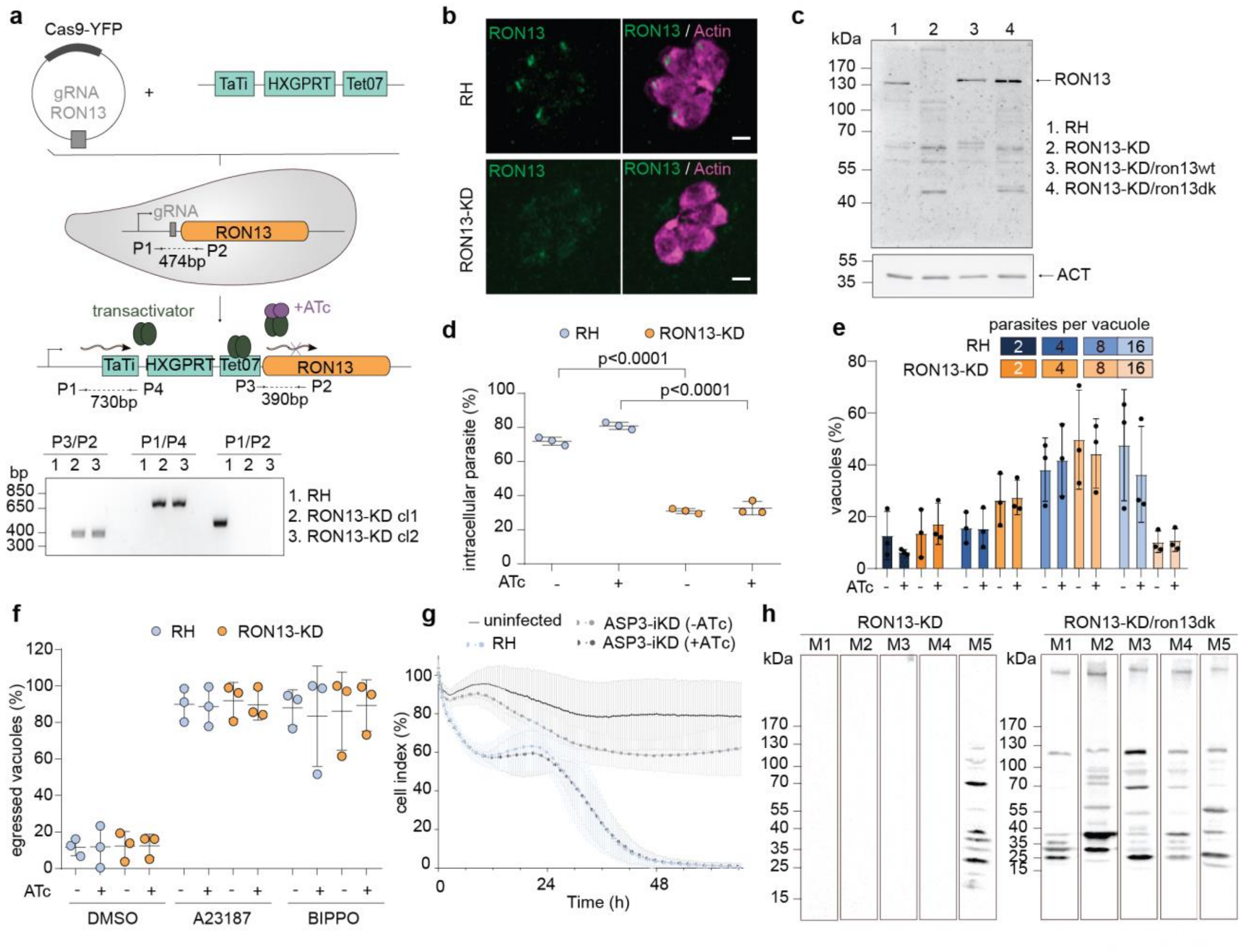

Supplementary Fig. 6. RON13 is an important invasion and virulence factor. a. Genetic strategy used to generate RON13-KD and integration PCRs on the RON13-KD clonal lines and RH parental line performed with primers P1/P4 (5' integration), P3/P2 (3' integration) and P1/P2 to control the locus (wt = 474 bp; modified locus = 4429 bp). **b.** IFAs of intracellular RH and RON13-KD parasites using anti-RON13 antibodies (green). An anti-actin antibody (magenta) stained the parasite cytoplasm. Scale bar = 2µm. Images representative of three biologically independent experiments. **c.** Western blot using anti-RON13 antibodies showing the downregulation of RON13 in RON13-KD parasites compared to the parental line RH or the complemented lines RON13-KD/ron13wt

(complemented wild-type) and RON13-DK/ron13dk (complemented dead kinase). Actin (anti-ACT) is used as a loading control. Image representative of three biologically independent experiments. **d.** Graph showing the percentage of intracellular parasites following 30 min of incubation with host cells reflecting the ability of extracellular parasites (±ATc) to invade. One ANOVA followed by Tukey's multiple comparison was used to test differences between groups (mean ± SD; n=3 biologically independent experiments). **e.** Intracellular replication assay. Graph representing the number of parasite per vacuole observed at 36 h post-invasion. Two-way ANOVA followed by Tukey's multiple comparison was used to test differences between groups (mean ± SD; n=3 biologically independent experiments). **f.** Induced egress assay. Graph representing the percentage of ruptured vacuoles following treatment with the egress inducers A23187 or BIPPO for RH and RON13-KD parasites (±ATc). Two-way ANOVA followed by Tukey's multiple comparison was used to test differences between groups (mean ± SD; n=3 biologically independent experiments). **g.** Kinetic assay representing the cell index of HFF infected with different parasite strains (mean ± SD; n=3 biologically independent experiments). **h.** Immunoblots showing the serology of infected mice at 84 days post-infection with RON13-KD and RON13-KD/ron13dk parasites (prior challenge). M1= mouse 1. Samples derive from the same experiment and gels were processed in parallel. The experiment was done once simultaneously with five mice for each strains tested (n=5 biologically independent animals). Source data are provided as a Source data file.

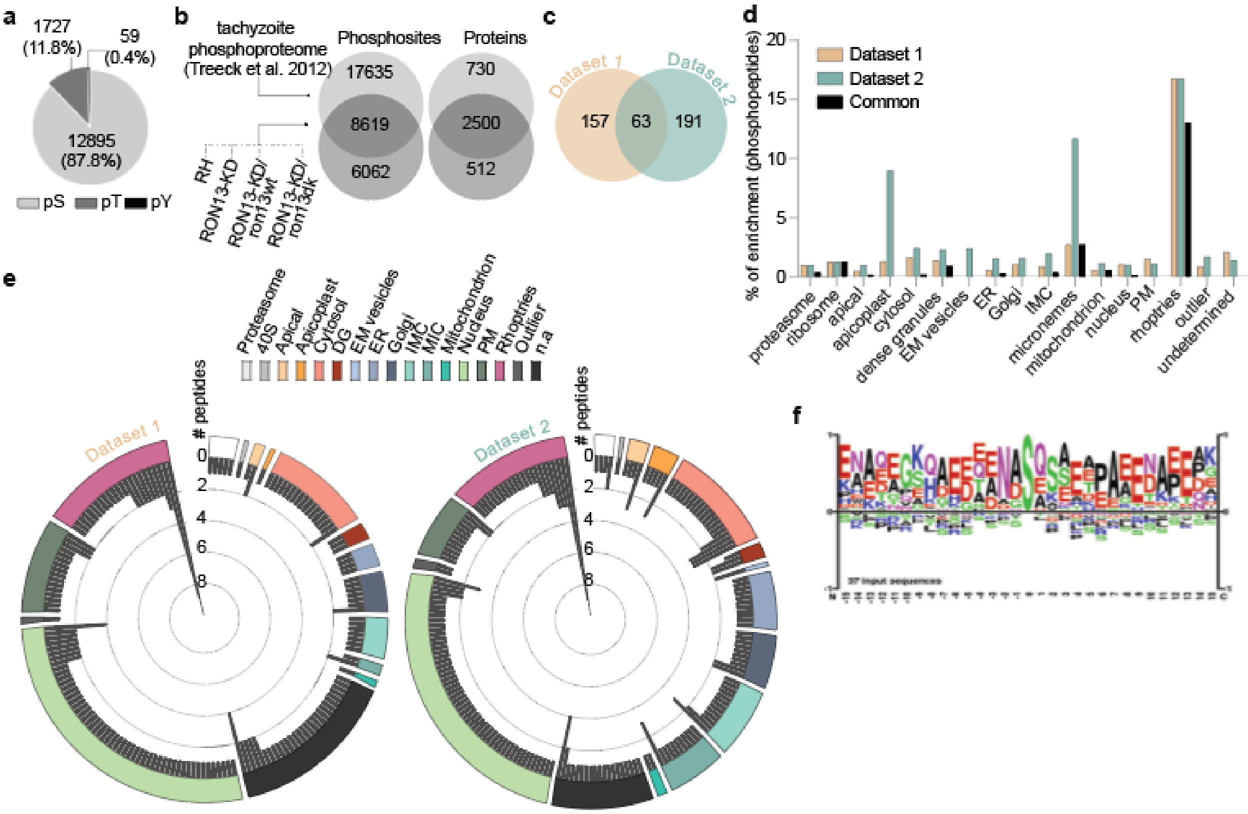

Supplementary Fig. 7. Rhoptry proteins are the major substrate of RON13. a. Pie chart showing the repartition of phospho-Ser (pS), phosphor-Thr (pT) and phosphor-Tyr (pY) among the identified phosphopeptides in the total phosphoproteome. Phosphoproteome analysis combined results obtained from four independent experiments. **b.** Venn diagrams showing the overlapping data in terms of proteins and phosphosites between the already published phosphoproteome [20] and the total phosphoproteome of this study. **c.** Venn diagram of the phosphosites found in Dataset 1 and Dataset 2. **d.** Bar graph showing the percentage of phosphopeptides for Datasets 1, Dataset 2 and common to both datasets relative to the total number of phosphopeptides according to their predicted localization[1]. **e.** Polar plots of the number of phosphopeptides found in Dataset 1 (left) and Dataset 2 (right) binned by gene IDs and clustered according to their predicted subcellular localization[1]. **f.**

Sequence logo for phosphoserine of RON13 substrates (37 input sequences). Position 0 indicates the serine that is phosphorylated. Source data are provided as a Source data file.

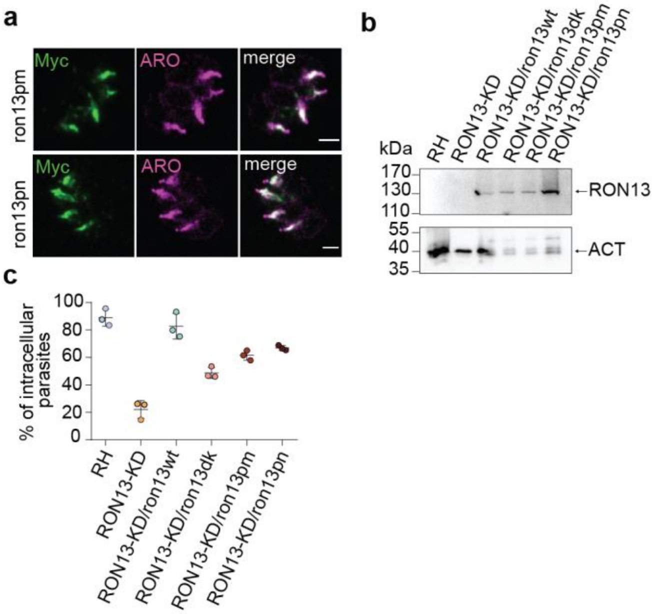

Supplementary Fig. 8. Phosphorylation of RON13 slightly influence its function. a. IFA on RON13-KD parasites expressing RON13 phospho-mimetic (ron13pm) or RON13 phospho-null (ron13pn) mutants. Anti-myc antibodies (green) were used to visualize the mutant copy of RON13 and anti-ARO antibodies (magenta) marked the rhoptries. Scale bar = 2µm. Images representative of three biologically independent experiments. **b.** Immunoblot of lysates from RON13-KD parasite complemented with either RON13pm or RON13pn. Anti-myc antibodies were used to visualize the mutant copy of RON13. Image representative of three biologically independent experiments. **c.** Invasion test showing the percentage of intracellular parasites reflecting their ability to invade. One-way ANOVA followed by Tukey's multiple comparison was used to test differences between groups (mean ± SD; n=3 biologically independent experiments). Source data are provided as a Source data file.

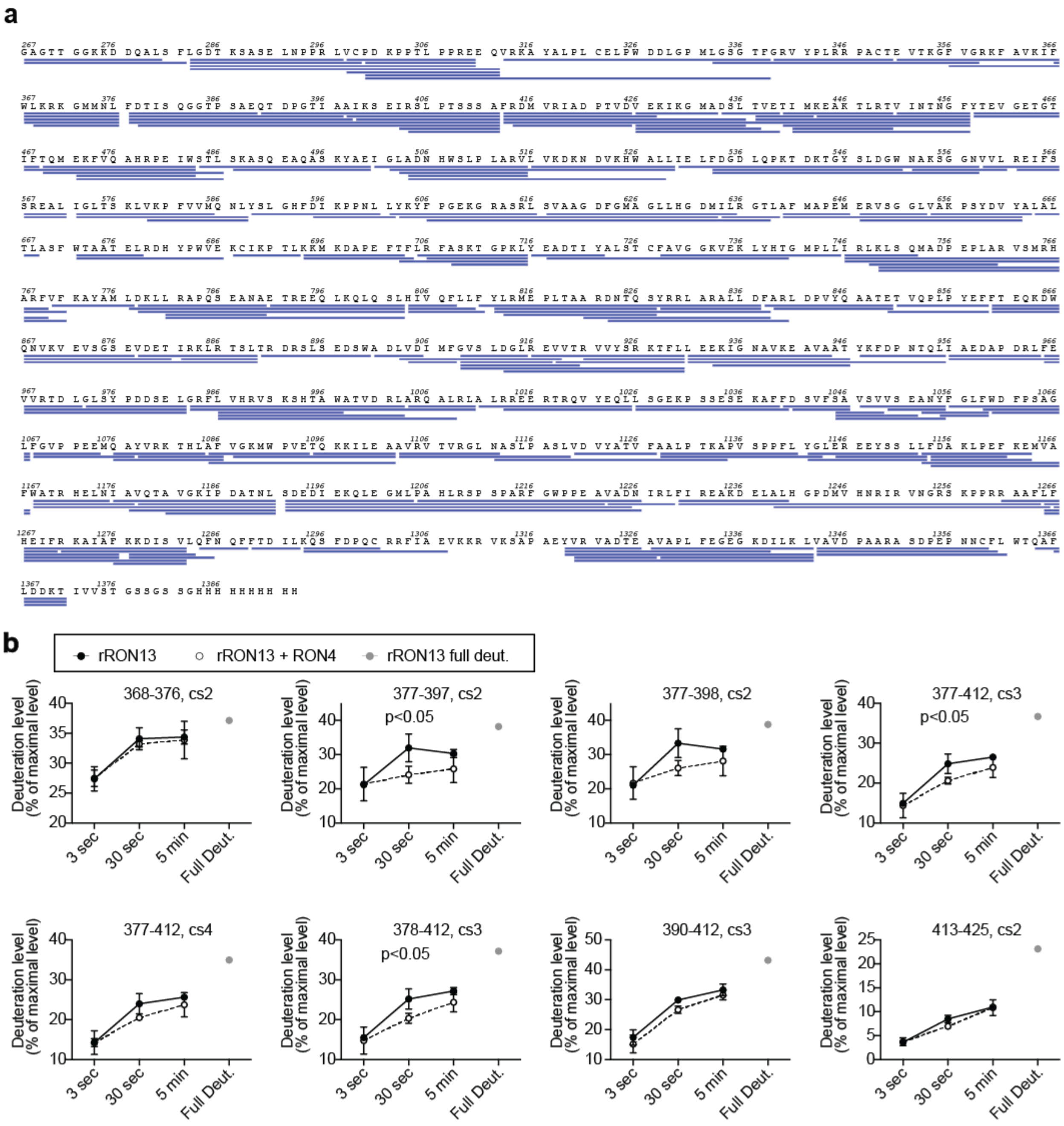

Supplementary Fig. 9. HDX-MS analysis of RON4 interaction with rRON13dk. a. Peptide map showing the peptides selected for HDX-MS analysis **b.** Uptake plots of a selection of peptides used in the HDX-MS analysis. cs: charge state. Indicated p values were determined using a two-tailed paired t-test; n=3 biologically independent experiments. Source data are provided in Supplementary Data 6.

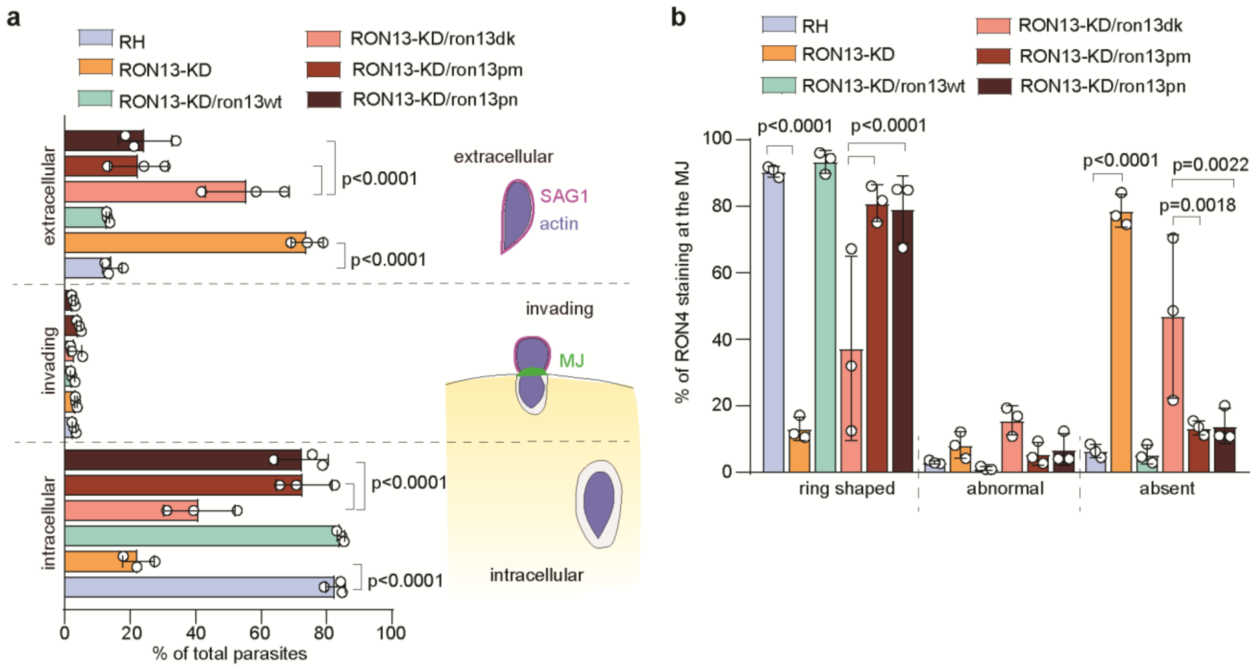

Supplementary Fig. 10. RON13 auto phosphorylation does not impact MJ formation. **a.** Graph representing the proportion of extracellular, invading and intracellular parasites observed in the pulse-invasion assay of RH, RON13-KD, RON13-KD/ron13t, RON13-KD/ron13dk as well as the RON13-KD parasites expressing RON13 phospho-mimetic (ron13pm) or RON13 phospho-null (ron13pn) mutants. A scheme representing the three steps of the invasion processed is depicted. Two-way ANOVA followed by Tukey's multiple comparison were used to test differences between groups in the panel of this figure (mean ± SD; n=3 biologically independent experiments). **b.** Quantification of the different types of RON4 staining (absent, abnormal, ring shaped) observed at the MJ of invading parasites. Two-way ANOVA followed by Tukey's multiple comparison were used to test differences between groups in the panel of this figure (mean ± SD; n=3 biologically independent experiments). Source data are provided as a Source data file.

**Data collection**	
Instrument	FEI Titan Krios / Gatan K2 Summit / Quantum GIF
Magnification	61425 (165kx)
Voltage (kV)	300
Electron exposure (e^-/Å^2)	47
Defocus range (um)	-0.6 to -2.4
Pixel size (Å)	0.8544
Resolution, Å (FSC 0.143)	3.125
Number of particles	320723
**Model refinement**	
FSC 0.5 (masked), Å	3.15
Map sharpening b-factor (Å)	-85.7
Map CC (mask)	0.86
Model composition	
protein residues/ligands	1044 / 0
B factor min / max / mean (Å^2)	19.84 / 110.98 / 49.14
Bond length RMSD (Å)	0.007
Bond angle RMSD (°)	0.756
**Validation**	
MolProbity score	1.76
Clash score	5.20
Rotamer outliers (%)	0.11
Ramachandran plot	
Favored (%)	92.02
Allowed (%)	7.68
Disallowed (%)	0.29

Supplementary Table 1. Cryo-EM data collection, single particle analysis and model building statistics.

Infection 19.02.2020

Challenge 13.05.2020

End of the experiment 19.06.2020

Number of mice infected by each strain n =5 biologically independent animals

		RH		RON13-KD		RON13-KD/ron13wt		RON13-KD/ron13dk	
	days p.i	n sacrifices	Date	n sacrifices	Date	n sacrifices	Date	n sacrifice	Date
	8	1	27.02			1	27.02		
	9	1	28.02			3	28.02		
	10	3	29.02			1	29.02		
Survived	84	0		5		0		5	
challenge	84	n.a		5		n.a		5	
	93			2	22.05				
	94			2	23.05				
end	121			1	19.06			5	19.06
Survived challenge		0		1		0		5	
n seroconverted		n.a		1		n.a		5	

Supplementary Table 2. Virulence of RON13 mutant strains. Table representing the number of CD1 mice infected with the specified parasite strains (top row), their survival and seroconversion to the infection (penultimate and ultimate rows respectively). At day 84 post-infection (p.i) surviving mice were challenged with RH parasites (turquoise row). The blue row indicates the number surviving the challenge infection. Abbreviations: n, number of biologically independent mouse; n.a, not applicable.

Dataset :	RON13	RON13 + RON4	RON13 FD
Description :	RON13	RON13 + RON4	RON13 FD
Reaction volume :	50 ul	50 ul	50 ul
% D2O in the reaction :	81.0%	81.0%	81.0%
temperature :	22 °C	22 °C	22 °C
D2O incubation times (sec) :	3 sec, 30 sec, 5 min	3 sec, 30 sec, 5 min	90 min
Control sample :	Non-deuterated (ND) and fully deuterated (FD) RON13		
Quench buffer :	6 M Urea / 0.1 M NaH2PO4 pH 2.4 / 1% Formic acid		
Quench buffer volume :	20 ul	20 ul	20 ul
Number of peptides analysed :	205	205	205
Sequence coverage :	94.0%	94.0%	94.0%
Replicates :	3, 3, 3	3, 3, 3	1
Standard deviation average (RON13, all time points, %D) :	1.03	1.55	-
Criteria for HDX rate difference :	Difference of HDX level at a given timepoint is > 5 % and > 1 Da and p values of two-tailed paired student t-test is < 0.05		

Supplementary Table 3. HDX-MS experimental details.

			RH/RON4-3Ty			RON13-KD/RON4-3Ty		
**Protein**	Gene ID	MW (kDa)	TSC IP	TSC input	**Enrichment**	TSC IP	TSC input	**Enrichment**
**RON8**	TGGT1_306060	329	66	5	**13.2**	30	4	**7.5**
**RON2**	TGGT1_300100	166	82	16	**5.125**	67	3	**22.33333333**
**RON4**	TGGT1_229010	107	64	20	**3.2**	49	11	**4.454545455**
**RON5**	TGGT1_311470	187	82	26	**3.153846154**	78	25	**3.12**
**AMA1**	TGGT1_255260	63	26	15	**1.733333333**	26	17	**1.529411765**

Supplementary Table 4. The MJ complex is form in absence of RON13. Table depicting the MJ proteins identified by mass spectrometry in the co-immunoprecipitation of RON4 samples from RH and RON13-KD parasites. The table include information regarding the total spectrum count (TCS) in the input (total lysate) and in the IP samples as well as the ratio of enrichment for each of the proteins. Anti-Ty antibodies have been used to immunoprecipitate RON4.

Primer #	Orientation	Sequence 3'->5'	Construct	Strain
6765	F	GATGGGCCCTCACCGACATCCTCAAACAGAG	KI-3TY-DHFR	ASP3-iKD/RON13-3Ty[2]
6766	R	ACGCCTGCAGGGCTCACGACGATTGTTTTATCG	KI-3TY-DHFR	ASP3-iKD/RON13-3Ty[2]
4883	R	AACTTGACATCCCCATTTAC	gRNA 5'ron13 locus	RON13-KD
7012	F	GGAGTTCACAGGTTCTCTTGGTTTTAGAGCTAGAAATA GC	gRNA 5'ron13 locus	RON13-KD
7013	F	CCTCTATGTTTGTCTCTAGCAACGAATCGGCATGTTTG CGGATCCGGGG	PCR cassette TaTi-HXGPRT-TetO7S1	RON13-KD
7015	R	CACGCTTCGTTCGCTTTGCATGCGAGGCAACATTTTGA TATCCCTAGGAATTC	PCR cassette TaTi-HXGPRT-TetO7S1	RON13-KD
P1-7069	F	CTTAGGTCCCTCGTTCCGTAG	Check 5’ integration RON13-KD	-
P4-2903	R	GAGCGAGTTTCCTTGTCGTCAGGCC	Check 5’ integration RON13-KD	-
P3-1935	F	CGCTGCACCACTTCATTATTTCTTCTGG	Check 3’ integration RON13-KD	-
P2-7070	R	CTCTGCATTCAGGTGCCTG	Check 3’ integration RON13-KD	-
8178	F	GTCTTCAGAGGCGCCTTAATGACAAAATGTTGCCTCGC	pRON5-RON13-4myc (part 1)	RON13-KD/ron13wt
7813	R	GCTTGATGCATTTCTCCACCCACGGATAGT	pRON5-RON13-4myc (part 1)	RON13-KD/ron13wt
7814	F	GGTGGAGAAATGCATCAAGCCGACGCTGAAGA	pRON5-RON13-4myc (part 2)	RON13-KD/ron13wt
7815	R	CCAGACGATCCACGGTCGCCCAGGCAGT	pRON5-RON13-4myc (part 2)	RON13-KD/ron13wt
7816	F	GACCGTGGATCGTCTGGCGCGTCAG	pRON5-RON13-4myc (part 3)	RON13-KD/ron13wt
8179	R	GAGATGAGTTTTTGTTCGATGCTCACGACGATTGTTTT ATC	pRON5-RON13-4myc (part 3)	RON13-KD/ron13wt
8548	F	GGGTCACTTTGCGATCAAGCCGC	pRON5-RON13dk-4myc	RON13-KD/ron13dk
8549	R	AAGCTGTAGAGGTTCTGC	pRON5-RON13dk-4myc	RON13-KD/ron13dk
9757	F	CGCTGAACTCGACCCCGTGTACCAG	pRON5-RON13cte(R840E)-4myc	RON13-KD/ron13cte
9026	F	GGGCGCCggtaccACAGGAGGAAAGAAGGATGACCAAG	pFastBac-Melittin-GST-RON13 (271-1371)-His6	Insect cell expression vector
9027	R	CCGAGGAaccggtGCTCACGACGATTGTTTTATCGTCG	pFastBac-Melittin-GST-RON13 (271-1371)-His6	Insect cell expression vector
9595	F	GAgAAtttGTAcTTcCAaGCCGCGCGAGACAACACACAG	pFastBac-RON13 kinase domain-TEV-CTE	Insect cell expression vector
9596	R	tTGgAAgTACaaaTTcTCAGTGAGAGGCTCCATTCGAAGA TAAAAC	pFastBac-RON13 kinase domain-TEV-CTE	Insect cell expression vector
9758	R	GTCGAGTTCAGCGAAGTCGAGTAAAGCGC	pRON5-RON13cte(R840E)-4myc	RON13-KD/ron13cte
9759	F	GTCACGGCAGTCGTCTATTCGAGAAAAACGTTTTTG	pRON5-RON13cte(R921E)-4myc	RON13-KD/ron13cte
9760	R	GACGACTGCCGTGACGACCTCACGCAG	pRON5-RON13cte(R921E)-4myc	RON13-KD/ron13cte
9761	F	GTTCACGAAGTTTCCGCAAGCCACACTGCCTGGGC	pRON5-RON13cte(R989E+K992A)-4myc	RON13-KD/ron13cte

9762	R	TGGCTTGCGGAAACTTCGTGAACCAAAAACCTCCCCAG	pRON5-RON13cte(R989E+K992A)-4myc	RON13-KD/ron13cte
9590	F	GCCATCGCCCAGGGCGGAACAC	pRON5-RON13pn(T379A+S381A)-4myc	RON13-KD/ron13pn
9763	F	ggtggatccggtggcGAgAAtttGTAcTTcCAaGCCGCGC	pFastBac-RON13 kinase domain-linker-TEV-linker-CTE	Insect cell expression vector
9764	R	gccaccggatccaccAGTGAGAGGCTCCATTCGAAGATAAAAC	pFastBac-RON13 kinase domain-linker-TEV-linker-CTE	Insect cell expression vector
9765	F	ggaagcggtggatccGCCGCGCGAGACAACACACAG	pFastBac-RON13 kinase domain-linker-TEV-linker-CTE	Insect cell expression vector
9766	R	ggatccaccgcttcctTGgAAgTACaaaTTcTCgccaccg	pFastBac-RON13 kinase domain-linker-TEV-linker-CTE	Insect cell expression vector
9591	R	GGCGATGGCGTCGAACAGATTCATC	pRON5-RON13pn(T379A+S381A)-4myc	RON13-KD/ron13pn
9946	F	GCCAGAGTTCGCCTTCCTGCGGT	pRON5-RON13pn(T379A+S381A+T703A)-4myc	RON13-KD/ron13pn
9947	R	GCGTCCTTCATCTTCTTCAGCG	pRON5- RON13pn(T379A+S381A+T703A)-4Myc<br>pRON5- RON13pm(T379D+S381D+T703D)-4Myc	RON13-KD/ron13pn<br>RON13-KD/ron13pm
9806	F	GATATCGACCAGGGCGGAACAC	pRON5-RON13pm(T379D+S381D)-4myc	RON13-KD/ron13pm
9807	R	GTCGATATCGTCGAACAGATTCAT	pRON5-RON13pm(T379D+S381D)-4myc	RON13-KD/ron13pm
9948	F	GCCAGAGTTCGACTTCCTGCGGT	pRON5- RON13pm(T379D+S381D+T703D)-4Myc	RON13-KD/ron13pm
8263	F	GCAAGGTTTCGTGCTGATCAGCGCTTCTTATCGTAGGG	pRON5-RON13-4myc-BLA	RON13-BLA
8264	R	ACCAGCGTTTCTGGGTGGGCCAGATCTTCTTCAGAAATAAGTTTTTG	pRON5-RON13-4myc-BLA	RON13-BLA
8798	F	TACTTCCAATCCAATTTAATGCCCTGTCTCAGAGATGATTACGAC	LIC-ARO-YFP	ARO-YFP
8799	R	TCCTCCACTTCCAATTTTAGCCTCCGACAGCCGGACCAAGA	LIC-ARO-YFP	ARO-YFP
8800	F	TACTTCCAATCCAATTTAATGCTTGTGGAAGCAGAGAATTTGAGG	LIC-RON11-YFP	RON11-YFP
8801	R	TCCTCCACTTCCAATTTTAGCCACTCGCTCTCCAGGAATTGG	LIC-RON11-YFP	RON11-YFP
8802	F	TACTTCCAATCCAATTTAATGCTTTCGTCCTTTTCTTGGAGAAGATCCG	LIC-RON13-YFP (part 1)	RON13-YFP
8803	R	GAAGGATATCGAATGCTTTCATGAAAAC	LIC-RON13-YFP (part 1)	RON13-YFP
8804	F	CATTCGATATCCTTCGATTGCATGCAGT	LIC-RON13-YFP (part 2)	RON13-YFP
8805	R	TCCTCCACTTCCAATTTTAGCGCTCACGACGATTGTTTT	LIC-RON13-YFP (part 2)	RON13-YFP
10019	F	GGATGAACTTCGACAAACTCGTTTTAGAGCTAGAAATAGC	gRNA RON4 frame shift	RON4 KO
10116	F	gtaaatggggatgtcaagttGCAAAGTGGAGAGCATCATTGCgttttagagctagaaatagc	gRNA RON4 locus editing RON4pn and RON4pm	RON4pn and RON4pm

10117	R	gctatttctagctctaaaacGAGTTTGTCGAAGTTCATCCaacttgacatc cccattta	gRNA RON4 locus editing RON4pn and RON4pm	RON4pn and RON4pm
10094	F	gctctcagGGATGTATCCGAACATG	Sequencing RON4 frameshift	RON4 KO
10097	R	GCAACCGGTTCCTCAGCACT	Sequencing RON4 frameshift	RON4 KO

Supplementary Table 5. List of oligonucleotide primers and constructs used in this study.

		Dilution	
Antibody	Reference	IFA	WB
RON2 (rb)	[3] kind gift from Dr. M. Lebrun	1/250 (U-ExM); 1/500 (IFA)	1/1000
RON4 (rb)	[3] kind gift from Dr. M. Lebrun	1/1500 (U-ExM); 1/3000 (IFA)	1/1000
RON4 (ms) hybridoma T5 4H1	[4] kind gift from Dr. JF. Dubremetz	1/10	-
RON5C (ms)	[5] kind gift from Dr. P. Bradley	1/1000	1/1000
RON8 (rat)	[5] kind gift from Dr. M. Lebrun	1/400	-
RON9 (ms) hybridoma	[4] kind gift from Dr. JF. Dubremetz	1/2.5 (U-ExM); 1/10 (IFA)	-
RON13 (rb)	this study	1/1000	1/500
ROP2/3/4 (ms) hybridoma	[6] kind gift from Dr. JF. Dubremetz	1/2.5 (U-ExM)	
ROP1 (ms) hybridoma	kind gift from Dr. JF. Dubremetz	1/10	-
ARO (rb)	[7]	1/1000	-
GAP45 (rb)	[8]	1/10000	-
SAG1 (ms) hybridoma T4 1E5	[9]	1/10	-
SAG1 (ascite)	[10]	1/3000	-
Actin (ms) hybridoma	[11]	1/10	1/10
Catalase (rb)	[12]	-	1/2000
α + β tubulin (gp)	[13]	1/250 (U-ExM)	
Phospho-STAT6 (rb)	Cell signalling (9361)	1/400	-
Myc (ms) hybridoma 9E10	from DSHB by Bishop, J.M. (DSHB Hybridoma Product 9E 10)	1/10	1/10
Myc (rb)	Sigma (3956)	1/1000	-
Ty (ms) hybridoma BB2	[14]	1/2.5 (U-ExM); 1/10 (IFA)	1/10
Ty (rb)	kind gift from Dr. CJ. Tonkin[15]	1/1000 (U-ExM)	-
GFP (ms)	Roche	1/2000	1/1000
MIC2 (ms)	kind gift from Dr. V. Carruthers	-	1/10
GRA1 (rb)	Anawa	-	1/3000
HA (rat)	Roche (clone 3F10)	1/1000	
HA (ms) hybridoma	Clone 12CA5		1/10

Supplementary Table 6. List of antibodies used in this study. The species in which the antibodies were produced are mentioned between parentheses. Rabbit (rb); mouse (ms) ; guinea pig (gp).

Supplementary references

1. Barylyuk, K. *et al.* A Comprehensive Subcellular Atlas of the Toxoplasma Proteome via hyperLOPIT Provides Spatial Context for Protein Functions. *Cell Host Microbe* **28**, 752-766.e9 (2020).
2. Dogga, S. K. *et al.* A druggable secretory protein maturase of Toxoplasma essential for invasion and egress. *Elife* **6**, 1–35 (2017).
3. Besteiro, S., Dubremetz, J. F. & Lebrun, M. The moving junction of apicomplexan parasites: A key structure for invasion. *Cell. Microbiol.* **13**, 797–805 (2011).
4. Leriche, M. A. & Dubremetz, J. F. Characterization of the protein contents of rhoptries and dense granules of Toxoplasma gondii tachyzoites by subcellular fractionation and monoclonal antibodies. *Mol. Biochem. Parasitol.* **45**, 249–259 (1991).
5. Straub, K. W., Cheng, S. J., Sohn, C. S. & Bradley, P. J. Novel components of the Apicomplexan moving junction reveal conserved and coccidia-restricted elements. *cell Microbiol.* **11**, 590–603 (2009).
6. Sadak, A., Taghy, Z., Fortier, B. & Dubremetz, J. Characterization of a family of rhoptry proteins of Toxoplasma gondii. *Mol. Biochem. Parasitol.* **29**, 203–211 (1988).
7. Mueller, C. *et al.* The toxoplasma protein ARO mediates the apical positioning of rhoptry organelles, a prerequisite for host cell invasion. *Cell Host Microbe* **13**, 289–301 (2013).
8. Frenal, K., Polonais, V., Marq, J., Stratmann, R. & Limenitakis, J. Functional Dissection of the Apicomplexan Glideosome Molecular Architecture. *Cell Host Microbe* 343–357 (2010) doi:10.1016/j.chom.2010.09.002.
9. Couvreur, G., Sadak, A., Fortier, B. & Dubremetz, J. F. Surface antigens of Toxoplasma gondii. *Parasites and Vectors* **97**, 1–10 (1988).
10. Kim, K., Soldati, D. & Boothroyd, J. Gene replacement in Toxoplasma gondii with chloramphenicol acetyltransferase as selectable marker. *Science (80-.).* **262**, 911–914 (1993).
11. Herm-gotz, A. *et al.* Toxoplasma gondii myosin A and its light chain : a fast , single-headed , plus-end-directed motor. *EMBO J.* **21**, 2149–2158 (2002).
12. Ding, M., Clayton, C., Soldati, D., Biologie, M. & Feld, I. N. Toxoplasma gondii catalase : are there peroxisomes in Toxoplasma ? *J. Cell Sci.* **2419**, 2409–2419 (2000).
13. Tosetti, N. *et al.* Essential function of the alveolin network in the subpellicular microtubules and conoid assembly in Toxoplasma gondii. *Elife* **9**, 1–22 (2020).
14. Bastin, P., Bagherzadeh, A., Matthews, K. R. & Gull, K. A novel epitope tag system to study protein targeting and organelle biogenesis in Trypanosoma brucei. *Mol. Biochem. Parasitol.* **77**, (1996).
15. Uboldi, A. D. *et al.* Regulation of Starch Stores by a Ca2+-Dependent Protein Kinase Is Essential for Viable Cyst Development in Toxoplasma gondii. *Cell Host Microbe* **18**, 670–681 (2015).
