# Supplementary material for: Structural insights into an atypical secretory pathway kinase crucial for Toxoplasma gondii invasion
Source: Nat Commun. 2021 Jun 18;12:3788. doi: 10.1038/s41467-021-24083-y (PMC8213820; doi:10.1038/s41467-021-24083-y)
Supplement: Supplementary file 2 — Description of Additional Supplementary Files [file 41467_2021_24083_MOESM2_ESM.docx]

Description of Additional Supplementary Files for

**Structural insights into an atypical secretory pathway kinase crucial for Toxoplasma gondii invasion**

Gaëlle Lentini1†, Rouaa Ben Chaabene1†, Oscar Vadas1†, Chandra Ramakrishnan2, Budhaditya Mukherjee1,3, Ved Mehta4, Matteo Lunghi1, Jonas Grossmann5,6, Bohumil Maco1, Rémy Visentin1, Adrian B. Hehl2, Volodymyr M. Korkhov4,7,* and Dominique Soldati-Favre1,*

Correspondence to: dominique.soldati-favre@unige.ch, volodymyr.korkhov@psi.ch

†These authors contributed equally to the work

**Supplementary Data and Movies legends**

File Name: Supplementary Data 1

Description: RON13 possesses the canonical motives of active kinases. Sequence alignment of TgRON13 kinase domain with other kinase domains of representative eukaryotic kinases Homo sapiens PKA (HsPKA), HsGSK3b, HsCAMK1, HsSrc, HsCK2A1, HsCDK2 and TgPKA and TgAGC as well as other members of the ROPK family. ROP16 and ROP18 are active rhoptry kinases. ROP7, ROP8 and ROP5 are pseudo-kinases. The subdomains of the kinase domain are indicated and the secondary structure are depicted at the top row of each line of the alignment. The N-lobe insertion of TgRON13 is indicated in blue and conserved residues are shown in red.

File Name: Supplementary Data 2

Description: RON13 is highly conserved among coccidian parasites. Sequence alignment of TgRON13 full-length with its orthologs among the coccidian parasites Eimeria tenella (Eth), Sarcocystis neurona (Sn), Besnoitia besboiti (Besb), Neospora caninum (NcLiv), Hammondia hammondi (Hha), Cyclospora cayetanensis (cyc) and Cystoisospora suis (csui). Conserved residues are shown in red. The N-lobe and C-lobe of the kinase domain and the C-terminal extension are mentioned. The N-lobe insertion of TgRON13 is indicated in blue. The secondary structure are depicted at the top row of each line of the alignment.

File Name: Supplementary Data 3

Description: Phosphoproteome analysis identifies new phosphorylation sites in T. gondii. Tables listing phosphosites and the corresponding phosphoproteins previously reported to be phosphorylated15 as well as the new phosphosites and the corresponding phosphoproteins identified in this study.

File Name: Supplementary Data 4

Description: Quantitative data of T. gondii phosphoproteome analysis and pairing. Quantitative data of T. gondii phosphopeptides differentially phosphorylated (log2 FC>2) and the corresponding proteins between RH vs RON13-KD (Dataset 1) and RON13-KD/ron13wt vs RON13-KD/ron13dk (Dataset 2). Peptides/proteins present in both Datasets are listed in the common peptides/proteins sheets. The modified aminoacids and their position are mentioned in column G. Proteins for which several phosphopeptides have been found are in red. RON13 (TGGT1_321650) is highlight in red. A color code has been assigned to each entry according to the predicted localization of the corresponding proteins 16. The MCMC probability of localization is mentioned between brackets in column C. PEP = posterior error probability.

File Name: Supplementary Data 5

Description: List of RON13 rhoptry substrates. Table listing the rhoptry protein substrates of RON13. Proteins have been extracted from Supplementary Data 4 according to their predicted or known localization in the rhoptry or if they possess a ‘ROP-like’ transcriptomic profile 17. Their previous identification as ASP3 substrates 1 and the presence of a signal peptide (SP) or transmembrane domain (TMD) are mentioned. A color gradient is assigned for the reported fitness score^15^.

File Name: Supplementary Data 6

Description: HDX-MS table, peptide characterization. Table listing all peptides used for the HDX-MS analysis of rRON13 protein, showing deuterium incorporation levels at each of the timepoint and including standard-deviation values. Differences in deuterium incorporation between the two conditions, that is rRON13 alone and rRON13 in the presence of RON4, are shown on the right for each of the selected peptide.

File Name: Supplementary Data 7

Description: Sequence alignment of TgRON13 with PF3D7_1321100, a putative rhoptry kinase in Plasmodium falciparum. Sequence alignment of PF3D7_1321100 with TgRON13 and its orthologs among the coccidian parasites Eimeria tenella (Eth), Sarcocystis neurona (Sn), Besnoitia besboiti (Besb), Neospora caninum (NcLiv), Hammondia hammondi (Hha), Cyclospora cayetanensis (cyc) and Cystoisospora suis (csui). Conserved residues are shown in red. The different subdomains of the kinase domain and the C-terminal extension are mentioned. The secondary structure are depicted at the top row of each line of the alignment.

File Name: Supplementary Data 8

Description: Curated alignment used to generate the phylogenetic tree of eukaryotic kinases.

File Name: Supplementary Movie 1

Description: Rhoptry morphology of control RH sample. Part of FIBSEM image volume of the control RH sample showing two tachyzoites. Rhoptry organelles are highlighted in green and show characteristic appearance of elongated bulbs and long thin electron dense necks. Segmentation of rhoptries from bottom tachyzoite cell was used to create 3D model displayed in Figure 1b. Scale bar: 1 µm.

File Name: Supplementary Movie 2

Description: Rhoptry morphology in ASP3 depleted parasites. Part of FIBSEM image volume of the ASP3-iKD parasites treated with ATc for 48 h sample showing two tachyzoites. Rhoptry organelles are highlighted in green and show significantly altered rhoptry morphology, in particular shortening and “swelling” of electron dense neck compared to the control. Segmentation of rhoptries from bottom tachyzoite cell was used to create 3D model displayed in Figure 1b. Scale bar: 1 µm.

File Name: Supplementary Movie 3

Description: The atypical structure of RON13 kinase. Cryo-EM density map determined by single particle analysis at a resolution of 3.1Å and the corresponding views of the atomic model of rRON13dk. The kinase domain (orange tones), the N-lobe insertion (NLI, red) and the C-terminal extension (CTE, grey) are depicted. The position of the ATP binding site is indicated by the ATP ligand modeled into the active site (absent in the experimentally determined density map).
