# Supplementary material for: Structural insights into an atypical secretory pathway kinase crucial for Toxoplasma gondii invasion
Source: Nat Commun. 2021 Jun 18;12:3788. doi: 10.1038/s41467-021-24083-y (PMC8213820; doi:10.1038/s41467-021-24083-y)
Supplement: Supplementary file 3 — Supplementary Data 1 [file 41467_2021_24083_MOESM3_ESM.pdf]

```
HsPKA/1-351

HsPKA/1-351              .....................................................................................
HsGSK3b/1-420            .....................................................................................
HsCAMK1/1-370            .....................................................................................
HsSrc/1-536          1   ...........................................................................MGSNKSKPKD
HsCK2A1/1-391            .....................................................................................
HsCDK2/1-298             .....................................................................................
TgPKAc/1-333             .....................................................................................
TgAGC/1-514          1   ...........................................................................MP......LR
TgROP5/1-461             .....................................................................................
TgROP7/1-575         1   ...................................MG.......HPTSF...GQPSCLV........WLAAAFLVLGLCLVQQ..
TgROP8/1-575         1   ........................MFS..VLRNRNMR.......HGPSI...GSSSCLI........WLAAAFFVSALGHVQQ..
TgROP16/1-707        1   ............................................................MKVTTKGLAFALALLFCTRCATA..
TgROP18/1-554            .....................................................................................
TgRON13/1-1375       1   MLPRMQSERSVARQSLGATRSRCLLRFATKKWASRGLGSSNRPGRPPPSSAAGHRASCPFFPRSSFSASFALRFLCPVLCSLLLA
TgRON13/1-1375

HsPKA/1-351

HsPKA/1-351              .....................................................................................
HsGSK3b/1-420            .....................................................................................
HsCAMK1/1-370            .....................................................................................
HsSrc/1-536         11   AS...............................QR..RRS..LEPAEN......VHGAGGGAFPASQT.................
HsCK2A1/1-391            .....................................................................................
HsCDK2/1-298             .....................................................................................
TgPKAc/1-333             .....................................................................................
TgAGC/1-514          5   T......................MSVTSGQYPVQPHQRRPLF..GRGNLFFFLNSGSSDGAAVG....TPGVSP.SRE.....QS
TgROP5/1-461             .....................................................................................
TgROP7/1-575             .....................................................................................
TgROP8/1-575             .....................................................................................
TgROP16/1-707       24   ..RYMSFEEAQ...................KASEAAK.RQIATLPSPDS.TLSNPGSKHRNRGGSPAAGQPSQSTLQPEQAAAEV
TgROP18/1-554            .....................................................................................
TgRON13/1-1375      86   TLFFVSPREVDALKFRGNGGRHLNAELDGNWAKRFQ...ETQRAAPPDSFFVSDSEISGRTSLLSLASSEQGTSTVDSR....FV
TgRON13/1-1375

HsPKA/1-351

HsPKA/1-351              .....................................................................................
HsGSK3b/1-420            .....................................................................................
HsCAMK1/1-370            .....................................................................................
HsSrc/1-536         38   .....................PSKPASADGHRGPSAAFAPAAAEPKLFGGFNSSDTVTSPQRAGPLAGGVTTFVALYDYESRTET
HsCK2A1/1-391            .....................................................................................
HsCDK2/1-298             .....................................................................................
TgPKAc/1-333             .....................................................................................
TgAGC/1-514         56   GFQMLTKRSLFGVSHPPTHS..SRTVAQ..MAPRSQAYTPQPH.....................R...............SSGDC
TgROP5/1-461             .....................................................................................
TgROP7/1-575        31   GAGIQRP.HQWKSSEAALSVSPA.....................G........DIV....................DKYSHDSTE
TgROP8/1-575        40   GAGVVRP.RHWQNSEAAVSVRSP.....................G........GAS....................PRHFHSPIE
TgROP16/1-707       86   GLGAG.....GSTQGQ.....GRTGGSAGAREERRSPSPQSAY.....................P...............ATSSA
TgROP18/1-554        1   MFSVQRP........PLTRTVVRMG..............................LATLLPKTAC...........LAGLNVALV
TgRON13/1-1375     164   GLSGQKPRHIWGSQEETGERREDRQEESEGRRGNEDAYHPHTF.....................S...............H.SSS
TgRON13/1-1375

HsPKA/1-351
                                                                                   .
HsPKA/1-351          1   .........................................................................MG...NAA....
HsGSK3b/1-420        1   ..........................MSGRPRTTSFA.....................ESCKPV....QQPSAFG...S......
HsCAMK1/1-370            .....................................................................................
HsSrc/1-536        102   DLSFKK....GERLQIVNNTEGDWWLAHSLSTGQTGY.....................I...PSNYV.............APSDS
HsCK2A1/1-391        1   ..........................MSGPVP....................................SRARVYT...D......
HsCDK2/1-298             .....................................................................................
TgPKAc/1-333             .....................................................................................
TgAGC/1-514        101   ST.......................AEK..............................ASCPTGSAAVHQATNMG...SVSAVAG
TgROP5/1-461         1   ...........................MSG............................GRVPRVPA.SSTTT......SASEGIF
TgROP7/1-575        66   GENTVS...EG..EAEGSRGGSWLEQEGV.............................ELRPPLLDSQTGTS......TASPTGF
TgROP8/1-575        75   PVAFID...GE..HDEDKHEGSWLEQEAA.............................EDVTPLPGSHTEAQ......TQSPSAF
TgROP16/1-707      125   SLRGYQTQLSPSHLPPRSSGPGGWFPTESIFTPWS.........................SPPQPLTQRKP.SLS...GVVVTEF
TgROP18/1-554       37   FLLFQV.....QDGTGITLGPSKLDSKPTSLD..SQQHVADKRWLATVGHYKHLAGATESTRDVSLLEERAQHRVNAQETNQRRT
TgRON13/1-1375     212   SMPFY................GALFDSSSAFPPFSAA.....................VSSPPFSMTVSPADRVI...QALPIAF
TgRON13/1-1375

HsPKA/1-351

HsPKA/1-351          6   ..................................AAK................................................
HsGSK3b/1-420       26   ....................M.............K..................................................
HsCAMK1/1-370            .....................................................................................
HsSrc/1-536        146   I.....QAEEWYFGKITRRESERLLLNAENP...RGT..............FLVRES............................
HsCK2A1/1-391       15   ....................V.............N..................................................
HsCDK2/1-298             .....................................................................................
TgPKAc/1-333             .....................................................................................
TgAGC/1-514        130   P......LGRMWGKLSSMSTM.............ATS................................................
TgROP5/1-461        24   .R.RLVRRLRR.GRGTA.................DGAGV...ADETHQEPRPPLRKRL...........................
TgROP7/1-575       111   .R.RFLRHLRFWRRGST.................RGSDD...AAEVSRRTRVPLHTRL...........................
TgROP8/1-575       120   .R.RLTRRLRFWRRGVT.................GGSDA...GEEAPQTPRPPLRTRI...........................
TgROP16/1-707      181   Q.....EPQEQYGAASSLASSPKRYVSGASSSALSGKAVPTPASLGQENPLFPVQSATLDSGIQSPAQERRGSPQRQIAMSTENP
TgROP18/1-554      115   IFQRLLNLLRRRE.........................RD..GEVSGSAADSSSRPRLS..........................
TgRON13/1-1375     257   A.....EHK.SGGEKASREET.............GGK................................................
TgRON13/1-1375
```

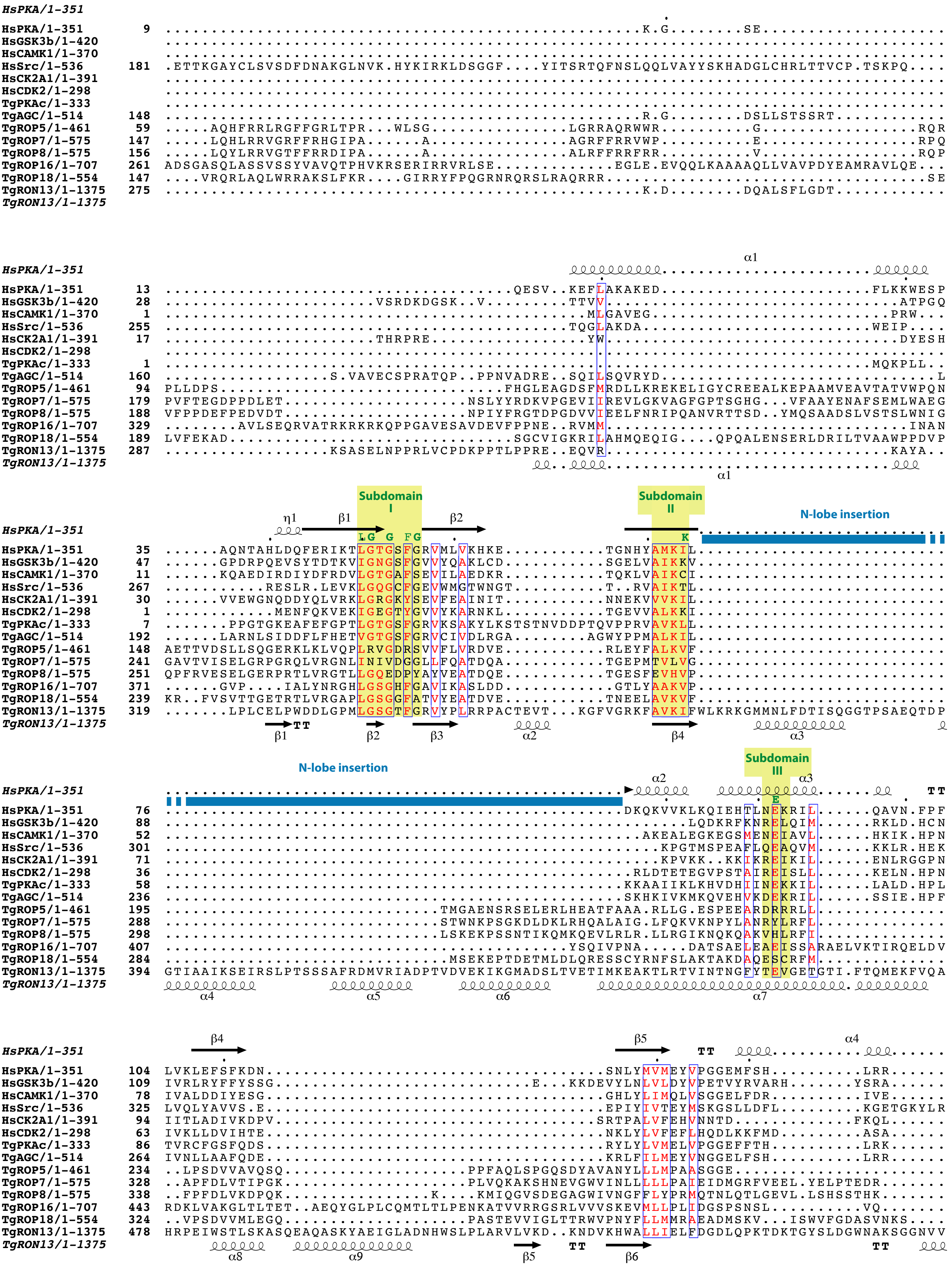
HsPKA/1-351
HsPKA/1-351 9 ....K.G.......SE..................
HsGSK3b/1-420 ....
HsCAMK1/1-370 ....
HsSrc/1-536 181 .ETTKGAYCLSVSDFDNAKGLNVK.HYKIRKLDSGGF....YITSRTQFNSLQQLVAYYSKHADGLCHRLTTVCP.TSKPQ....
HsCK2A1/1-391 ....
HsCDK2/1-298 ....
TgPKAc/1-333 ....
TgAGC/1-514 148 ....R.G.......DSLLSTSSRT..........
TgROP5/1-461 59 .....AQHFRRLRGFFGRLTPR...WLSG..............LGRRAQRWWR.........G.................RQR
TgROP7/1-575 147 .....LQHLRRVGRFFRHGIPA......A..............AGRFFRRVWP.........E.................RPQ
TgROP8/1-575 156 .....LQYLRRVGTFFRRDIPA......A..............ALRFFRRFRR.........V.................RQP
TgROP16/1-707 261 ADSGASQLASSVSSYVAVQTPHVKRSERIRRVRLSE.............EGLE.EVQQLKAAAAQLLVAVPDYEAMRAVLQE...
TgROP18/1-554 147 ....VRQRLAQLWRRAKSLFKR....GIRRYFPQGRNRQRSLRAQRRR..............................SE
TgRON13/1-1375 275 ....K.D.......DQALSFLGDT..........
TgRON13/1-1375
α1
HsPKA/1-351
HsPKA/1-351 13 ..............................QESV..KEFLAKAKED.......................FLKKWESP
HsGSK3b/1-420 28 ..................VSRDKDGSK..V.........TTVV.........................ATPGQ
HsCAMK1/1-370 1 ....MLGAVEG.....................PRW...
HsSrc/1-536 255 ....TQGLAKDA....................WEIP....
HsCK2A1/1-391 17 ..................THRPRE.................YW...........................DYESH
HsCDK2/1-298 ....
TgPKAc/1-333 1 ....MQKPLL..
TgAGC/1-514 160 .................S.VAVECSPRATQP..PPNVADRE..SQILSQVRYD..........................L
TgROP5/1-461 94 PLLDPS....................FHGLEAGDSFMRDLLKREKELIGYCREEALKEPAAMVEAVTATVWPQN
TgROP7/1-575 179 PVFTEGDPPDLET.................NSLYYRDKVPGEVIIREVLGKVAGFGPTSGHG...VFAAYENAFSEMLWAEG
TgROP8/1-575 188 VFPPDEFPEDVDT.................NPIYFRGTDPGDVVIEELFNRIPQANVRTTSD..YMQSAADSLVSTSLWNIG
TgROP16/1-707 329 ........AVLSEQRVATRKRKRKQPPGAVESAVDEVFPPNE..RVMM.........................INAN
TgROP18/1-554 189 LVFEKAD.......................SGCVIGKRILAHMQEQIG....QPQALENSERLDRILTVAAWPPDVP
TgRON13/1-1375 287 .................KSASELNPPRLVCPDKPPTLPPRE..EQVR.........................KAYA..
TgRON13/1-1375
α1
Subdomain I
Subdomain II
N-lobe insertion
η1
β1
β2
L G G FG
K
HsPKA/1-351
HsPKA/1-351 35 ......AQNTAHLDQFERIKTLGTGSFGRVMLVKHKE..........TGNHYAMKIL...............................
HsGSK3b/1-420 47 ......GPDRPQEVSYTDTKVIGNGSFGVVYQAKLCD..........SGELVAIKKV...............................
HsCAMK1/1-370 11 ......KQAEDIRDIYDFRDVLGTGAFSEVILAEDKR..........TQKLVAIKCI...............................
HsSrc/1-536 267 ..........RESLR.LEVKLGQGCFGEVWMGTWNGT.........T..RVAIKTL...............................
HsCK2A1/1-391 30 ......VVEWGNQDDYQLVRKLGRGKYSEVFEAINIT..........NNEKVVVKIL...............................
HsCDK2/1-298 1 ..........MENFQKVEKIGEGTYGVVYKARNKL..........TGEVVALKKI...............................
TgPKAc/1-333 7 ......PPGTGKEAFEFGPTLGTGSFGRVKSAKYLKSTSTNVDDPTQVPPRVAVKLL...............................
TgAGC/1-514 192 ......LARNLSIDDFLFHETVGTGSFGRVCIVDLRGA.......AGWYPPMALKIL...............................
TgROP5/1-461 148 AETTVDSLLSQGERKLKLVQPLRVGDRSVVFLVRDVE..........RLEYFALKVF...............................
TgROP7/1-575 241 GAVTVISELGRPGRQLVRGNLINIVDGGLLFQATDQA..........TGEPMTVLVG...............................
TgROP8/1-575 251 QPFRVESELGERPRTLVRGTLLGQEDPYAYVEATDQE..........TGESFEVHVP...............................
TgROP16/1-707 371 ......GVP....IALYNRGHLGSGHFGAVIKASLDD..........GTLYAAKVP...............................
TgROP18/1-554 239 KR..FVSVTTGETRTLVRGAPLGSGGFATVYEATDVE..........TNEELAVKVF...............................
TgRON13/1-1375 319 ......LPLCELPWDDLGPMLGSGTFGRVYPLRRPACTEVT...KGFVGRKFAVKIFWLKRKGMMNLFDTISQGGTPSAEQTDP
TgRON13/1-1375
TT
β1
β2
β3
α2
β4
α3
N-lobe insertion
Subdomain III
α2
α3
E
TT
HsPKA/1-351
HsPKA/1-351 76 ....DKQKVVKLKQIEHTLNEKRIL......QAVN.FPF
HsGSK3b/1-420 88 ....LQDKRFKNRELQIM......RKLD.HCN
HsCAMK1/1-370 52 ....AKEALEGKEGSMENEIAVL......HKIK.HPN
HsSrc/1-536 301 ....KPGTMSPEAFLQEAQVM......KKLR.HEK
HsCK2A1/1-391 71 ....KPVKK..KKIKREIKIL......ENLRGGPN
HsCDK2/1-298 36 ....RLDTETEGVPSTAIREISLL......KELN.HPN
TgPKAc/1-333 58 ....KKAAIIKLKHVDHIINEKKIL......LALD.HPL
TgAGC/1-514 236 ....SKHKIVKMKQVEHVKDEKRIL......SSIE.HPF
TgROP5/1-461 195 ....TMGAENSRSELERLHEATFAAA.RLLG.ESPEEARDRRRLL............
TgROP7/1-575 288 ....STWNKPSGKDLDKLRHQALAIG.LFQKVKNPYLANRYLRFL............
TgROP8/1-575 298 ....LSKEKPSSNTIKQMKQEVLRLR.LLRGIKNQKQAKVHLRFI............
TgROP16/1-707 407 ....YSQIVPNA.....DATSAELEAEISSARAELVKTIRQELDV
TgROP18/1-554 284 ....MSEKEPTDETMLDLQRESSCYRNFSLAKTAKDAQESCRFM............
TgRON13/1-1375 394 GTIAAIKSEIRSLPTSSSAFRDMVRIADPTVDVEKIKGMADSLTVETIMKEAKTLRTVINTNGFYTEVGETGTI.FTQMEKFVQA
TgRON13/1-1375
α4
α5
α6
α7
β4
β5
TT
α4
HsPKA/1-351
HsPKA/1-351 104 LVKLEFSFKDN...............................SNLYMVMEYVPGGEMFSH..........LRR......
HsGSK3b/1-420 109 IVRLRYFFYSSG.......................E...KKDEVYLNLVLDYVPETVYRVARH.......YSRA......
HsCAMK1/1-370 78 IVALDDIYESG...............................GHLYLIMQLVSGGELFDR..........IVE......
HsSrc/1-536 325 LVQLYAVVS.E...............................EPIYIVTEYMSKGSLLDFL..........KGETGKYLR
HsCK2A1/1-391 94 IITLADIVKDPV..............................SRTPALVFEHVNNTD..............FKQL.....
HsCDK2/1-298 63 IVKLLDVIHTE...............................NKLYLVFEFLHQDLKKFMD.........ASA.....
TgPKAc/1-333 86 TVRCFGSFQDS...............................RYLYLVMELVPGGEFFTH..........LRK.....
TgAGC/1-514 264 IVNLLAAFQDE...............................KRLFILMEYVNGGELFSH..........LRR.....
TgROP5/1-461 234 ..LPSDVVAVQSQ...............PPFAQLSPGQSDYAVANYLLLMPAASGGE.........................
TgROP7/1-575 328 ..APFDLVTIPGK...............PLVQKAKSHNEVGWVINLLLLPAIEIDMGRFVEEL.YELPTEDR.......
TgROP8/1-575 338 ..FPFDLVKDPQK...........K....KMIQGVSDEGAGWIVNGFFLYPRMQTNLQTLGEVL.LSHSSTHK.......
TgROP16/1-707 443 RDKLVAKGLTLTET..AEQYGLPLCQMTLTLPENKATVVRRGSRLVVVSKEVMLLPLIDGSPSNSL..........VQ.....
TgROP18/1-554 324 ..VPSDVVMLEGQ...............PASTEVVIGLTTRWVPNYFLLMRAEADMSKV...ISWVFGDASVNKS....
TgRON13/1-1375 478 HRPEIWSTLSKASQEAQASKYAEIGLADNHWSLPLARVLVKD..KNDVKHWALLIELFDGDLQPKTDKTGYSLDGWNAKSGGNVV
TgRON13/1-1375
α8
α9
β5
TT
β6
TT

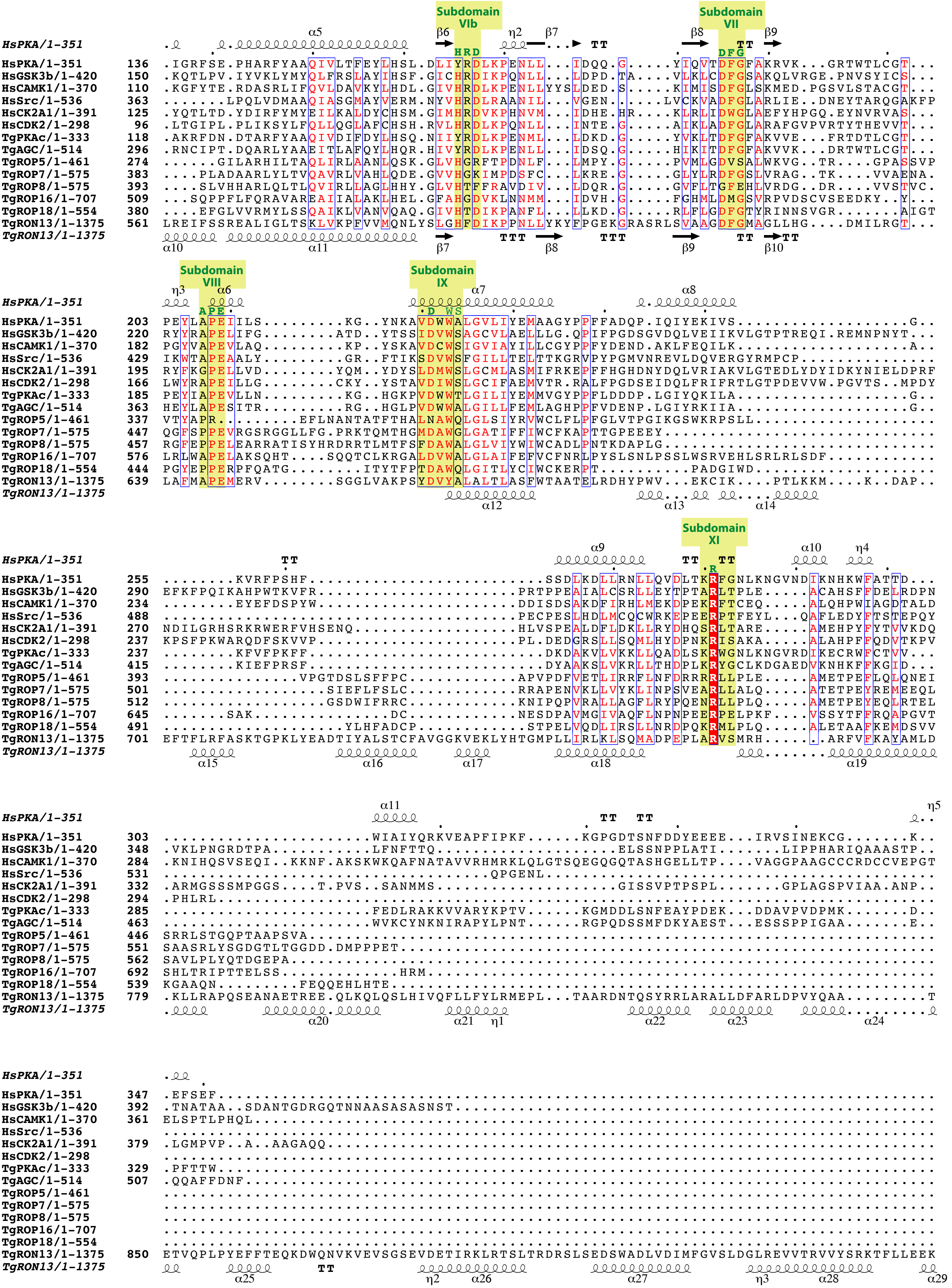

Subdomain VIb
Subdomain VII
HRD
DFG
HsPKA/1-351 136 .IGRFSE.PHARFYAAQIVLTFEYLHSL.DLIYRDLKPENLL...IDQQ.G.....YIQVTDFGFAKRVK...GRTWTLCGT...
HsGSK3b/1-420 150 .KQTLPV.IYVKLYMYQLFRSLAYIHSF.GICHRDIKPQNLL...LDPD.TA....VLKLCDFGSAKQLVRGE.PNVSYICS...
HsCAMK1/1-370 110 .KGFYTE.RDASRLIFQVLDAVKYLHDL.GIVHRDLKPENLLYYSLDED.S.....KIMISDFGLSKMED.PGSVLSTACGT...
HsSrc/1-536 363 .......LPQLVDMAAQIASGMAYVERM.NYVHRDLRAANIL...VGEN.......LVCKVADFGLARLIE..DNEYTARQGAKFP
HsCK2A1/1-391 125 .YQTLTD.YDIRFYMYEILKALDYCHSM.GIMHRDVKPHNVM...IDHE.HR....KLRLIDWGLAEFYHPGQ.EYNVRVAS...
HsCDK2/1-298 96 .LTGIPL.PLIKSYLFQLLQGLAFCHSH.RVLHRDLKPQNLL...INTE.G.....AIKLADFGLARAFGVPVRTYTHEVVT...
TgPKAc/1-333 118 .AKRFDN.DTARFYAAQIVDIFDYLHSQ.NIIYRDLKPENML...LDKD.G.....YVKLTDFGFAKVVE...FRTDTLCGT...
TgAGC/1-514 296 .RNCIPT.DQARLYAAEITLAFQYLHQR.HIVYRDLKPENLL...IDSQ.G.....HIKITDFGFAKVVK...DRTWTLCGT...
TgROP5/1-461 274 ......GILARHILTAQLIRLAANLQSK.GLVHGRFTPDNLF...LMPY.G.....PVMLGDVSALWKVG..TR....GPASSVP
TgROP7/1-575 383 ....PLADAARLYLTVQAVRLVAHLQDE.GVVHGKIMPDSFC...LKRE.G.....GLYLRDFGSLVRAG..TK....VVAENA.
TgROP8/1-575 393 ....SLVHHARLQLTLQVIRLLAGLHHY.GLVHTFFRAVDIV...LDQR.G.....GVFLTGFEHLVRDG..DR....VVSTVC.
TgROP16/1-707 509 ...SQPPFLFQRAVAREAIIALAKLHEL.GFAHGDVKLNNMM...IDVH.G.....FGHMLDMGSVRPVDSCVSEEDKY..Y...
TgROP18/1-554 380 ....EFGLVVRMYLSSQAIKLVANVQAQ.GIVHTDIKPANFL...LKKD.G.....RLFLGDFGTYRINNSVGR.......AIGT
TgRON13/1-1375 561 LREIFSSREALIGLTSKLVKPFVVMQNLYSLGHFDIKPPNLLYKYFPGEKGRASRLSVAAGDFGMAGLLHG....DMILRGT...

Subdomain VIII
Subdomain IX
APE
D WS
HsPKA/1-351 203 PEYLAPEIILS.........KG..YNKAVDWWALGVLIYEMAAGYPPFFADQP.IQIYEKIVS...................G..
HsGSK3b/1-420 220 RYYRAPELIFG........ATD..YTSSIDVWSAGCVLAELLLG.QPIFPGDSGVDQLVEIIKVLGTPTREQI.REMNPNYT...
HsCAMK1/1-370 182 PGYVAPEVLAQ.........KP..YSKAVDCWSIGVIAYILLCGYPPFYDEND.AKLFEQILK..................A..
HsSrc/1-536 429 IKWTAPEAALY.........GR..FTIKSDVWSFGILLTELTTKGRVPYPGMVNREVLDQVERGYRMPCP...............
HsCK2A1/1-391 195 RYFKGPELLVD........YQM..YDYSLDMWSLGCMLASMIFRKEPFFHGHDNYDQLVRIAKVLGTEDLYDYIDKYNIELDPRF
HsCDK2/1-298 166 LWYRAPEILLG........CKY..YSTAVDIWSLGCIFAEMVTR.RALFPGDSEIDQLFRIFRTLGTPDEVVW.PGVTS..MPDY
TgPKAc/1-333 185 PEYIAPEVLLN.........KG..HGKPVDWWTLGILIYEMMVGYPPFLDDDP.LGIYQKILA...................G..
TgAGC/1-514 363 HEYLAPESITR.........RG..HGLPVDWWALGILLFEMLAGHPPFVDENP.LGIYRKIIA...................G..
TgROP5/1-461 337 VTYAPR.........EFLNANTATFTHALNAWQLGLSIYRVWCLFLPFGLVTPGIKGSWKRPSLL.....................
TgROP7/1-575 447 QGFSPPEVRGSRGGLLFG.PRKTQMTHGMDAWGLGATIFFIWCFKAPTTGPEEEY.........................
TgROP8/1-575 457 RGFEPPELEARRATISYHRDRRTLMTFSFDAWALGLVIYWIWCADLPNTKDAPLG.........................
TgROP16/1-707 576 LRLWAPELAKSQHT...SQQTCLKRGALDVWALGLAIFEFVCFNRLPYSLSNLPSSLWSRVEHLSRLRLSDF.........
TgROP18/1-554 444 PGYEPPERPFQATG........ITYTFPTDAWQLGITLYCIWCKERPT..........PADGIWD................
TgRON13/1-1375 639 LAFMAPEMERV........SGGLVAKPSYDVYALALTLASFWTAATELRDHYPWV...EKCIK....PTLKKM....K..DAP..

Subdomain XI
R
HsPKA/1-351 255 ........KVRFPSHF.........................SSDLKDLLRNLLQVDLTKRFGNLKNGVNDIKNHKWFATTD...
HsGSK3b/1-420 290 EFKFPQIKAHPWTKVFR.....................PRTPPEAIALCSRLLEYTPTARLTPLE.....ACAHSFFDELRDPN
HsCAMK1/1-370 234 ........EYEFDSPYW.....................DDISDSAKDFIRHLMEKDPEKRFTCEQ.....ALQHPWIAGDTALD
HsSrc/1-536 488 ......................................PECPESLHDLMCQCWRKEPEERPTFEYL...QAFLEDYFTSTEPQY
HsCK2A1/1-391 270 NDILGRHSRKRWERFVHSENQ.................HLVSPEALDFLDKLLRYDHQSRLTARE.....AMEHPYFYTVVKDQ
HsCDK2/1-298 237 KPSFPKWARQDFSKVVP.....................PL.DEDGRSLLSQMLHYDPNKRISAKA.....ALAHPFFQDVTKPV
TgPKAc/1-333 237 ........KFVFPKFF.......................DKDAKVLVKKLLQADLSKRWGNLKNGVRDIKECRWFCTVV...
TgAGC/1-514 415 ........KIEFPRSF.......................DYAAKSLVKRLLTHDPLKRYGCLKDGAEDVKNHKFFKGID...
TgROP5/1-461 393 ..............VPGTDSLSFFPC.............APVPDFVETLIRRFLNFDRRRRLLPLE.....AMETPEFLQLQNEI
TgROP7/1-575 501 .................SIEFLFSLC.............RRAPENVKLLVYKLINPSVEARLLALQ.....ATETPEYREMEEQL
TgROP8/1-575 512 .................GSDWIFRRC.............KNIPQPVRALLAGFLRYPQENRLLPLQ.....AMETPEYEQLRTEL
TgROP16/1-707 645 ......SAK.................DC............NESDPAVMGIVAQFLNPNPEERPELPKF....VSSYTFFRQAPGVT
TgROP18/1-554 491 ...................YLHFADCP.............STPELVQDLIRSLLNRDPQKRMLPLQ.....ALETAAFKEMDSVV
TgRON13/1-1375 701 EFTFLRFASKTGPKLYEADTIYALSTCFAVGGKVEKLYHTGMPLLIRLKLSQMADPEPLARVSMRH......ARFVFKAYAMLD

HsPKA/1-351 303 ......................WIAIYQRKVEAPFIPKF......KGPGDTSNFDDYEEEE...IRVSINEKCG.......K..
HsGSK3b/1-420 348 .VKLPNGRDTPA..........LFNFTTQ.....................ELSSNPPLATI.......LIPPHARIQAAASTP..
HsCAMK1/1-370 284 .KNIHQSVSEQI..KKNF.AKSKWKQAFNATAVVRHMRKLQLGTSQEGQGQTASHGELLTP....VAGGPAAGCCCRDCCVEPGT
HsSrc/1-536 531 ....QPGENL...
HsCK2A1/1-391 332 .ARMGSSSMPGGS....T.PVS..SANMMS.................GISSVPTPSPL.......GPLAGSPVIAA.ANP..
HsCDK2/1-298 294 .PHLRL..
TgPKAc/1-333 285 ......................FEDLRAKKVVARYKPTV......KGMDDLSNFEAYPDEK...DDAVPVDPMK.......D..
TgAGC/1-514 463 ......................WVKCYNKNIRAPYLPNT......RGPQDSSMFDKYAEST...ESSSPPIGAA.......E..
TgROP5/1-461 446 SRRLSTGQPTAAPSVA..................................
TgROP7/1-575 551 SAASRLYSGDGTLTGGDD.DMPPPET........................
TgROP8/1-575 562 SAVLPLYQTDGEPA....................................
TgROP16/1-707 692 SHLTRIPTTELSS............HRM......................
TgROP18/1-554 539 KGAAQN.........FEQQEHLHTE.........................
TgRON13/1-1375 779 .KLLRAPQSEANAETREE.QLKQLQSLHIVQFLLFYLRMEPL...TAARDNTQSYRRLARALLDFARLDPVYQAA.........T

HsPKA/1-351 347 .EFSEF....
HsGSK3b/1-420 392 .TNATAA..SDANTGDRGQTNNAASASASNST....
HsCAMK1/1-370 361 ELSPTLPHQL....
HsSrc/1-536 ....
HsCK2A1/1-391 379 .LGMPVP..A..AAGAQQ....
HsCDK2/1-298 ....
TgPKAc/1-333 329 .PFTTW....
TgAGC/1-514 507 .QQAFFDNF....
TgROP5/1-461 ....
TgROP7/1-575 ....
TgROP8/1-575 ....
TgROP16/1-707 ....
TgROP18/1-554 ....
TgRON13/1-1375 850 ETVQPLPYEFFTEQKDWQNVKVEVSGSEVDETIRKLRTSLTRDRSLSEDSWADLVDIMFGVSLDGLREVVTRVVYSRKTFLLEEK

```
HsPKA/1-351

HsPKA/1-351              .....................................................................................
HsGSK3b/1-420            .....................................................................................
HsCAMK1/1-370            .....................................................................................
HsSrc/1-536              .....................................................................................
HsCK2A1/1-391            .....................................................................................
HsCDK2/1-298             .....................................................................................
TgPKAc/1-333             .....................................................................................
TgAGC/1-514              .....................................................................................
TgROP5/1-461             .....................................................................................
TgROP7/1-575             .....................................................................................
TgROP8/1-575             .....................................................................................
TgROP16/1-707            .....................................................................................
TgROP18/1-554            .....................................................................................
TgRON13/1-1375      935  IGNAVKEAVAATYKFDPNTQLIAEDAPDRLFEVVRTDLGLSYPDDSELGRFLVHRVSKSHTAWATVDRLARQALRLALRREERTR
TgRON13/1-1375           α30 α31 α32
```

```
HsPKA/1-351

HsPKA/1-351              .....................................................................................
HsGSK3b/1-420            .....................................................................................
HsCAMK1/1-370            .....................................................................................
HsSrc/1-536              .....................................................................................
HsCK2A1/1-391            .....................................................................................
HsCDK2/1-298             .....................................................................................
TgPKAc/1-333             .....................................................................................
TgAGC/1-514              .....................................................................................
TgROP5/1-461             .....................................................................................
TgROP7/1-575             .....................................................................................
TgROP8/1-575             .....................................................................................
TgROP16/1-707            .....................................................................................
TgROP18/1-554            .....................................................................................
TgRON13/1-1375     1020  QVYEQLLSGEKPSSESEKAFFDSVFSAVSVVSEANYFGLFWDFPSAGLFGVPPEEMQAYVRKTHLAFVGKMWPVETQKKILEAAV
TgRON13/1-1375           α33 α34 TT α35 α36
```

```
HsPKA/1-351

HsPKA/1-351              .....................................................................................
HsGSK3b/1-420            .....................................................................................
HsCAMK1/1-370            .....................................................................................
HsSrc/1-536              .....................................................................................
HsCK2A1/1-391            .....................................................................................
HsCDK2/1-298             .....................................................................................
TgPKAc/1-333             .....................................................................................
TgAGC/1-514              .....................................................................................
TgROP5/1-461             .....................................................................................
TgROP7/1-575             .....................................................................................
TgROP8/1-575             .....................................................................................
TgROP16/1-707            .....................................................................................
TgROP18/1-554            .....................................................................................
TgRON13/1-1375     1105  RVTVRGLNASLPASLVDVYATVFAALPTKAPVSPPFLYGLEREEYSSLLFDAKLPEFKEMVAFWATRHELNIAVQTAVGKIPDAT
TgRON13/1-1375           α37 α38 TT α39 α40
```

```
HsPKA/1-351

HsPKA/1-351              .....................................................................................
HsGSK3b/1-420            .....................................................................................
HsCAMK1/1-370            .....................................................................................
HsSrc/1-536              .....................................................................................
HsCK2A1/1-391            .....................................................................................
HsCDK2/1-298             .....................................................................................
TgPKAc/1-333             .....................................................................................
TgAGC/1-514              .....................................................................................
TgROP5/1-461             .....................................................................................
TgROP7/1-575             .....................................................................................
TgROP8/1-575             .....................................................................................
TgROP16/1-707            .....................................................................................
TgROP18/1-554            .....................................................................................
TgRON13/1-1375     1190  NLSDEDIEKQLEGMLPAHLRSPSPARFGWPPEAVADNIRLFIREAKDELALHGPDMVHNRIRVNGRSKPPRRAAFLFHEIFRKAI
TgRON13/1-1375           η4 TT α41 α42 β11 β12
```

```
HsPKA/1-351

HsPKA/1-351              .....................................................................................
HsGSK3b/1-420            .....................................................................................
HsCAMK1/1-370            .....................................................................................
HsSrc/1-536              .....................................................................................
HsCK2A1/1-391            .....................................................................................
HsCDK2/1-298             .....................................................................................
TgPKAc/1-333             .....................................................................................
TgAGC/1-514              .....................................................................................
TgROP5/1-461             .....................................................................................
TgROP7/1-575             .....................................................................................
TgROP8/1-575             .....................................................................................
TgROP16/1-707            .....................................................................................
TgROP18/1-554            .....................................................................................
TgRON13/1-1375     1275  AFKKDISVLQFNQFFTDILKQSFDPQCRRFIAEVKKRVKSAPAEYVRVADTEAVAPLFEGEGKDILKLVAVDPAARASDPEPNNC
TgRON13/1-1375           α43 η5 β13 TT α44 β14
```

HsPKA/1-351		
**HsPKA/1-351**		. . . .
**HsGSK3b/1-420**		. . . .
**HsCAMK1/1-370**		. . . .
**HsSrc/1-536**		. . . .
**HsCK2A1/1-391**		. . . .
**HsCDK2/1-298**		. . . .
**TgPKAc/1-333**		. . . .
**TgAGC/1-514**		. . . .
**TgROP5/1-461**		. . . .
**TgROP7/1-575**		. . . .
**TgROP8/1-575**		. . . .
**TgROP16/1-707**		. . . .
**TgROP18/1-554**		. . . .
**TgRON13/1-1375**	**1360**	F L W T Q A F L D D K T I V V S
TgRON13/1-1375		η6 **TTT** β15
