# Supplementary material for: Structural insights into an atypical secretory pathway kinase crucial for Toxoplasma gondii invasion
Source: Nat Commun. 2021 Jun 18;12:3788. doi: 10.1038/s41467-021-24083-y (PMC8213820; doi:10.1038/s41467-021-24083-y)
Supplement: Supplementary file 4 — Supplementary Data 2 [file 41467_2021_24083_MOESM4_ESM.pdf]

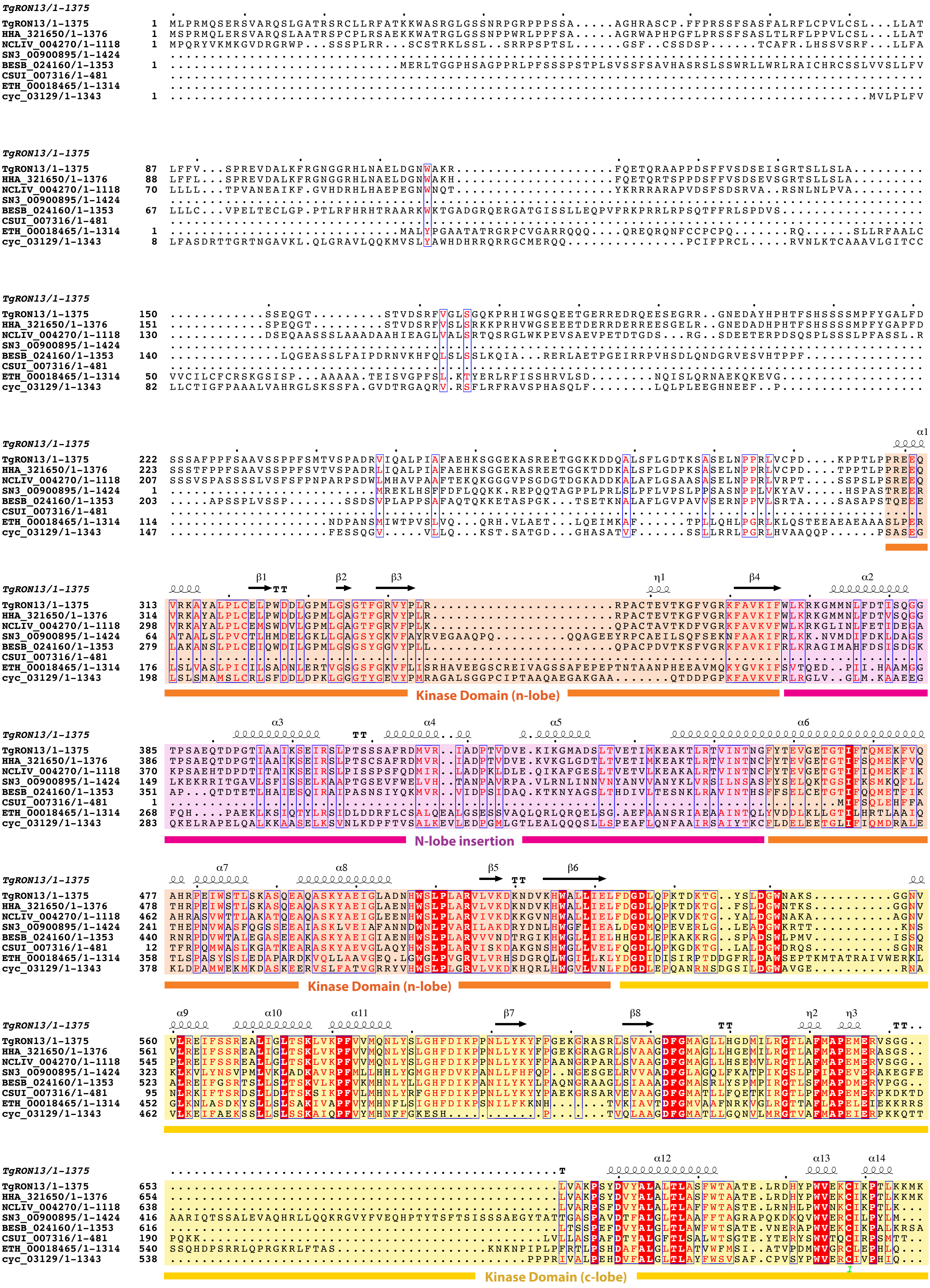
TgRON13/1-1375
HHA_321650/1-1376
NCLIV_004270/1-1118
SN3_00900895/1-1424
BESB_024160/1-1353
CSUI_007316/1-481
ETH_00018465/1-1314
cyc_03129/1-1343
Kinase Domain (n-lobe)
N-lobe insertion
Kinase Domain (n-lobe)
Kinase Domain (c-lobe)

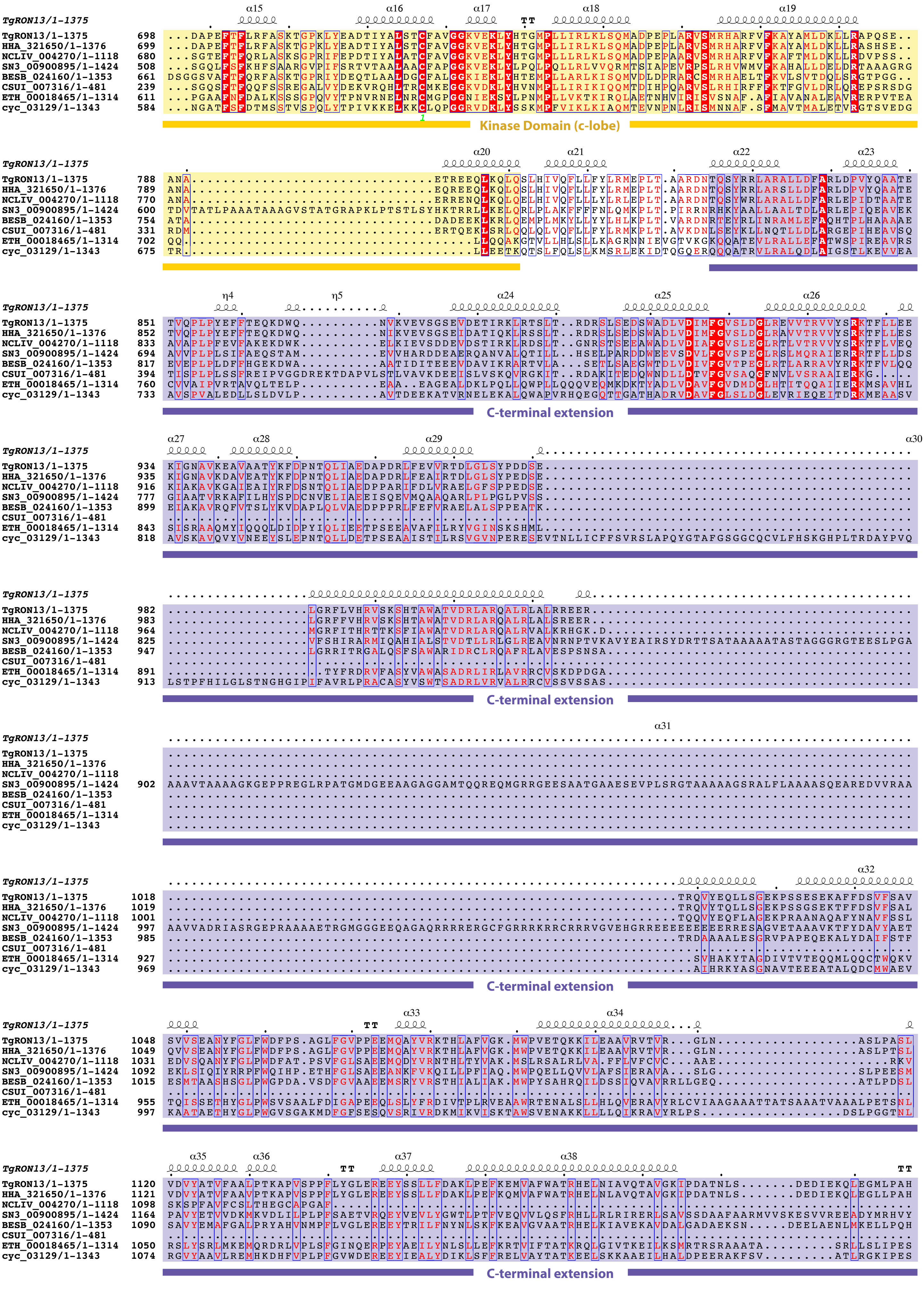
TgRON13/1-1375
HHA_321650/1-1376
NCLIV_004270/1-1118
SN3_00900895/1-1424
BESB_024160/1-1353
CSUI_007316/1-481
ETH_00018465/1-1314
cyc_03129/1-1343
Kinase Domain (c-lobe)
C-terminal extension

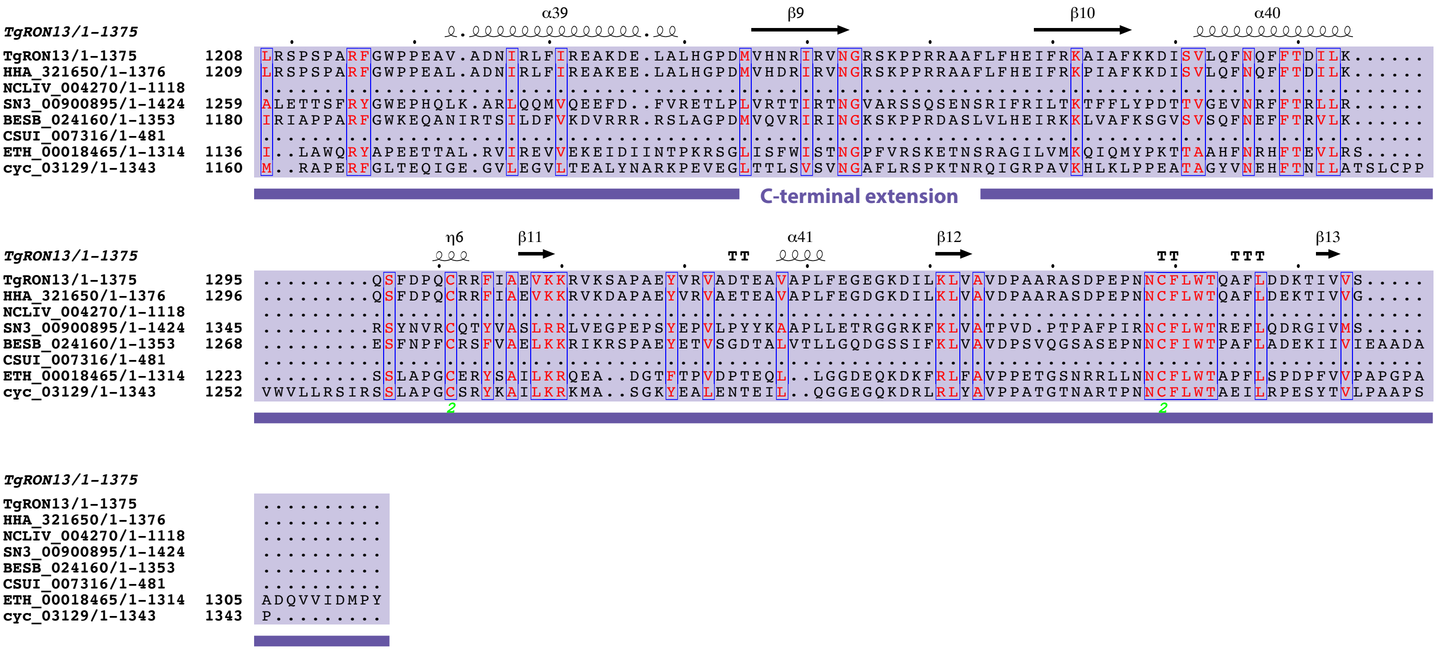
TgRON13/1-1375
α39
β9
β10
α40
TgRON13/1-1375 1208 LRSPSPARFGWPPEAV.ADNIRLFIREAKDE.LALHGPDMVHNRIRVNGRSKPPRRAAFLFHEIFRKAIAFKKDISVLQFNQFFTDILK......
HHA_321650/1-1376 1209 LRSPSPARFGWPPEAL.ADNIRLFIREAKEE.LALHGPDMVHDRIRVNGRSKPPRRAAFLFHEIFRKPIAFKKDISVLQFNQFFTDILK......
NCLIV_004270/1-1118
SN3_00900895/1-1424 1259 ALETTSFRYGWEPHQLK.ARLQQMVQEEFD..FVRETLPLVRTTIRTNGVARSSQSENSRIFRILTKTFFLYPDTTVGEVNRFFTRLLR......
BESB_024160/1-1353 1180 IRIAPPARFGWKEQANIRTSILDFVKDVRRR.RSLAGPDMVQVRIRINGKSKPPRDASLVLHEIRKKLVAFKSGVSVSQFNEFFTRVLK......
CSUI_007316/1-481
ETH_00018465/1-1314 1136 I..LAWQRYAPEETTAL.RVIREVVEKEIDIINTPKRSGLISFWISTNGPFVRSKETNSRAGILVMKQIQMYPKTTAAHFNRHFTEVLRS.....
cyc_03129/1-1343 1160 M..RAPERFGLTEQIGE.GVLEGVLTEALYNARKPEVEGLTTLSVSVNGAFLRSPKTNRQIGRPAVKHLKLPPEATAGYVNEHFTNILATSLCPP
C-terminal extension
TgRON13/1-1375
η6
β11
TT
α41
β12
TT
TTT
β13
TgRON13/1-1375 1295 .........QSFDPQCRRFIAEVKKRVKSAPAEYVRVADTEAVAPLFEGEGKDILKLVAVDPAARASDPEPNNCFLWTQAFLDDKTIVVS.....
HHA_321650/1-1376 1296 .........QSFDPQCRRFIAEVKKRVKDAPAEYVRVAETEAVAPLFEGDGKDILKLVAVDPAARASDPEPNNCFLWTQAFLDEKTIVVG.....
NCLIV_004270/1-1118
SN3_00900895/1-1424 1345 .........RSYNVRCQTYVASLRRLVEGPEPSYEPVLPYYKAAPLLETRGGRKFKLVATPVD.PTPAFPIRNCFLWTREFLQDRGIVMS.....
BESB_024160/1-1353 1268 .........ESFNPFCRSFVAELKKRIKRSPAEYETVSGDTALVTLLGQDGSSIFKLVAVDPSVQGSASEPNNCFIWTPAFLADEKIIVIEAADA
CSUI_007316/1-481
ETH_00018465/1-1314 1223 .........SSLAPGCERYSAILKRQEA..DGTFTPVDPTEQL..LGGDEQKDKFRLFAVPPETGSNRRLLNNCFLWTAPFLSPDPFVVPAPGPA
cyc_03129/1-1343 1252 VWVLLRSIRSSLAPGCSRYKAILKRKMA..SGKYEALENTEIL..QGGEGQKDRLRLYAVPPATGTNARTPNNCFLWTAEILRPESYTVLPAAPS
2
2
TgRON13/1-1375
TgRON13/1-1375 ....
HHA_321650/1-1376 ....
NCLIV_004270/1-1118 ....
SN3_00900895/1-1424 ....
BESB_024160/1-1353 ....
CSUI_007316/1-481 ....
ETH_00018465/1-1314 1305 ADQVVIDMPY
cyc_03129/1-1343 1343 P.........
