# Supplementary material for: Structural insights into an atypical secretory pathway kinase crucial for Toxoplasma gondii invasion
Source: Nat Commun. 2021 Jun 18;12:3788. doi: 10.1038/s41467-021-24083-y (PMC8213820; doi:10.1038/s41467-021-24083-y)
Supplement: Supplementary file 9 — Supplementary Data 7 [file 41467_2021_24083_MOESM9_ESM.pdf]

TgRON13/1-1375

```
TgRON13/1-1375         ....................................................................................................
HHA_321650/1-1376      ....................................................................................................
NCLIV_004270/1-1118    ....................................................................................................
SN3_00900895/1-1424    ....................................................................................................
BESB_024160/1-1353     ....................................................................................................
CSUI_007316/1-481      ....................................................................................................
ETH_00018465/1-1314    ....................................................................................................
cyc_03129/1-1343       ....................................................................................................
PF3D7_1321100/1-3447   MNFFLFFRNIFLLNTIIFILNQYIKRKDYGSRFFIFSLKIKSSKEEFTKNVHRKATNTKEDKSAYKNEKKEIPYRNNNKRKDKNKKGNDNKKILEKGLK
```

TgRON13/1-1375

```
TgRON13/1-1375         ....................................................................................................
HHA_321650/1-1376      ....................................................................................................
NCLIV_004270/1-1118    ....................................................................................................
SN3_00900895/1-1424    ....................................................................................................
BESB_024160/1-1353     ....................................................................................................
CSUI_007316/1-481      ....................................................................................................
ETH_00018465/1-1314    ....................................................................................................
cyc_03129/1-1343       ....................................................................................................
PF3D7_1321100/1-3447   DTNSNIKSSTSSNTISNVSSKLSDNSNISNTFTNIKIQKLKDKHNKKNSDIKNVSTDKKKENDNNKKNNNNNKKNNKKIPIIYIPFNDIKNSPYIRGKLK
```

TgRON13/1-1375

```
TgRON13/1-1375         ....................................................................................................
HHA_321650/1-1376      ....................................................................................................
NCLIV_004270/1-1118    ....................................................................................................
SN3_00900895/1-1424    ....................................................................................................
BESB_024160/1-1353     ....................................................................................................
CSUI_007316/1-481      ....................................................................................................
ETH_00018465/1-1314    ....................................................................................................
cyc_03129/1-1343       ....................................................................................................
PF3D7_1321100/1-3447   LVYVKSFYEECQKELYEAKFQFFKNNKIYISTFAVVKNTLSENIDQTEKWVTADMKILQKNSYNISVTCNDSHEKQIQDISFTKQEKEEFELMRKSLKN
```

TgRON13/1-1375

```
TgRON13/1-1375         ....................................................................................................
HHA_321650/1-1376      ....................................................................................................
NCLIV_004270/1-1118    ....................................................................................................
SN3_00900895/1-1424    ....................................................................................................
BESB_024160/1-1353     ....................................................................................................
CSUI_007316/1-481      ....................................................................................................
ETH_00018465/1-1314    ....................................................................................................
cyc_03129/1-1343       ....................................................................................................
PF3D7_1321100/1-3447   VVNITYDHFLYNIDTFYNAKRKYLEYINFFFRNPHIKSNMDIKKNISSNITIQKDNILLKRLGSFNKTKYILFRRMQRNNRIMCMVLTDPKRLENRFIL
```

TgRON13/1-1375

```
TgRON13/1-1375         ....................................................................................................
HHA_321650/1-1376      ....................................................................................................
NCLIV_004270/1-1118    ....................................................................................................
SN3_00900895/1-1424    ....................................................................................................
BESB_024160/1-1353     ....................................................................................................
CSUI_007316/1-481      ....................................................................................................
ETH_00018465/1-1314    ....................................................................................................
cyc_03129/1-1343       ....................................................................................................
PF3D7_1321100/1-3447   YNMLRDHIIEKEGFKDVIIFETDDNNIYNYDSKLSKDIRILSNKFEFEILMDEHAYINKPYEYFYLNKYIPVHFELQNKGIINYNNIFSWLCEHFINNS
```

TgRON13/1-1375

```
TgRON13/1-1375         ....................................................................................................
HHA_321650/1-1376      ....................................................................................................
NCLIV_004270/1-1118    ....................................................................................................
SN3_00900895/1-1424    ....................................................................................................
BESB_024160/1-1353     ....................................................................................................
CSUI_007316/1-481      ....................................................................................................
ETH_00018465/1-1314    ....................................................................................................
cyc_03129/1-1343       ....................................................................................................
PF3D7_1321100/1-3447   QYKYYYPHFYLSKHSDKNDMGIRNISFYQKEDAYTFIENNKKNINKNDKCKTYDTCEKDDTYRTDKKSKLKNINRIYNTKYALSKKKNRNHKNNEINNI
```

TgRON13/1-1375

```
TgRON13/1-1375         ....................................................................................................
HHA_321650/1-1376      ....................................................................................................
NCLIV_004270/1-1118    ....................................................................................................
SN3_00900895/1-1424    ....................................................................................................
BESB_024160/1-1353     ....................................................................................................
CSUI_007316/1-481      ....................................................................................................
ETH_00018465/1-1314    ....................................................................................................
cyc_03129/1-1343       ....................................................................................................
PF3D7_1321100/1-3447   CSHKKEEKHMFNNYNISYNNMKYINKHNKFSFLELKEKLKSDNNEREDKKKKDESFEKNETHLMNNDDNNIGDDEFNSEVNNDNNGSYESNFFYDMINE
```

TgRON13/1-1375

```
TgRON13/1-1375         ....................................................................................................
HHA_321650/1-1376      ....................................................................................................
NCLIV_004270/1-1118    ....................................................................................................
SN3_00900895/1-1424    ....................................................................................................
BESB_024160/1-1353     ....................................................................................................
CSUI_007316/1-481      ....................................................................................................
ETH_00018465/1-1314    ....................................................................................................
cyc_03129/1-1343       ....................................................................................................
PF3D7_1321100/1-3447   ISNKIENSDSYLNEEDIYWDDDIFFDTNEYIDDTSTITTSTNNNNIRTDQNGDNKKDDSNKDIISIQKVNDSTEVNESNKIDDTKKGESYTLNNIKGYI
```

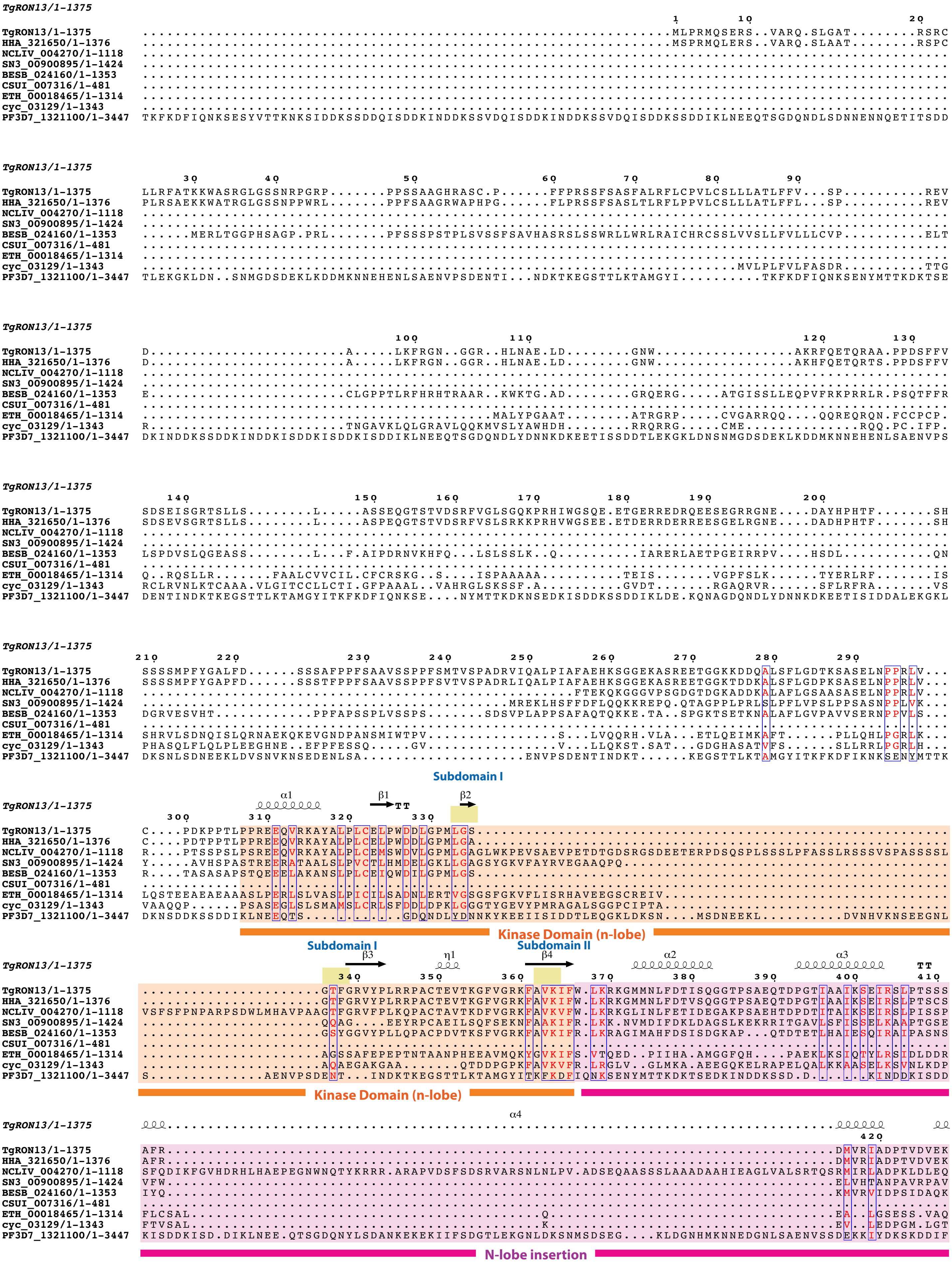
TgRON13/1-1375
HHA_321650/1-1376
NCLIV_004270/1-1118
SN3_00900895/1-1424
BESB_024160/1-1353
CSUI_007316/1-481
ETH_00018465/1-1314
cyc_03129/1-1343
PF3D7_1321100/1-3447
Subdomain I
Kinase Domain (n-lobe)
Subdomain II
N-lobe insertion

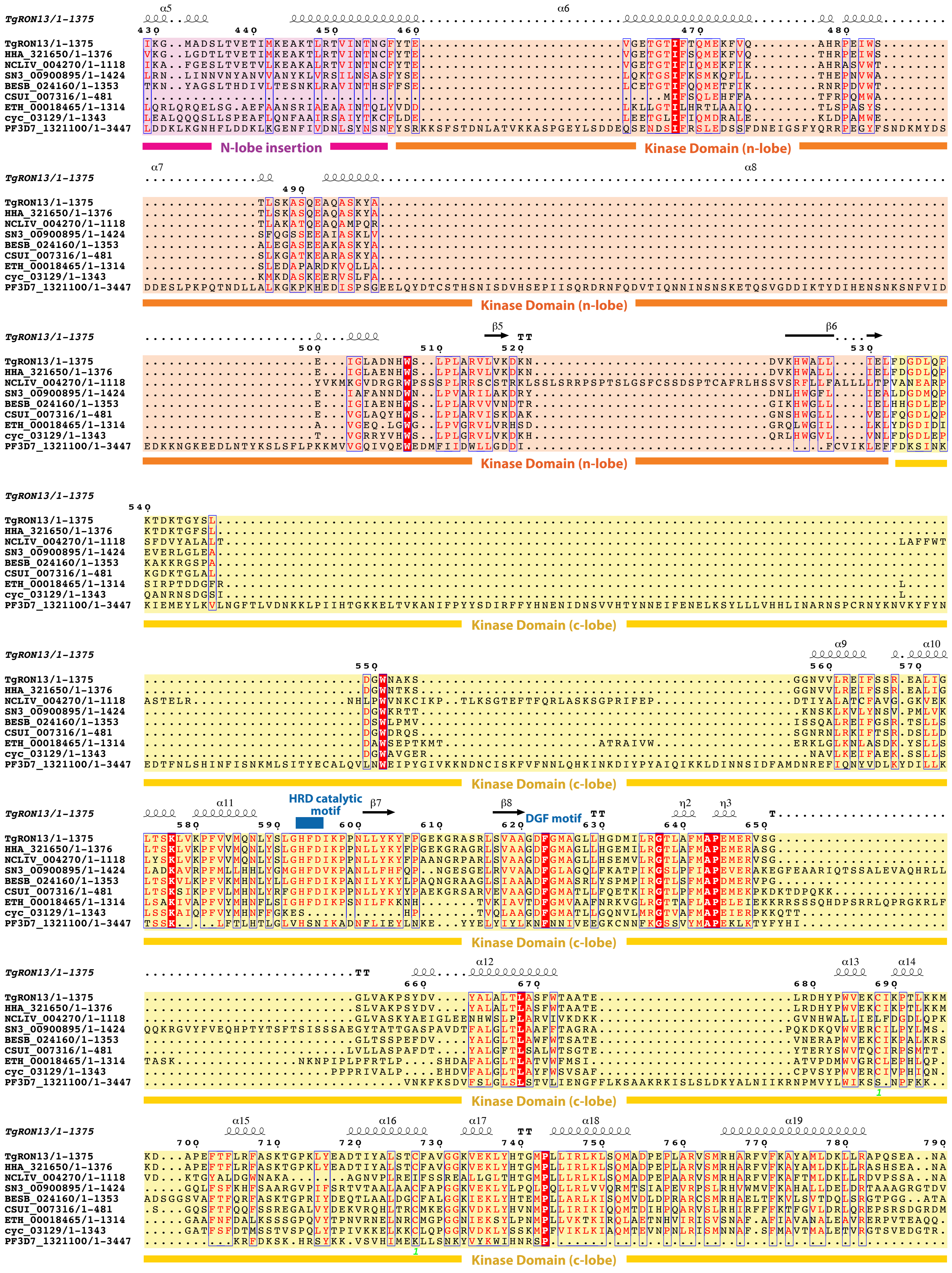

TgRON13/1-1375
HHA_321650/1-1376
NCLIV_004270/1-1118
SN3_00900895/1-1424
BESB_024160/1-1353
CSUI_007316/1-481
ETH_00018465/1-1314
cyc_03129/1-1343
PF3D7_1321100/1-3447
N-lobe insertion
Kinase Domain (n-lobe)
Kinase Domain (c-lobe)
HRD catalytic motif
DGF motif

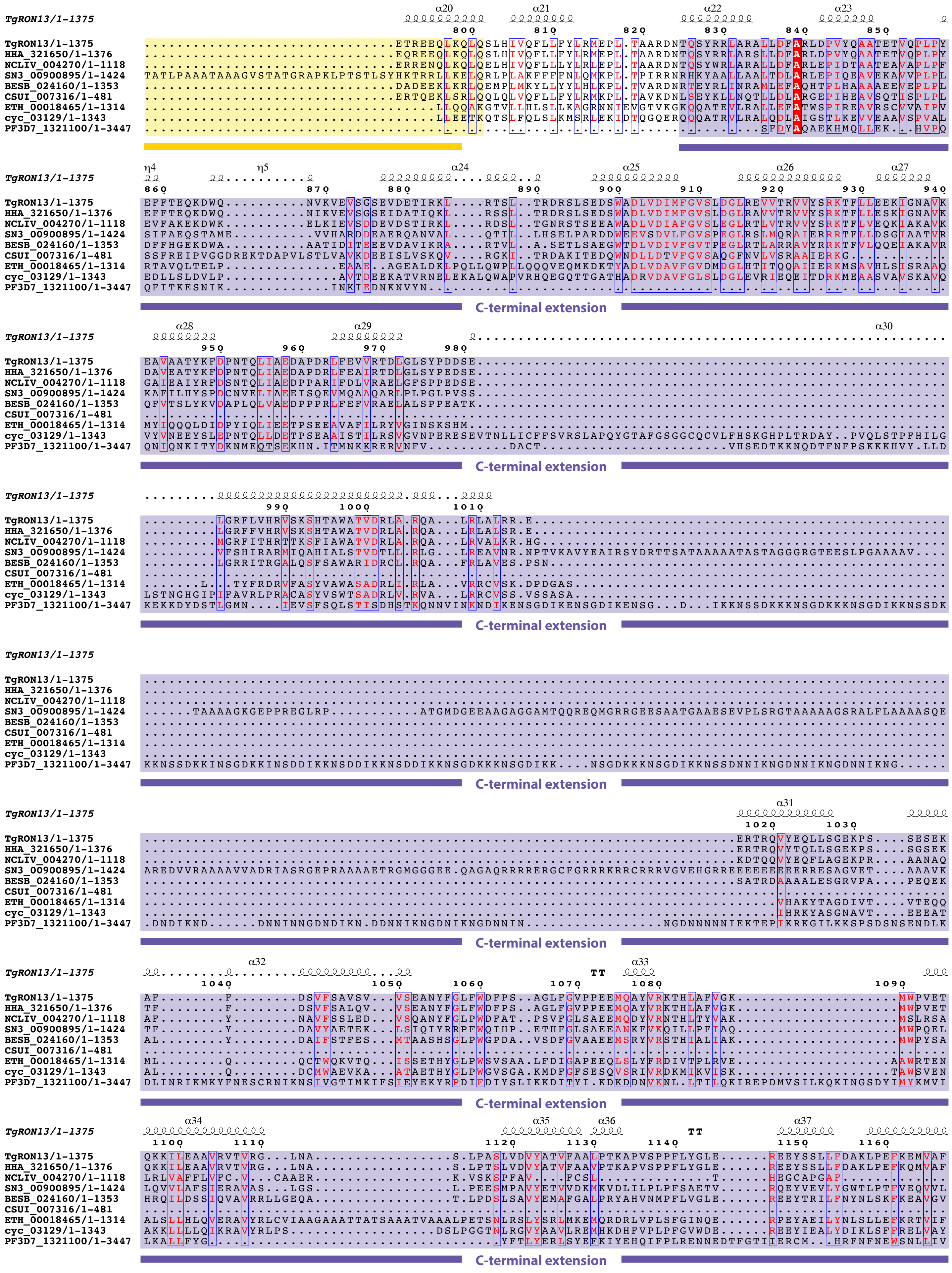
TgRON13/1-1375
HHA_321650/1-1376
NCLIV_004270/1-1118
SN3_00900895/1-1424
BESB_024160/1-1353
CSUI_007316/1-481
ETH_00018465/1-1314
cyc_03129/1-1343
PF3D7_1321100/1-3447
α20 α21 α22 α23
η4 η5 α24 α25 α26 α27
α28 α29 α30
α31
α32 α33
α34 α35 α36 α37
C-terminal extension

```
                              α38                                                                  α39
TgRON13/1-1375                                                                      TT
                                     1170      1180      1190           1200           1210      1220      1230
TgRON13/1-1375         WA.......TRHELNIAVQTAVGKIPDATNLS.D.E...................DIEKQLEGML.....PAHLRSPSPARFGWPPEAV.ADNIRLFIR
HHA_321650/1-1376      WA.......TRHELNLAVQTAVGKIPDATNLS.D.E...................DIEKQLEGLL.....PAHLRSPSPARFGWPPEAL.ADNIRLFIR
NCLIV_004270/1-1118    ...................................................................................................
SN3_00900895/1-1424    QS.......FRHLLRLRIRERLSAVSSDAAFAARMVV..............SKESVVREEADYMR.....HVYALETTSFRYGWEPHQLKARL.QQMVQ
BESB_024160/1-1353     AA.......TRHELKIAVEKAVDALGADAEKSNDEE...................LAENLMKELL.....PQHIRIAPPARFGWKEQANIRTSILDFVK
CSUI_007316/1-481      ...................................................................................................
ETH_00018465/1-1314    TA.......TKRQLGIVTKEILKSMRTRSRAAATAS.......................RLLSLI.....PES..ILAWQRYAPEETTAL.RVIREVVE
cyc_03129/1-1343       TA.......TKEELSKKAAEILHALDPEERAKFSVA.......................TLRGKI.....PES..MRAPERFGLTEQIGE.GVLEGVLT
PF3D7_1321100/1-3447   QHIYNNYMSKKKYTNLYINLHILNIKPNDEKKKEMEELYDKHMTEVFKDIDYNDNFNNFLLYMKKESEDISNTLKEKNNMKTGWDNFHINYDDIYD...

                                          β9
TgRON13/1-1375
                          1240      1250                1260
TgRON13/1-1375         EAK.DELALHGPDMVHNRI.................RVNGRSKPPRRAA..................................................
HHA_321650/1-1376      EAK.EELALHGPDMVHDRI.................RVNGRSKPPRRAA..................................................
NCLIV_004270/1-1118    ...................................................................................................
SN3_00900895/1-1424    EEF..DFVRETLPLVRTTI.................RTNGVARSSQSEN..................................................
BESB_024160/1-1353     DVR.RRRSLAGPDMVQVRI.................RINGKSKPPRDAS..................................................
CSUI_007316/1-481      ...................................................................................................
ETH_00018465/1-1314    KEIDIINTPKRSGLISFWI.................STNGPFVRSKETN..................................................
cyc_03129/1-1343       EALYNARKPEVEGLTTLSV.................SVNGAFLRSPKTN..................................................
PF3D7_1321100/1-3447   .....KHDEKDLSLIRKTILKLDNSVIRDMNINLAILLNDMFWKHKNRCKGVNYNHMKVKYADNTIKEFNIQKNPIIDLHKPIGSIVKGLIKNDDIMIS

TgRON13/1-1375

TgRON13/1-1375         ...................................................................................................
HHA_321650/1-1376      ...................................................................................................
NCLIV_004270/1-1118    ...................................................................................................
SN3_00900895/1-1424    ...................................................................................................
BESB_024160/1-1353     ...................................................................................................
CSUI_007316/1-481      ...................................................................................................
ETH_00018465/1-1314    ...................................................................................................
cyc_03129/1-1343       ...................................................................................................
PF3D7_1321100/1-3447   KGHTNINTLDYIIPINIHLDVILQNSLHFVYTSTLIKCAEKMDKKGIIYHRDITYNRKKETIKIKIGLISNSYFYLNVDISYLRYHEITLKDIIYFIYR

                                β10          α40                                        η6      β11
TgRON13/1-1375
                                 1270      1280      1290                                 1300      1310      1320
TgRON13/1-1375         ........FLFHEIFRKAIAFKKDISVLQFNQFFTDILK.................................QSFDPQCRRFIA.EVKKRVKSAPAEYV
HHA_321650/1-1376      ........FLFHEIFRKPIAFKKDISVLQFNQFFTDILK.................................QSFDPQCRRFIA.EVKKRVKDAPAEYV
NCLIV_004270/1-1118    ...................................................................................................
SN3_00900895/1-1424    ........SRIFRILTKTFFLYPDTTVGEVNRFFTRLLR.................................RSYNVRCQTYVA.SLRRLVEGPEPSYE
BESB_024160/1-1353     ........LVLHEIRKKLVAFKSGVSVSQFNEFFTRVLK.................................ESFNPFCRSFVA.ELKKRIKRSPAEYE
CSUI_007316/1-481      ...................................................................................................
ETH_00018465/1-1314    ........SRAGILVMKQIQMYPKTTAAHFNRHFTEVLR................................SSSLAPGCERYSA.ILKRQEA..DGTFT
cyc_03129/1-1343       ........RQIGRPAVKHLKLPPEATAGYVNEHFTNILA..................TSLCPPVWVLLRSIRSSLAPGCSRYKA.ILKRKMA..SGKYE
PF3D7_1321100/1-3447   NYKSELNKSVRSNIEKRSFLFFDCLLVNEYNTFFYNNSTKKLIKETSFNKLISQLYNINLNDPEYIIKSTIN.YYNHRKSNMPLISLVTISHTIHERWN
                                                                                            2

                              α41                     β12                                               β13
TgRON13/1-1375            TT                                                             TT      TTT
                              1330                  1340                  1350      1360      1370
TgRON13/1-1375         RV..ADTEAVAPLFEG...........EGKDILKLVAVDPA...........ARASDPEPNNCFLWTQAFLDDKTIVVS....................
HHA_321650/1-1376      RV..AETEAVAPLFEG...........DGKDILKLVAVDPA...........ARASDPEPNNCFLWTQAFLDEKTIVVG....................
NCLIV_004270/1-1118    ...................................................................................................
SN3_00900895/1-1424    PV..LPYYKAAPLLET...........RGGRKFKLVATPV............DPTPAFPIRNCFLWTREFLQDRGIVMS....................
BESB_024160/1-1353     TV..SGDTALVTLLGQ...........DGSSIFKLVAVDPS...........VQGSASEPNNCFIWTPAFLADEKIIVIEAA...DA............
CSUI_007316/1-481      ...................................................................................................
ETH_00018465/1-1314    PV..DPTEQ...LLGG..........DEQKDKFRLFAVPPE...........TGSNRRLLNNCFLWTAPFLSPDPFVVPAPG...PAADQVVIDMPY..
cyc_03129/1-1343       AL..ENTEI...LQGG..........EGQKDRLRLYAVPPA...........TGTNARTPNNCFLWTAEILRPESYTVLPAA...PSP...........
PF3D7_1321100/1-3447   GIKKQTDYILQPSINNYSDEYNEELIDPSKLKFKNPSLNIKENMNRIIMRNRYEGYQYRLVKCFDF....LHKKNISYYKDQHTTNLFDQIIIPAVFIH
                                                                              2

TgRON13/1-1375

TgRON13/1-1375         ...................................................................................................
HHA_321650/1-1376      ...................................................................................................
NCLIV_004270/1-1118    ...................................................................................................
SN3_00900895/1-1424    ...................................................................................................
BESB_024160/1-1353     ...................................................................................................
CSUI_007316/1-481      ...................................................................................................
ETH_00018465/1-1314    ...................................................................................................
cyc_03129/1-1343       ...................................................................................................
PF3D7_1321100/1-3447   LKNDDISKINIKLALEHRKIKFNIITDIMNLQIYENQIFTLTDFMYLIFRWLETYISFNDRDHCTFLTDRTQISQYDLVLSIETGKDKINILYKNNEGV

TgRON13/1-1375

TgRON13/1-1375         ...................................................................................................
HHA_321650/1-1376      ...................................................................................................
NCLIV_004270/1-1118    ...................................................................................................
SN3_00900895/1-1424    ...................................................................................................
BESB_024160/1-1353     ...................................................................................................
CSUI_007316/1-481      ...................................................................................................
ETH_00018465/1-1314    ...................................................................................................
cyc_03129/1-1343       ...................................................................................................
PF3D7_1321100/1-3447   IKFIFQNILKNHQVNQYTSEFLMSAIKLNKLKLFFVLKDELKYIFPDIPEGLYFHLKESNYKIINCLKYLDGKNVKLLENNIYDDSYVPVKIKFMNVTV

TgRON13/1-1375

TgRON13/1-1375         ...................................................................................................
HHA_321650/1-1376      ...................................................................................................
NCLIV_004270/1-1118    ...................................................................................................
SN3_00900895/1-1424    ...................................................................................................
BESB_024160/1-1353     ...................................................................................................
CSUI_007316/1-481      ...................................................................................................
ETH_00018465/1-1314    ...................................................................................................
cyc_03129/1-1343       ...................................................................................................
PF3D7_1321100/1-3447   IYRIKKDNFKNIIYNFTKYEVLKHYGKQLFTRFISPINDIENKFRYLFLRDLSYYIDIPRDIKRDKDDFMVSMDSNVYNALNEKHNIYMNNYSSIIEKM
```

TgRON13/1-1375

```
TgRON13/1-1375         ........................................
HHA_321650/1-1376      ........................................
NCLIV_004270/1-1118    ........................................
SN3_00900895/1-1424    ........................................
BESB_024160/1-1353     ........................................
CSUI_007316/1-481      ........................................
ETH_00018465/1-1314    ........................................
cyc_03129/1-1343       ........................................
PF3D7_1321100/1-3447   ITYMNVTYTEPVFIVCVVKHTVLGSKIQLLLREMKYNITS
```
