# Supplementary material for: Structural insights into an atypical secretory pathway kinase crucial for Toxoplasma gondii invasion
Source: Nat Commun. 2021 Jun 18;12:3788. doi: 10.1038/s41467-021-24083-y (PMC8213820; doi:10.1038/s41467-021-24083-y)
Supplement: Supplementary file 10 — Supplementary Data 8 [file 41467_2021_24083_MOESM10_ESM.pdf]

```
                    ....|....| ....|....| ....|....| ....|....| ....|....| ....|....| ....|....| ....|....| ....|....| ....|....|
                        5         15         25         35         45         55         65         75         85         95
Q13554              DEYQLYEDGK GAFSVVRRCV KLCTGEYAAK IINKHQKLER EARICRLLKH SNIVRLHSIS EEGFHYLVFD LVTGELFEDI VCIQQILEAV LHCHQMGVVH
TGGT1_321650        TIFTQMEKVQ AHRPEIWSTL SKASQEAQAS KYAIWSLPLA RVLVKDVKHW ALLIELFDLQ PKTDKYSLDG WNAKVVLREI FLTSKLVKPF VVMQNYSLGH
PF3D7_0424500       KKVPASIWRQ YIILNEHKGE YSLGENFVME AISAPGIAPK FYGILYDKPN DNVLKKFMLI NQLQLIMISE LYGEDVFKYV KILHESLKLI TTLHETGLSH
PF3D7_1016400       KKIPVDIWKQ FELMNAYNGE YVLCGNYVME ATTAPGITPK LYKILYNTPD CMFLNVFILS ARLKCVIISE LFGEDIYTYL TILFECLNVL RKLHDAGLCH
PF3D7_1200800       KRIPVEWWKQ FNLMEKYDGE YLVKANYVME GVASPGIAPK LLKILYNVNH DIMYKFKIYE FNNMLVMVSE LFGEDLFDFN KLLYKCLRLL VRLHSAGLSH
PF3D7_0902100       KKVPLNSWEL YNKMDIYHGE FLDGANFVME AMVLPGITPK FYNLLYYSEL KGLLMFCIDI FKNELVMIWE FFGQNLKEFL HILFECLKLI NKLHKAGLTH
PF3D7_0902400       KKIPIDIWKQ FEMMQMYNGE FIENGNFVME AIVSPNICPK FYRLLYISCF ENIINIFLLR EHLKMVMISE FFGEDLENYI YIMLECLKLI NKLHQVGICH
TGME49_205250       TRLVRGAPGS GGFATVYEAT DVETNELAVK VFMEESFCYR NFSLAKFMVP SDVVMLEPAS TEVVIFLLMM RAETKVISWV FLSSQAIRLV ANVQAQGIVH
TGME49_308090       ERLKLVEPRV GDRSVVFLVR DVERLDFALK VFTGHEATFA AARLLGLLLP SDAVAVQQLS PGQSDFFLMP AASVDLELLF RLLAQLIRLA ANLQSKGLVH
TGME49_215775       YAYLEATDET GESFEVHVPY FTERPSNAIK QMKEQKQAKV HLRFIFLVKD PQKKKMIVRL DERDMFFLYP RMQSTFGEVL LLTLQVIRLL ASLHHYGLVH
TGME49_295110       GLLFQATDAT GEPMTVLVGS TWNKPGKDLD KLRQPYLANR YLRFLALVTI PGKPLVQKAK SHNEVLLLLP AIEIRFVEEL YLTVQAVRLV AHLQDEGVVH
TGME49_289050       QSFGVGARGS GGSSTTPSAK LSGGSRVQLK VCDANHSPFR LPPCILSGQE TPVSSTAPFL SCEPTVAKGP YMPPECWRIV YILRALGITA PFAQVEPLLT
TGME49_262730       PILYNRGHGS GHFGAVIKAS LDDGTLYAAK VPYQAEISSA RAELVKLVAK GLTLTETVVR RGSRLVMLLP LIDGSLVQSQ PVAREAIIAL AKLHELGFAH
TGME49_262050       SRGQSGREGR REPQMVSLRI RVGGAQVANE MQAEQKCVSE ADRIMHVSRF RFAVPLYRIR HRSALYALLN YMDVMLCDLF AAIKQLLELV AHLQALGVVH
TGME49_258580       GRYILLKKGG GGFGLVYHVE HPTTGPFALK IFVRSDLIED EFGVMKYSEL RFMVPLLRVL GKPEFYSVCA LFPKDLEEAV VCTIQMVKLL ARFHAFELVH
TGME49_286470       DDFLFHETGT GSFGRVCIVD LRGAAPMALK ILSHVEHVKD EKRILSSIEH PFIVNLLAFQ DEKRLFILME YVNGELFSHL RYAAEITLAF QYLHQRHIVY
Q13976              SDFNIIDTGV GGFGRVELVQ LKSEETFAMK ILKRQEHIRS EKQIMQGAHS DFIVRLYTFK DSKYLYMLME ACLGELWTIL RYTACVVEAF AYLHSKGIIY
P17612              DQFERIKTGT GSFGRVMLVK HKETGHYAMK ILDQIEHTLN EKRILQAVNF PFLVKLESFK DNSNLYMVME YVPGEMFSHL RYAAQIVLTF EYLHSLDLIY
TGME49_228420       EAFEFGPTGT GSFGRVKSAK YLKSTRVAVK LLKAVDHIIN EKKILLALDH PLTVRCFSFQ DSRYLYLVME LVPGEFFTHL RYAAQIVDIF DYLHSQNIIY
P31749              NEFEYLKLGK GTFGKVILVK EKATGYYAMK ILKEVAHTLT ENRVLQNSRH PFLTALKSFQ THDRLCFVME YANGELFFHL SYGAEIVSAL DYLHSKNVVY
P17252              TDFNFLMVGK GSFGKVMLAD RKGTELYAIK ILKDVECTMV EKRVLALDKP PFLTQLHCFQ TVDRLYFVME YVNGDLMYHI QYAAEISIGL FFLHKRGIIY
P05771              TDFNFLMVGK GSFGKVMLSE RKGTDLYAVK ILKDVECTMV EKRVLAPGKP PFLTQLHCFQ TMDRLYFVME YVNGDLMYHI QYAAEIAIGL FFLQSKGIIY
Q15418              DGYVVKETGV GSYSECKRCV HKATNEYAVK VIDSKRDPSE EIEILLYGQH PNIITLKVYD DGKHVYLVTE LMRGLLDKIL RVLHTIGKTV EYLHSQGVVH
Q14012              RDYDFRDVGT GAFSEVILAE DKRTQLVAIK CIAEEGSMEN EIAVLHKIKH PNIVALDIYE SGGHLYLIMQ LVSGELFDRI VLIFQVLDAV KYLHDLGIVH
P43405              KSYSFPKPGS GNFGTVKKGY YQMKKTVAVK ILKEKDELLA EANVMQQLDN PYIVRMIGIC EAESWMLVME MAELPLNKYL QLVHQVSMGM KYLEESNFVH
P00519              TDITMKHKGG GQYGEVYEGV WKKYSTVAVK TLKDVEEFLK EAAVMKEIKH PNLVQLLVCT REPPFYIITE FMTYNLLDYL RMATQISSAM EYLEKKNFIH
P12931              ESLRLEVKGQ GCFGEVWMGT WNGTTRVAIK TLKGPEAFLQ EAQVMKKLRH EKLVQLYAVV SEEPIYIVTE YMSKSLLDFL KMAAQIASGM AYVERMNYVH
P06239              ETLKLVERGA GQFGEVWMGY YNGHTKVAVK SLKGPDAFLA EANLMKQLQH QRLVRLYAVV TQEPIYIITE YMENSLVDFL KMAAQIAEGM AFIEERNYIH
P07948              ESIKLVKRGA GQFGEVWMGY YNNSTKVAVK TLKGVQAFLE EANLMKTLQH DKLVRLYVVT REEPIYIITE YMAKSLLDFL KFSAQIAEGM AYIERKNYIH
P68400              DDYQLVRKGR GKYSEVFEAI NITNNKVVVK ILKVKKKIKR EIKILELRGG PNIITLAIVK DPVSRALVFE HVNNTDFKQL YYMYEILKAL DYCHSMGIMH
P49841              VSYTDTKVGN GSFGVVYQAK LCDSGLVAIK KVLDKRFKNR ELQIMRKLDH CNIVRLRFYS SGEKKNLVLD YVPETVYRVA RYMYQLFRSL AYIHSFGICH
P27361              PRYTQLQYGE GAYGMVSSAY DHVRKRVAIK KISPCQRTLR EIQILLRFRH ENVIGIRDIL RASTLYIVQD LMETDLYKLL KFLYQILRGL KYIHSANVLH
Q16539              ERYQNLSPGS GAYGSVCAAF DTKTGRVAVK KLSPAKRTYR ELRLLKHMKH ENVIGLLVFT PARSLYLVTH LMGADLNNIV KLIYQILRGL KYIHSADIIH
Q15759              QRLQGLRPGS GAYGSVCSAY DARLRKVAVK KLSPARRTYR ELRLLKHLKH ENVIGLLVFT PATSIYLVTT LMGADLNNIV KLVYQLLRGL KYIHSAGIIH
O15264              KTYVSPTHGS GAYGSVCSAI DKRSGKVAIK KLSPAKRAYR ELLLLKHMQH ENVIGLLVFT PASSLYLVMP FMQTDLQKIM GLVYQMLKGL KYIHSAGVVH
P53778              AVYRDLQPGS GAYGAVCSAV DGRTGKVAIK KLYPAKRAYR ELRLLKHMRH ENVIGLLVFT PDETLYLVMP FMGTDLGKLM KLVYQMLKGL RYIHAAGIIH
P24941              ENFQKVEKGE GTYGVVYKAR NKLTGVVALK KIRDPSTAIR EISLLKELNH PNIVKLLVIH TENKLYLVFE FLHQDLKKFM DYLFQLLQGL AFCHSHRVLH
P20794              NRYTTMRQGD GTYGSVLMGK SNESGLVAIK RMKKECMNLR EVKSLKKLNH ANVIKLKVIR ENDHLYFIFE YMKENLYQLM KIMYQILQGL AFIHKHGFFH
```

```
                        ....|....| ....|....| ....|....| ....|....| ....|....| ....|....| ....|....| ....|....| ....|....| .
                             105        115        125        135        145        155        165        175        185
Q13554                  RDLKPENLLL ASKCKGALAF AIGFAGPEVR EDIWACGVIL YILLVGYPPF WDEDQHKLYV TPEAKNLINQ MLTINPAKRI TAHEALKHPW V
TGGT1_321650            FDIKPPNLLF PGEKGASVAF AGILRGPEMA PDVYALALTL ASFWTAAPTL KKAPEFTFLM PLLIRLKLSQ MADPEPLARV SMARFVFKAY A
PF3D7_0424500           LDISPENILI GNNCELKLCF SASYQPPECE KDKFMLGIFF IWVWNNNYIW EKFNDRKFRW PDDLKDIINQ LLPLENRAQL SLKELCKHPW W
PF3D7_1016400           LDISPQNILM SYNFEIRLCL STSCVPPECE ADKFMLGVLF IWIWNNDYLW KSEQDIDFLW PYELKKIIQK LLQTEGRKNL NLHELCAHPW W
PF3D7_1200800           LDLTAENVLI TDDYDIRLCF STSCVPIECF KDYFMLGVLF IWIWNCGHMW KTSESVNFTW PSDFKFIVKE LMNEECRKKL NLKNLMTHPW F
PF3D7_0902100           LDISPENILI GENYEMRLCF TTSYIPPECY DDIYMLGILF IWISSNRYLW GNSQNSNFKW PEGLKYIIRK LLDYESRKSL DLNELIEHPW W
PF3D7_0902400           LDFSIDNILI SKNGDMRLCF STSCIPPECE KDKYMLGILF IAIWNNGYLW YKLQDKDYLW PKELKIILRQ LLDLECRKNL NLNDLISHPW F
TGME49_205250           TDIKPANFLL LKDGRLFLGF YRPAIGPERT IDAWQLGITL YCIWCKERPT PAWDYLHFAT PELVQDLIRN LLNREPQKRM LPLQALETAA F
TGME49_308090           GRFTPENLFI MPDGRLMMGV LRVPVTREFN ANAWQLGLSI YRVWCLVLPF GLGIKRTWKV PDFVQTLTRR FLNFDRRRRL LPLEAMETPE F
TGME49_215775           TYLRPVDIVL DQRGGVFLTF VRARVVPELR TDAWALGLVI YWIWCADLPI TKLGGSEWII PQPVRALLEG FLRYPKEDRL LPLQAMETPE Y
TGME49_295110           GKILPDSFCL KREGGLYLRF VRTKVVPEVF PDAWGLGATI FFIWCFKAPT TGEYSIEFLA PENVKLLVYK LINPSVEARL LALQATETPE Y
TGME49_289050           TGRPPPPLYL KDPSQTAQLF HDVSAGAELD RDVYMLGVLL FWIWAEGAIW TCRQDAQYNW PPELRHLLKG ALDPDPTRRV TLPEILQHPW W
TGME49_262730           GDVKLNNMMI DVHGFGHMLM VRKYYLPELT QDVWALGLAI FEFVCFNLPY SLPSSLWSRS DPAVMGIVAQ FLNPNPEERP ELKFVSSYTF F
TGME49_262050           SDLKPENVLV DEAGNLFLAF AHVRCSPQTQ VDAWALGMLM YDTFCRNWPF MGNNDSDTSV ESEIKDLISR LLDIDPENRL DVLSFYMHSD F
TGME49_258580           GDVKLQNFLV DKSGLLLLSF TQPVVTPEIN IDSWMLGISL YRLWCGNFPF GMATALQVAI PEQFREMIVG FLRKTPGVRL SPQQALEQFS L
TGME49_286470           RDLKPENLLI DSQGHIKITF AKTLCGPEST RDWWALGILL FEMLAGHPPF VDLGIYRKIF DYAAKSLVKR LLTHDPLKRY GCEDVKNHKF F
Q13976                  RDLKPENLIL DHRGYAKLVF AKTFCGPEIL KDYWSLGILM YELLTGSPPF SGMKTYNIII AKNAANLIKK LCRDNPSERL GNKDIQKHKW F
P17612                  RDLKPENLLI DQQGYIQVTF AKTLCGPEIL KDWWALGVLI YEMAAGYPPF FAIQIYEKIF SSDLKDLLRN LLQVDLTKRF GNNDIKNHKW F
TGME49_228420           RDLKPENMLL DKDGYVKLTF AKTLCGPEVN KDWWTLGILI YEMMVGYPPF LDLGIYQKIF DKDAKVLVKK LLQADLSKRW GNRDIKECRW F
P31749                  RDLKLENLML DKDGHIKITF CKTFCGPEVE NDWWGLGVVM YEMMCGRLPF YNEKLFELIL GPEAKSLLSG LLKKDPKQRL GGKEIMQHRF F
P17252                  RDLKLDNVML DSEGHIKIAF CKTFCGPEIA QDWWAYGVLL YEMLAGQPPF DGDELFQSIL SKEAVSVCKG LMTKHPAKRL GCRDVREHAF F
P05771                  RDLKLDNVML DSEGHIKIAF CKTFCGPEIA QDWWAFGVLL YEMLAGQAPF EGDELFQSIM SKEAVAICKG LMTKHPGKRL GCRDIKEHAF F
Q15418                  RDLKPSNILE SGNPELRICF AKTPCYPEVK QDIWSLGILL YTMLAGYTPF ANDTPEEILV SETAKDLVSK MLHVDPHQRL TAKQVLQHPW V
Q14012                  RDLKPENLLL DEDSKIMISF SKTACGPEVA KDCWSIGVIA YILLCGYPPF YDAKLFEQII SDSAKDFIRH LMEKDPEKRF TCEQALQHPW I
P43405                  RDLAARNVLL VTQHYAKISF SKAQTHPECN YDVWSFGVLM WEAFSYGKPY RGMKGSEVTC PREMYDLMNL CWTYDVENRP GFVELRLRNY Y
P00519                  RDLAARNCLV GENHLVKVAF SRAHAGPESA NDVWAFGVLL WEIATYGSPY PGSQVYELLC PEKVYELMRA CWQWNPSDRP SFHQAFETMF Q
P12931                  RDLRAANILV GENLVCKVAF ARARQGPEAL GDVWSFGILL TELTTKGVPY PGREVLDQVC PESLHDLMCQ CWRKEPEERP TFLQAFLEDY F
P06239                  RDLRAANILV SDTLSCKIAF ARAREGPEAN GDVWSFGILL TEIVTHGIPY PGPEVIQNLC PEELYQLMRL CWKERPEDRP TFLRSVLEDF F
P07948                  RDLRAANVLV SESLMCKIAF ARAREGPEAN GDVWSFGILL YEIVTYGIPY PGADVMTALC PDELYDIMKM CWKEKAEERP TFLQSVLDDF Y
P68400                  RDVKPHNVMI DHEHRLRLIW AEVRVAPELD QDMWSLGCML ASMIFRKPFF HGYDQLVRIV SPEALDFLDK LLRYDHQSRL TAREAMEHPY F
P49841                  RDIKPQNLLL DPDTALKLCF AKSYICPELG TDVWSAGCVL AELLLGQPIF PGVDQLVEIT PPEAIALCSR LLEYTPTARL TPLEACAHSF F
P27361                  RDLKPSNLLI NTTCDLKICF AREYVAPEIN KDIWSVGCIL AEMLSNRPIF PGLDQLNHIS DSKALDLLDR MLTFNPNKRI TVEEALAHPY L
Q16539                  RDLKPSNLAV NEDCELKILF ARGYVAPEIN MDIWSVGCIM AELLTGRTLF PGIDQLKLIA NPLAVDLLEK MLVLDSDKRI TAAQALAHAY F
Q15759                  RDLKPSNVAV NEDCELRILF ARGYVAPEIN MDIWSVGCIM AELLQGKALF PGIDQLKRIA NPLAIDLLGR MLVLDSDQRV SAAEALAHAY F
O15264                  RDLKPGNLAV NEDCELKILF ARGYVVPEVS MDIWSVGCIM AEMLTGKTLF KGLDQLTQIA SPQAADLLEK MLELDVDKRL TAAQALTHPF F
P53778                  RDLKPGNLAV NEDCELKILF ARGYVVPEVN MDIWSVGCIM AEMITGKTLF KGLDQLKEIA SPLAVNLLEK MLVLDAEQRV TAGEALAHPY F
P24941                  RDLKPQNLLI NTEGAIKLAF ARHEVVPEIG KDIWSLGCIF AEMVTRRALF PGIDQLFRIL DEDGRSLLSQ MLHYDPNKRI SAKAALAHPF F
P20794                  RDMKPENLLC MGPELVKIAF ARDYVSPEVR SDVWAVGSIM AELYMLRPLF PGVDEIFKIA SNEAIQLMTE MLNWDPKKRP TASQALKHPY F
```
