# Supplementary material for: Structural insights into an atypical secretory pathway kinase crucial for Toxoplasma gondii invasion
Source: Nat Commun. 2021 Jun 18;12:3788. doi: 10.1038/s41467-021-24083-y (PMC8213820; doi:10.1038/s41467-021-24083-y)
Supplement: Supplementary file 14 — Reporting Summary [file 41467_2021_24083_MOESM14_ESM.pdf]

# nature research

Corresponding author(s): Dominique Soldati-Favre

Last updated by author(s): May 11, 2021

# Reporting Summary

Nature Research wishes to improve the reproducibility of the work that we publish. This form provides structure for consistency and transparency in reporting. For further information on Nature Research policies, see our Editorial Policies and the Editorial Policy Checklist.

## Statistics

For all statistical analyses, confirm that the following items are present in the figure legend, table legend, main text, or Methods section.

| n/a | Confirmed | |
|---|---|---|
| ☐ | ☒ | The exact sample size ($n$) for each experimental group/condition, given as a discrete number and unit of measurement |
| ☐ | ☒ | A statement on whether measurements were taken from distinct samples or whether the same sample was measured repeatedly |
| ☐ | ☒ | The statistical test(s) used AND whether they are one- or two-sided<br>*Only common tests should be described solely by name; describe more complex techniques in the Methods section.* |
| ☒ | ☐ | A description of all covariates tested |
| ☒ | ☐ | A description of any assumptions or corrections, such as tests of normality and adjustment for multiple comparisons |
| ☐ | ☒ | A full description of the statistical parameters including central tendency (e.g. means) or other basic estimates (e.g. regression coefficient) AND variation (e.g. standard deviation) or associated estimates of uncertainty (e.g. confidence intervals) |
| ☒ | ☐ | For null hypothesis testing, the test statistic (e.g. $F$, $t$, $r$) with confidence intervals, effect sizes, degrees of freedom and $P$ value noted<br>*Give P values as exact values whenever suitable.* |
| ☒ | ☐ | For Bayesian analysis, information on the choice of priors and Markov chain Monte Carlo settings |
| ☒ | ☐ | For hierarchical and complex designs, identification of the appropriate level for tests and full reporting of outcomes |
| ☒ | ☐ | Estimates of effect sizes (e.g. Cohen's $d$, Pearson's $r$), indicating how they were calculated |

*Our web collection on statistics for biologists contains articles on many of the points above.*

## Software and code

Policy information about availability of computer code

| | |
|---|---|
| Data collection | For FIB-SEM experiments : iTEM acquisition software (Olympus Soft-Imaging Systems; version 5.2); AutoSlice and View G3 software (FEI; version 4.0)<br>Cell impedance was collected using the RTCA software (Agilent; version 1.0) |
| Data analysis | Mascot (Matrix science; version 2.5.1); Scaffold (Proteome software; version 4.9.0); Image J (NIH, version 1.53c); GraphPad Prism (GraphPad Software; version 8.0.1); FlowJo (Becton, Dickinson & Company; version 9.0); Kaluza (Beckman Coulter; version 2.1); Blender software (blender.org; version 2.79); LasX Software (Leica, version 3.7.0), phenix.map_to_model (Phenix; version 1.16-3549); MaxQuant (Max Planck Institute; version 1.6.2.3); PhosphoSitePlus (www.phosphosite.org, version 6.5.9.3); Ilastik software (ilastik.org; version 1.2.0); MUSCLE sequence alignment software (EMBL-EBI; version 3.8.31) ; PhyML 3.0 (ATGC; version 3.0); PyMol (DeLano Scientific LLC; version 4.2.4); UCSF Chimera (UCSF; version 1.13.1); Gctf (version 1.06); MotionCor2 (UCSF; version 1.3.0); Coot (version 0.9.5); MolProbity (version 4.5.1); Andromeda search engine (MaxQuant; version 1.6.2.3); HD examiner software (Sierra Analytics; version 1.4); R package SRMService (version 0.1.9.23); R package limma (limma; version.3.40.6); R package funscoR (version 0.1.0) |

For manuscripts utilizing custom algorithms or software that are central to the research but not yet described in published literature, software must be made available to editors and reviewers. We strongly encourage code deposition in a community repository (e.g. GitHub). See the Nature Research guidelines for submitting code & software for further information.

## Data

Policy information about availability of data

All manuscripts must include a data availability statement. This statement should provide the following information, where applicable:

- Accession codes, unique identifiers, or web links for publicly available datasets
- A list of figures that have associated raw data
- A description of any restrictions on data availability

All data are present in the main text and the supplementary materials. Source data are provided with this paper. The Figures 1, 2, 3, 5, 6, 7, 8, 9 and the Supplementary Figures 1, 2, 5, 6, 7, 8, 10 are associated with raw data presented in the Source Data File. The mass spectrometry proteomics regarding RON4 immunoprecipitation have been deposited on the ProteomeXchange Consortium via the PRIDE62 partner repository with the dataset identifier PXD021516 [http://www.ebi.ac.uk/pride/archive/projects/PXD021516]. The phosphoproteomic data have been deposited on the ProteomeXchange Consortium via the PRIDE 62 partner repository with the dataset identifier PXD018056 [https://www.ebi.ac.uk/pride/archive/projects/PXD018056]. The HDX-MS data have been deposited on the ProteomeXchange Consortium via the PRIDE62 partner repository with the dataset identifier PXD023791 [http://www.ebi.ac.uk/pride/archive/projects/PXD023791]. The coordinates and the cryo-EM map have been deposited to the Protein Data Bank (pdb id: 7NUR; [http://doi.org/10.2210/pdb7NUR/pdb]) and Electron Microscopy Data Bank (accession code: EMD-12600; [https://www.ebi.ac.uk/pdbe/entry/emdb/EMD-12600]). The public dataset used for mass-spectrometry analysis of RON4 immunoprecipitation is the ToxoDB_Tgondii_GT1 database (ToxoplasmaDB.org, release 42) and the one used for the phosphoproteomics analsyis is the ToxoDB_Tgondii_GT1 database (ToxoplasmaDB.org, release 44). All biological materials and data are available from the authors upon request.

# Field-specific reporting

Please select the one below that is the best fit for your research. If you are not sure, read the appropriate sections before making your selection.

☒ Life sciences ☐ Behavioural & social sciences ☐ Ecological, evolutionary & environmental sciences

For a reference copy of the document with all sections, see nature.com/documents/nr-reporting-summary-flat.pdf

# Life sciences study design

All studies must disclose on these points even when the disclosure is negative.

| | |
|---|---|
| Sample size | Sample size were chosen based on previously published litterature for the performed assays (1,2,3). All experiments were performed with enough biological replicates (a minimum of 3) to allow relevant statistical analysis.<br>1. Guérin, A. et al. Efficient invasion by Toxoplasma depends on the subversion of host protein networks. Nat. Microbiol. 2, 1358–1366 (2017).<br>2. Treeck, M., Sanders, J. L., Elias, J. E. & Boothroyd, J. C. The Phosphoproteomes of Plasmodium falciparum and Toxoplasma gondii reveal unusual adaptations within and beyond the parasites' boundaries. Cell Host Microbe 10, 410–419 (2012).<br>3. Dogga, S. K. et al. A druggable secretory protein maturase of Toxoplasma essential for invasion and egress. Elife 6, 1–35 (2017). |
| Data exclusions | No data were excluded from the analysis presented in this study. |
| Replication | All experiments were done with biological replicates and take into account the biological variation of the system. At least three biological replicates and two technical replicates were performed. All results were successfully replicated. |
| Randomization | Assignement of strains to treatment group were randomized in the study to get non-bias response. |
| Blinding | For quantification based on IFA, the sample labeling was hidden during data collection and only revealed post-analysis. For the other experiements, investigators were not blinded. The analysis perfomed have quantitative endpoints and are not subjected to investigator bias. |

# Reporting for specific materials, systems and methods

We require information from authors about some types of materials, experimental systems and methods used in many studies. Here, indicate whether each material, system or method listed is relevant to your study. If you are not sure if a list item applies to your research, read the appropriate section before selecting a response.

## Materials & experimental systems

| n/a | Involved in the study |
|---|---|
| ☐ | ☒ Antibodies |
| ☐ | ☒ Eukaryotic cell lines |
| ☒ | ☐ Palaeontology and archaeology |
| ☐ | ☒ Animals and other organisms |
| ☒ | ☐ Human research participants |
| ☒ | ☐ Clinical data |
| ☒ | ☐ Dual use research of concern |

## Methods

| n/a | Involved in the study |
|---|---|
| ☒ | ☐ ChIP-seq |
| ☐ | ☒ Flow cytometry |
| ☒ | ☐ MRI-based neuroimaging |

# Antibodies

Antibodies used

Primary antibodies used in this study include: anti-RON2; anti-RON4 (rb); anti-RON8 (a gift from Dr. M. Lebrun), anti-RON4 (ms); anti-ROP2/3/4; anti-ROP1; anti-RON9 (a gift from Dr. JF. Dubremetz), anti-RON5C (a gift from Dr. P. Bradley), anti-Ty (rb) (a gift from Dr. CJ. Tonkin), anti-MIC2 (a gift from Dr. V. Carruthers), anti-Phospho-STAT6 (Cell signalling #9361), anti-myc (ms) (DSH Bishop #9E10), anti-Myc (rb) (Sigma #3956), anti-GRA1 (Anawa), anti-GFP (Roche #11814460001), anti-RON13 produced in this study, anti-ARO [6], anti-GAP45 [7], anti SAG1 (ms) [8], anti-SAG1 (ascite) [9], anti-actin [10], anti-catalase [11], anti-α + β tubulin [12], anti-Ty (ms) [13], anti-HA (rat) (Roche clone 3F10), anti-HA (ms) (Clone 12CA5).

Validation

Validation of RON13 antibody is described in this study.
The other antibody used were validated in the following publications:
anti-RON2; anti-RON4 (rb) : Besteiro, S., Dubremetz, J. F. & Lebrun, M. The moving junction of apicomplexan parasites: A key structure for invasion. Cell. Microbiol. 13, 797–805 (2011).
anti-RON4 (ms); anti-RON9 : Leriche, M. A. & Dubremetz, J. F. Characterization of the protein contents of rhoptries and dense granules of Toxoplasma gondii tachyzoites by subcellular fractionation and monoclonal antibodies. Mol. Biochem. Parasitol. 45, 249–259 (1991).
anti-RON5C; anti-RON8 : Straub, K. W., Cheng, S. J., Sohn, C. S. & Bradley, P. J. Novel components of the Apicomplexan moving junction reveal conserved and coccidia-restricted elements. cell Microbiol. 11, 590–603 (2009).
anti-ROP2/3/4 : Sadak, A., Taghy, Z., Fortier, B. & Dubremetz, J. Characterization of a family of rhoptry proteins of Toxoplasma gondii. Mol. Biochem. Parasitol. 29, 203–211 (1988).
anti-ARO : Mueller, C. et al. The toxoplasma protein ARO mediates the apical positioning of rhoptry organelles, a prerequisite for host cell invasion. Cell Host Microbe 13, 289–301 (2013).
anti-GAP45 : Frenal, K., Polonais, V., Marq, J., Stratmann, R. & Limenitakis, J. Functional Dissection of the Apicomplexan Glideosome Molecular Architecture. Cell Host Microbe 343–357 (2010) doi:10.1016/j.chom.2010.09.002.
anti-SAG1 (ms) : Couvreur, G., Sadak, A., Fortier, B. & Dubremetz, J. F. Surface antigens of Toxoplasma gondii. Parasites and Vectors 97, 1–10 (1988).
anti-SAG1 (ascite) : Kim, K., Soldati, D. & Boothroyd, J. Gene replacement in Toxoplasma gondii with chloramphenicol acetyltransferase as selectable marker. Science (80-. ). 262, 911–914 (1993).
anti-actin : Herm-gotz, A. et al. Toxoplasma gondii myosin A and its light chain : a fast , single-headed , plus-end-directed motor. EMBO J. 21, 2149–2158 (2002).
anti-catalase : Ding, M., Clayton, C., Soldati, D., Biologie, M. & Feld, I. N. Toxoplasma gondii catalase : are there peroxisomes in Toxoplasma ? J. Cell Sci. 2419, 2409–2419 (2000).
anti-Ty (ms) : Bastin, P., Bagherzadeh, A., Matthews, K. R. & Gull, K. A novel epitope tag system to study protein targeting and organelle biogenesis in Trypanosoma brucei. Mol. Biochem. Parasitol. 77, (1996).
anti-Ty (rb) : Uboldi, A. D. et al. Regulation of Starch Stores by a Ca2+-Dependent Protein Kinase Is Essential for Viable Cyst Development in Toxoplasma gondii. Cell Host Microbe 18, 670–681 (2015).
anti-α + β tubulin : Tosetti, N. et al. Essential function of the alveolin network in the subpellicular microtubules and conoid assembly in Toxoplasma gondii. Elife 9, 1–22 (2020).
anti-Phospho-STAT6 (Cell signalling #9361) : Validated for Western-blots and IFA
anti-myc (ms) (DSH Bishop #9E10) : Validated for Western-blots and IFA
anti-Myc (rb) (Sigma #3956) : Validated for Western-blots and IFA
anti-GRA1 (Anawa) : Validated for Western-blots and IFA
anti-GFP (Roche #11814460001) : Validated for Western-blots and IFA
anti-HA (rat) (Roche clone 3F10) : Validated for Western-blots and IFA
anti-HA (ms) (Clone 12CA5) : Validated for Western-blots and IFA

# Eukaryotic cell lines

Policy information about cell lines

Cell line source(s)

human foreskin fibroblasts (HFFs, ATCC, CRL 1634), HeLa cells (ATCC, CCL-2)
T. gondii ΔKU80 RH strain (Huynh, M. H. & Carruthers, V. B. Tagging of endogenous genes in a Toxoplasma gondii strain lacking Ku80. Eukaryot. Cell 8, 530–539 (2009))
T. gondii TaTi/ΔKU80 strain (Meissner, M., Schlüter, D. & Soldati, D. Role of Toxoplasma gondii Myosin A in Powering Parasite Gliding and Host Cell Invasion. Science (80-. ). 298, 837 LP – 840 (2002))

| | |
|---|---|
| Authentication | none of this cell lines were authentificated |
| Mycoplasma contamination | HFF, HeLa and parental strains ΔKU80 RH and TaTi/ΔKU80 have been tested for mycoplasma contamination. Following transfection, the resulting transgenic T. gondii lines have not been tested for mycoplasma contamination except prior to use them for the virulence experiments. |
| Commonly misidentified lines (See ICLAC register) | None to be reported |

## Animals and other organisms

Policy information about studies involving animals; ARRIVE guidelines recommended for reporting animal research

| | |
|---|---|
| Laboratory animals | Mouse 7 weeks old female CD1 mice (Charles River), day/night cycle of 12h/12h. The temperature of the room and humidity are set to 22C and 35% humidity respectively. |
| Wild animals | no wild animals were used in this study |
| Field-collected samples | no field collected samples were used in the study |
| Ethics oversight | All animal experiments were conducted with the authorization numbers GE121-19, according to the guidelines and regulations issued by the Swiss Federal Veterinary Office. |

Note that full information on the approval of the study protocol must also be provided in the manuscript.

## Flow Cytometry

### Plots

Confirm that:

- [x] The axis labels state the marker and fluorochrome used (e.g. CD4-FITC).
- [x] The axis scales are clearly visible. Include numbers along axes only for bottom left plot of group (a 'group' is an analysis of identical markers).
- [x] All plots are contour plots with outliers or pseudocolor plots.
- [x] A numerical value for number of cells or percentage (with statistics) is provided.

### Methodology

| | |
|---|---|
| Sample preparation | Human Foreskin fibroblasts (ATCC, CRL 1634) infected with Toxoplasma gondii expressing beta-lactamase. The infected cells were incubated with CCF4-AM (ThermoFisher Scientific) or DMSO prior trypsinization and analysis. |
| Instrument | Gallios flow cytometer (Beckman Coulter) |
| Software | FlowJo (Becton, Dickinson & Company; version 9.0); Kaluza (Beckman Coulter; version 2.1) |
| Cell population abundance | Intact infected cells represent ~ 50% of the total abundance determined by SSC/FSC. All intact cell were analyzed. |
| Gating strategy | All intact cells were analyzed. The SSC/FSC was used to discriminate intact fibroblasts from debris and extracellular parasites. |

- [x] Tick this box to confirm that a figure exemplifying the gating strategy is provided in the Supplementary Information.
